# Deep eutectic solvent-catalyzed arylation of benzoxazoles with aromatic aldehydes†

**DOI:** 10.1039/c8ra01094c

**Published:** 2018-03-20

**Authors:** Phuong Hoang Tran, Anh-Hung Thi Hang

**Affiliations:** Department of Organic Chemistry, Faculty of Chemistry, University of Science, Viet Nam National University Ho Chi Minh City 721337 Viet Nam thphuong@hcmus.edu.vn

## Abstract

A novel and efficient methodology for the arylation of benzoxazoles with aromatic aldehydes catalyzed by deep eutectic solvent has been developed. The reaction smoothly proceeded with a wide range of substrates to give the desired products in high yields within short reaction time. Deep eutectic solvents are easily recovered and reused without significant loss of catalytic activity.

## Abstract 2

A green and efficient synthesis of 2-arylbenzoxazoles using deep eutectic solvent as a homogeneous catalyst has been developed for the first time.

## Graphical abstract

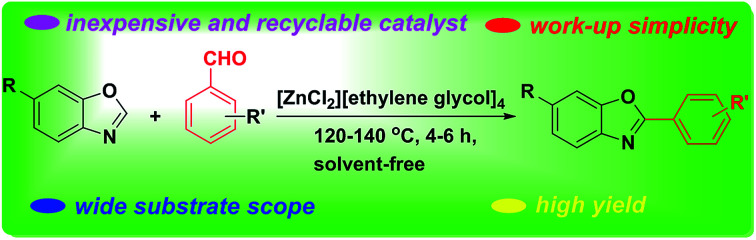

## Introduction

2-Arylbenzoxazoles are important structural subunits in natural products, pharmaceuticals, agrochemicals, and dyes.^1–4^ Therefore, the development of efficient synthetic methods for C2-arylation of benzoxazoles has received much attention.^5,6^ Among them, palladium-catalyzed cross-coupling is one of the most powerful and reliable tools to achieve aryl-substituted benzoxazoles through direct C–H activation and subsequent C–C bond formation.^7–9^ However, this protocol still suffered from inherent drawbacks associated with the use of uncommon, toxic, and expensive agents such as aryltrimethylammonium triflates,^7^ aryl halides or triflates,^10–17^ aryl boronic acids,^18^ sodium arylsulfinates,^19^ arylsulfonyl hydrazides,^20^ and 3-phenylpropionic acid.^21^ Additionally, the requirement of high-cost palladium catalysts and other expensive additives also prevents the palladium-catalyzed cross-coupling procedure from being employed in large-scale synthesis.^22^ Recently, the replacement of palladium by other transition metals, such as bis(diisopropylphosphinomethyl)amine nickel(ii) and nickel(0) complexes,^23^ metal–organic frameworks,^24,25^ Cu/Fe system,^26^ CeO_2_/Fe_3_O_4_,^27^ CuCN(PPh_3_)_2_,^28^ and Ni(COD)_2_,^29^ has been investigated. These direct arylation methods have received much attention due to their advantage of avoiding the use of stoichiometric amounts of expensive organometallic reagents or additives. However, these protocols still encounter several problems including low yields, long reaction times, poor substrate scope, and high toxicity. Therefore, the development of an effective alternative method for the arylation of benzoxazoles remains highly desirable. Aromatic aldehydes are considered as preferable reagents in the arylation of benzoxazoles owing to their widespread availability, non-toxicity, and low-cost production. The reaction between benzoxazoles and aromatic aldehydes can be conducted in the presence of a suitable catalyst, *e.g.*, molecular I_2_ or FeSO_4_.^30,31^ Until now, there has been no further report on the arylation of benzoxazoles using aldehydes as reagents.

In an attempt to develop a cost-effective and environmentally benign protocol, we focus on exploring a new and affordable catalyst for direct arylation of benzoxazoles under “greener” conditions. Deep eutectic solvents (DESs) which were discovered for the first time by Abbott in 2001 have been known as a new class of ionic liquids possessing many outstanding characteristics.^32–34^ DESs have found many applications as green solvents in diverse fields including nanotechnology,^35^ separation processes,^36^ transition metal catalyzed reactions,^37^ material chemistry,^38^ stabilization of DNA,^39^ and organic synthesis.^40^ Besides, DESs have been known as preferable alternative solvents/catalysts for organic synthesis due to their non-hazardous, non-toxic, stable, non-flammable, and inexpensive nature.^41–49^ Recently, we have reported DESs-catalyzed organic transformations such as Friedel–Crafts acylation and esterification of sterically hindered alcohols.^50,51^ As our ongoing efforts to develop environmentally benign syntheses, it is the aim of this communication to describe our preliminary results in the arylation of benzoxazoles with aromatic aldehyde using DES as a green catalyst. Notable features of our report include: (i) straightforward and affordable preparation of catalyst, (ii) simple work-up, (iii) removable additive agents, (iv) recyclable, biodegradable, and low-toxic catalyst.

## Results and discussion

### Preparation and characterization of [ZnCl_2_][ethylene glycol]_4_

DESs were synthesized by mixing zinc chloride and ethylene glycol (HBDs) at a defined molar ratio (1 : 4) and heating at 120 °C for 1 h at atmospheric pressure under constant magnetic stirring until a homogeneous liquid was formed. The charge delocalization which occurs through hydrogen bonding between the halide anion and ethylene glycol is responsible for the decrease in the *T*_m_ of the mixture.^52,53^

**Fig. 1:**
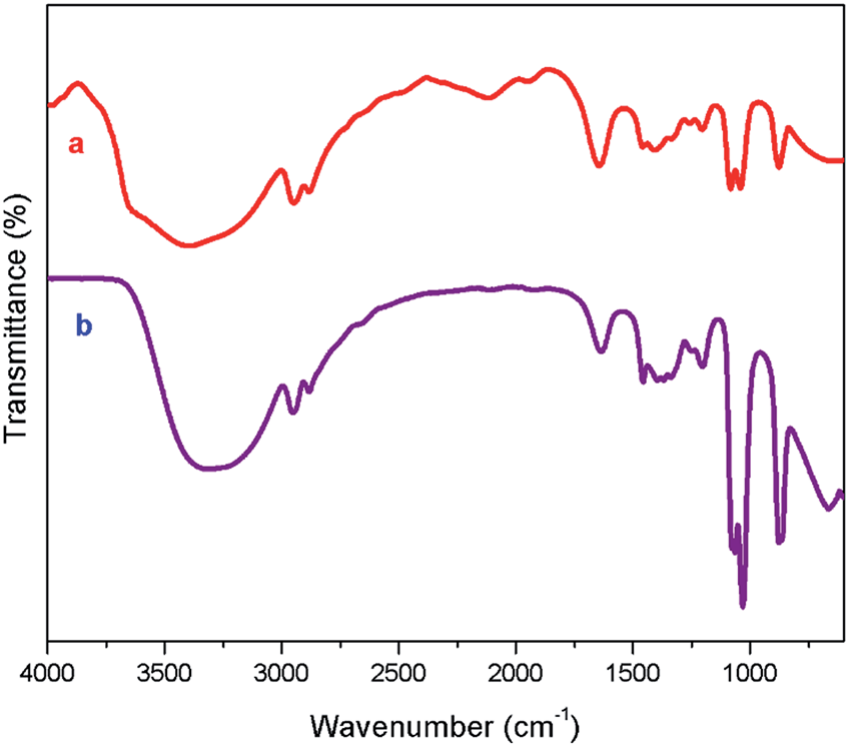
FT-IR spectra of ethylene glycol (a), and [ZnCl_2_][ethylene glycol]_4_ (b).

The NMR characterization are in good agreement with the structure of [ZnCl_2_][ethylene glycol]_4_ and the NMR spectra show that the catalyst is free of impurities. DESs with high viscosity and the inter- as well as intra-dipolar interactions can cause the broadening effect on the resonance signals of NMR spectrum.^54^ The high viscosity of DES used in the current work involves the formation of massive hydrogen bond network between each component.^55^Fig. 1 displays the FT-IR spectra of ethylene glycol and [ZnCl_2_][ethylene glycol]_4_. The spectrum of the [ZnCl_2_][ethylene glycol]_4_ is an overlap of those of ethylene glycol. The result showed that the structure of ethylene glycol was not destroyed in the [ZnCl_2_][ethylene glycol]_4_. Particularly, the absorption bands of ethylene glycol at 3390 cm^−1^ could be ascribed to stretch vibration of O–H functional group. As observed in Fig. 1, the O–H stretching vibration of [ZnCl_2_][ethylene glycol]_4_ shifts to lower wavenumber, indicating that O–H of ethylene glycol takes part in the formation of the hydrogen bond with the anion of zinc chloride.^47,53,55^

**Fig. 2:**
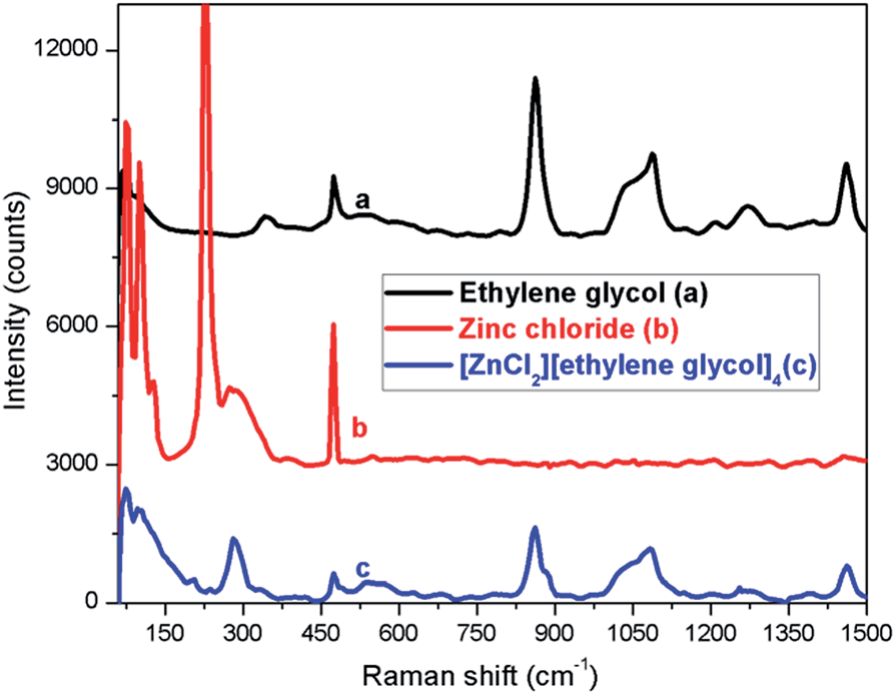
Raman spectra of ethylene glycol (a), zinc chloride (b), and [ZnCl_2_][ethylene glycol]_4_ (c).

The Raman spectra of ethylene glycol, zinc chloride, and [ZnCl_2_][ethylene glycol]_4_ are presented in Fig. 2 for a comparative analysis in the region from 50 to 1500 cm^−1^. In pure ZnCl_2_, we have observed a strong signal at 225 cm^−1^ and another weak signal at 290 cm^−1^; however, at low ZnCl_2_ molar fraction (in deep eutectic solvent) the feature at 290 cm^−1^ becomes strong and the signal at 225 cm^−1^ disappears. Rubim *et al.* have also observed the same feature for ZnCl_2_ in eutectic mixture with 1-butyl-3-methylimidazolium chloride.^56^ Thus, the Raman spectrum of [ZnCl_2_][ethylene glycol]_4_ does not change as compared with the signal of ethylene glycol but additional peaks from ZnCl_2_ appear at 80 and 290 cm^−1^.

**Fig. 3:**
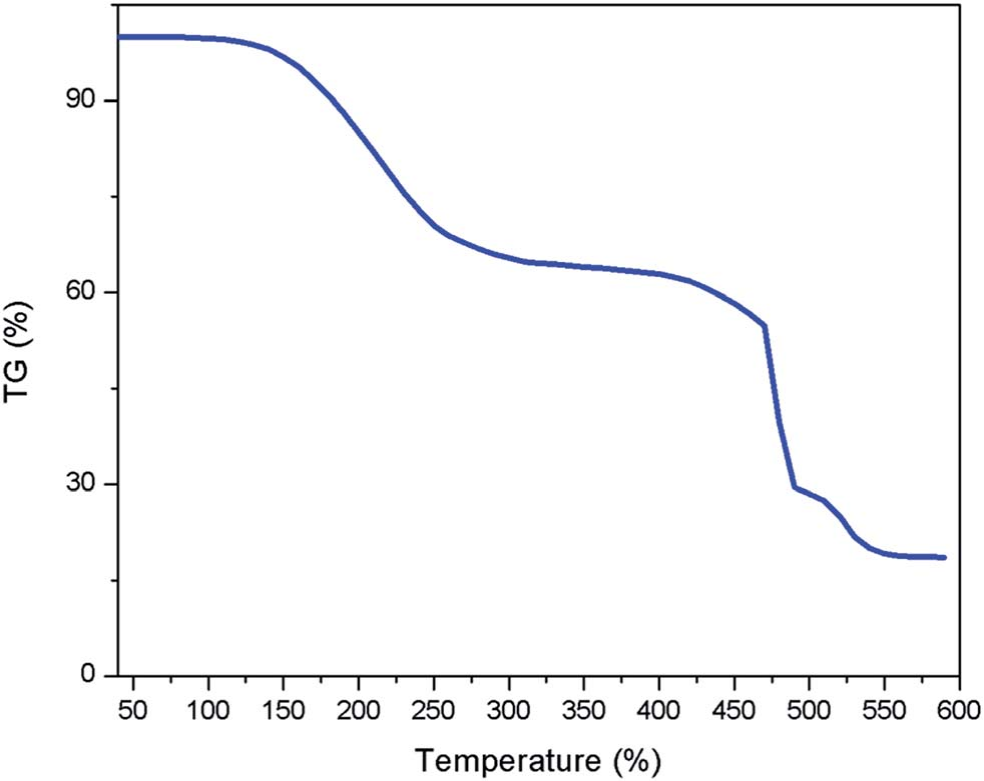
TG analysis of [ZnCl_2_][ethylene glycol]_4_.

Thermal gravimetric analysis (TGA) of [ZnCl_2_][ethylene glycol]_4_ was performed in Fig. 3. The major weight loss occurs in the temperature range from 200 °C to 475 °C, which make the [ZnCl_2_][ethylene glycol]_4_ suitable for high-temperature reaction conditions.

### Optimization of reaction conditions

We initiated our studies by investigating various reaction conditions for the C2-arylation of benzoxazole by benzaldehyde (Tables 1 and 2). The optimized condition revealed that the air-stable and environmentally benign [ZnCl_2_][ethylene glycol]_4_ proved to be an effective catalyst for the highly selective C2-arylation of benzoxazole with benzaldehyde in 2 : 1 molar ratio. The method provided the desired product in high yield in the absence of other additives such as organic solvents or bases. It is also noteworthy that this arylation of benzoxazole proceeded smoothly without the need of inert atmosphere.

**Table 1:** Optimization of catalyst for the arylation of benzoxazole^a^

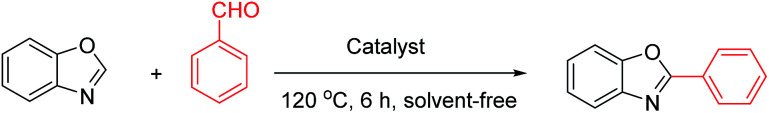		
Entry	Catalyst	Yield^b^ (%)
1	ZrCl_4_	70
2	ZnCl_2_	20
3	FeCl_3_	75
4	HfCl_4_	73
5	[ZnCl_2_][ethylene glycol]_4_	95
6	[ChCl][ZnCl_2_]_3_	0
7	[ZnCl_2_]_4_[urea]	80
8	[ZnCl_2_][glycerol]_4_	85
9	[ZnCl_2_][acetamide]_4_	5

a Reaction conditions: benzoxazole (1 mmol), benzaldehyde (0.5 mmol), solvent-free.

b Isolated yield.

**Table 2:** Optimization of the reaction conditions for the synthesis of 2-phenylbenzoxazole^a^

Entry	Catalytic amount (mol%)	Time (h)	Temperature (°C)	Yield^b^ (%)
1	1	6.0	120	60
2	2	6.0	120	75
3	3	6.0	120	80
4	5	6.0	120	95
5	10	6.0	120	95
6	5	1.0	120	0
7	5	1.5	120	0
8	5	2.5	120	0
9	5	4.0	120	30
10	5	4.5	120	75
11	5	6.0	120	95
12	5	6.5	120	97
13	5	6.0	90	40
14	5	6.0	100	45
15	5	6.0	110	80
16	5	6.0	120	95
17	5	6.0	130	95
18	5	6.0	140	95

a Reaction conditions: benzoxazole (1 mmol), benzaldehyde (0.5 mmol), solvent-free.

b Isolated yield.

With the optimized catalyst in hand, the scope of the benzoxazoles and aromatic aldehydes in the arylation reaction was studied (Table 3). The results demonstrated that the developed pathway provided the C2-arylation products in high yields. Generally, the robust DES between ethylene glycol and zinc chloride allowed a variety of substituted benzoxazoles and aldehydes to transform into the desired products in good to excellent yields with 100% selectivity in C2-arylation. First, a large number of aromatic aldehydes, regardless of containing electron-donating substituents (methyl, *t*-butyl, hydroxy, methoxy) or electron-withdrawing substituents (nitro, halide), can react with benzoxazoles to produce the expected products under the given reaction conditions (Table 3). However, low yields of the desired products were observed for benzaldehydes bearing severe electron-withdrawing groups such as nitro or fluoro substituents (Table 3, entries 5, 6, 8, 10, 18, 26, 35). Next, the scope of various benzoxazoles was also evaluated. As our expectation both 5-methylbenzoxazole and 5-chlorobenzoxazole generally underwent the arylation to give the corresponding products in very good yields ranging from 82 to 95% except the case of 4-fluorobenzaldehyde and 4-hydroxybenzaldehyde whose resulting arylated products were only obtained in significantly diminished yields of 70–75% (Table 3, entries 14–30). For the benzoxazole bearing a nitro substituent, the lower yields of arylation products with various aldehydes were recorded even though harsher conditions (higher temperature for prolonged reaction time) were employed (Table 3, entries 31–37). It is noteworthy that the condensation between benzoxazole and benzaldehyde was successfully performed on a 10 mmol or 20 mmol scale, and the yield is virtually the same as on 1 mmol scale (Table 3, entry 1).

**Table 3:** The scope of arylation of benzoxazoles with aromatic aldehydes

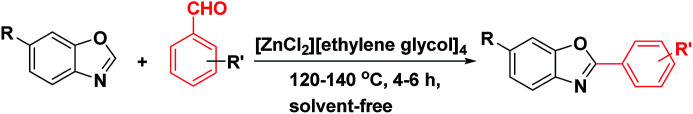			
Entry	Product	Condition	Yield^a^ (%)
1	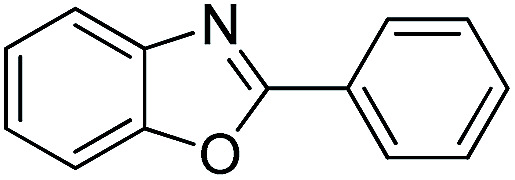	120 °C, 6 h	95 (94)^b^
2	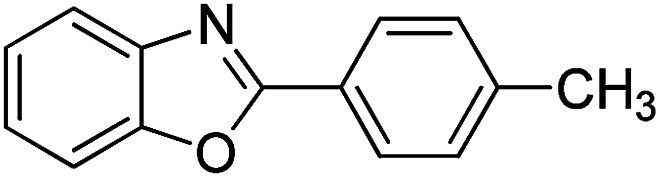	120 °C, 5 h	93
3	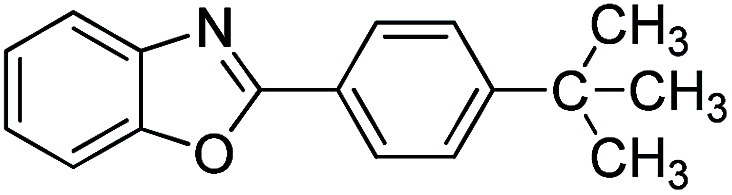	120 °C, 5 h	94
4	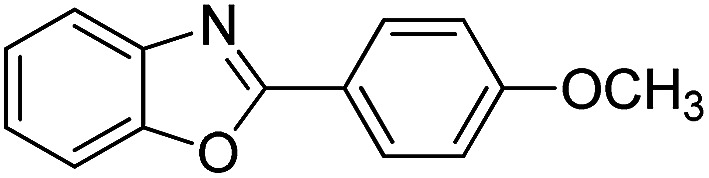	120 °C, 4 h	95
5	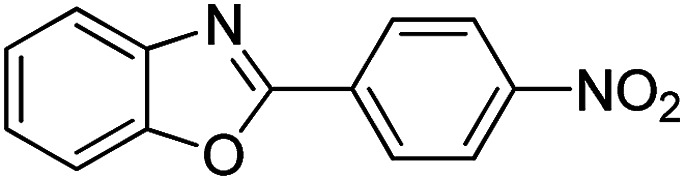	140 °C, 6 h	75
6	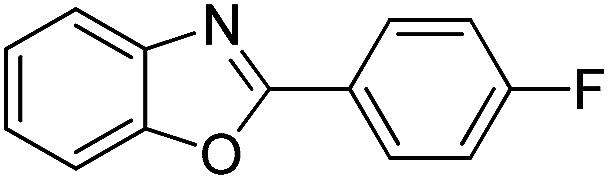	120 °C, 4 h	80
7	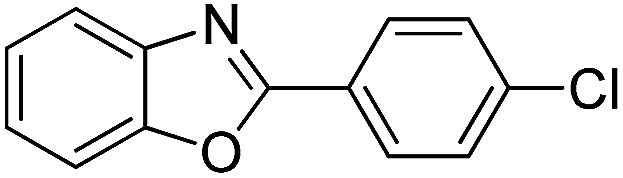	120 °C, 4 h	92
8	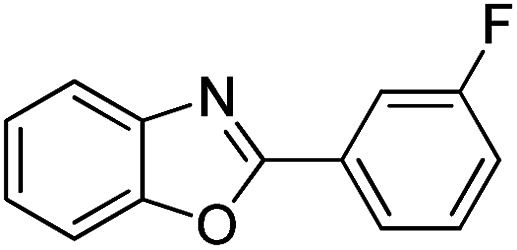	120 °C, 5 h	70
9	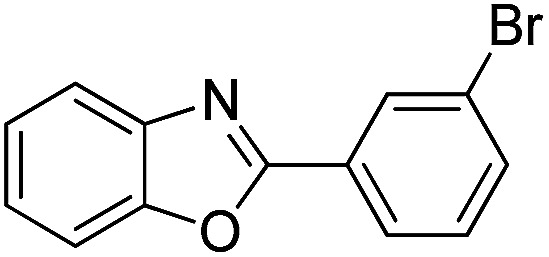	120 °C, 5 h	90
10	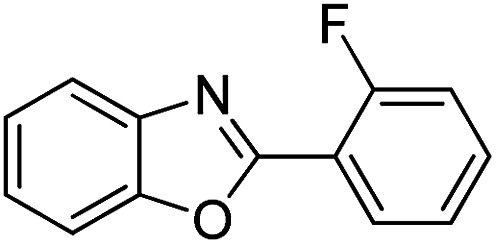	120 °C, 5 h	72
11	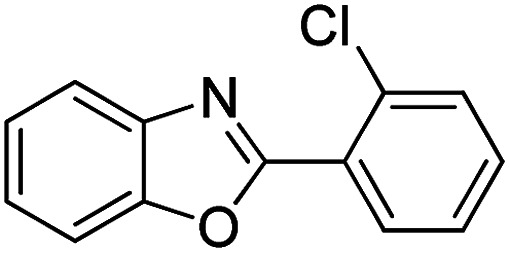	120 °C, 4 h	90
12	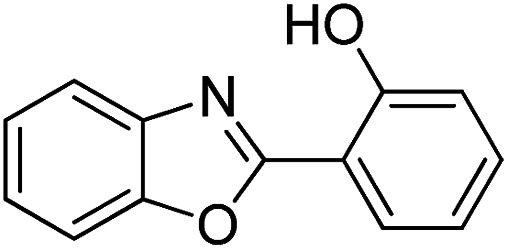	120 °C, 6 h	93
13	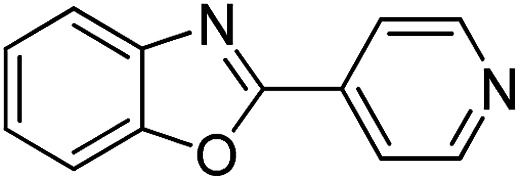	120 °C, 6 h	80
14	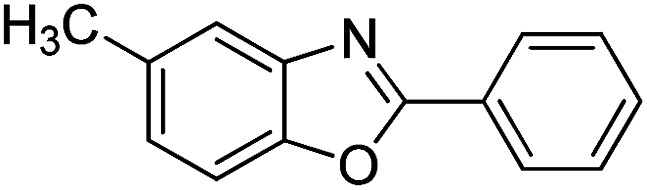	120 °C, 5 h	94
15	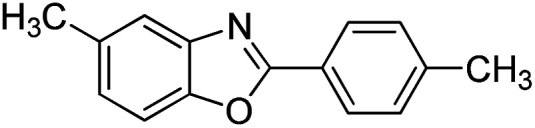	120 °C, 4 h	90
16	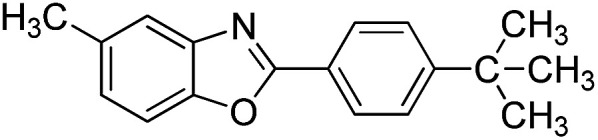	120 °C, 4 h	90
17	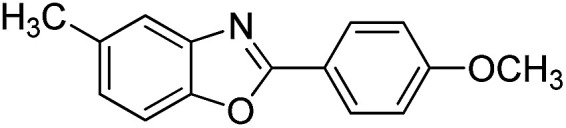	120 °C, 4 h	95
18	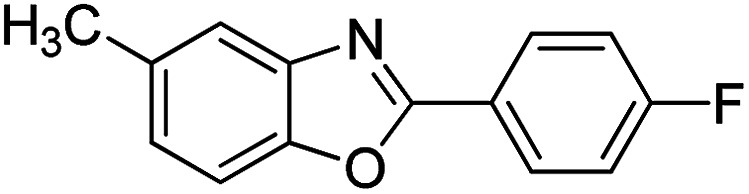	120 °C, 4.5 h	75
19	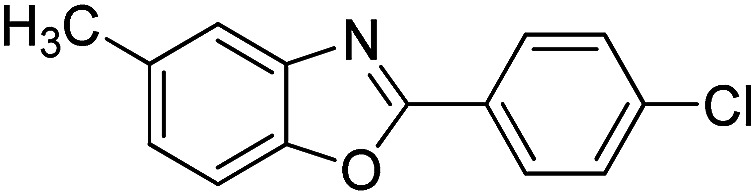	120 °C, 4.5 h	85
20	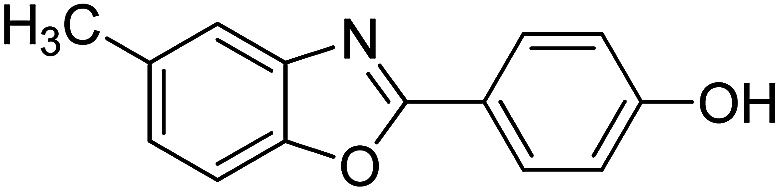	120 °C, 6.5 h	70
21	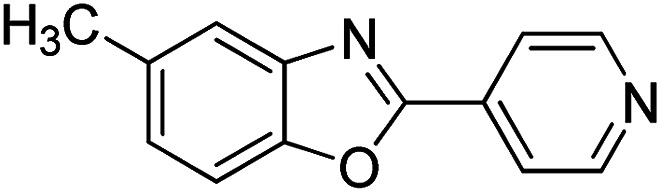	120 °C, 6 h	82
22	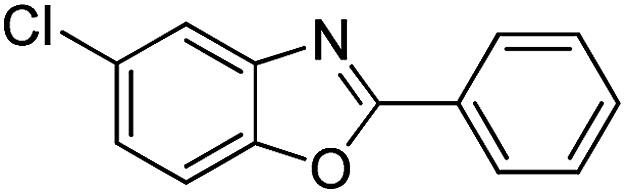	120 °C, 5 h	95
23	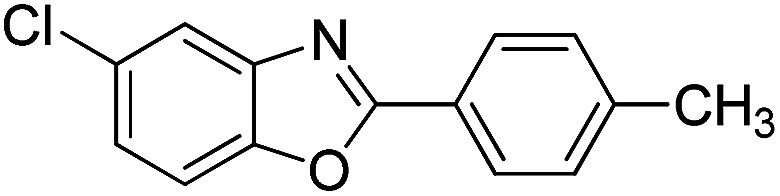	120 °C, 4 h	95
24	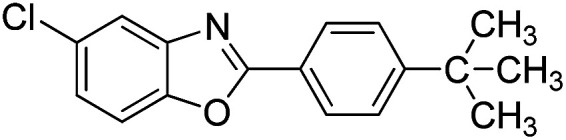	120 °C, 4 h	90
25	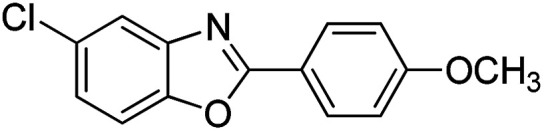	120 °C, 4 h	95
26	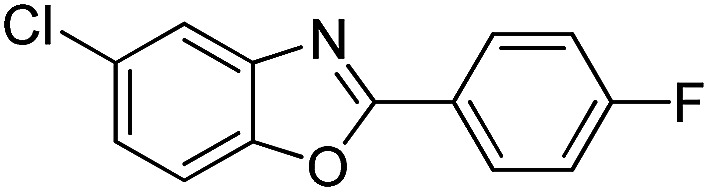	120 °C, 4 h	75
27	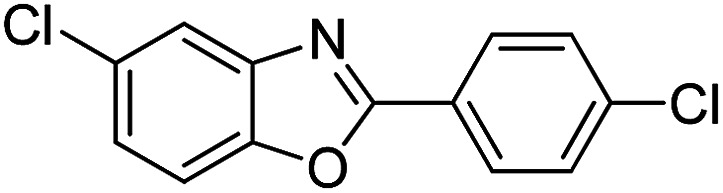	120 °C, 4 h	95
28	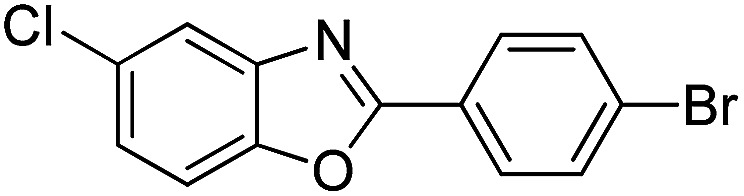	120 °C, 6 h	85
29	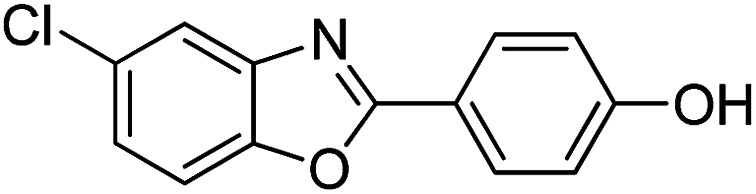	120 °C, 6.5 h	75
30	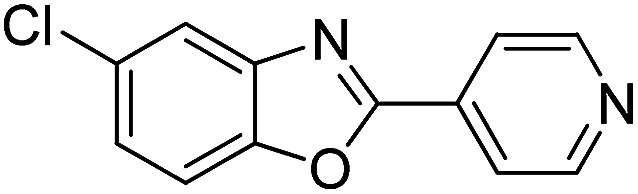	120 °C, 6 h	85
31	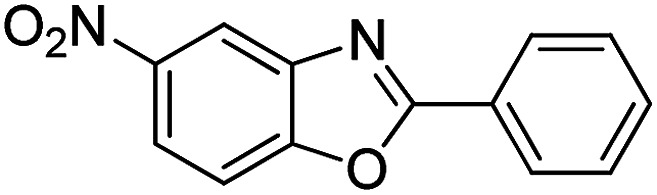	140 °C, 6 h	70
32	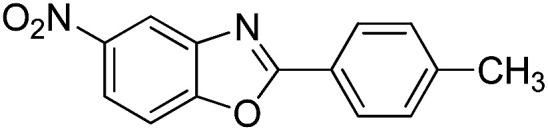	140 °C, 5 h	75
33	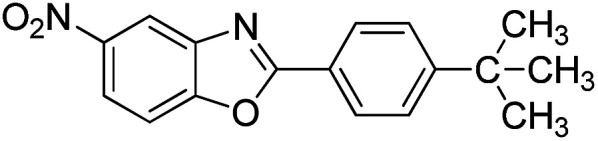	140 °C, 5 h	75
34	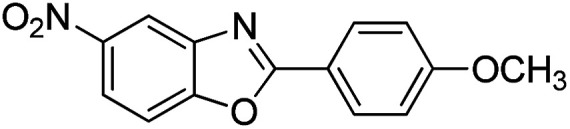	140 °C, 5 h	80
35	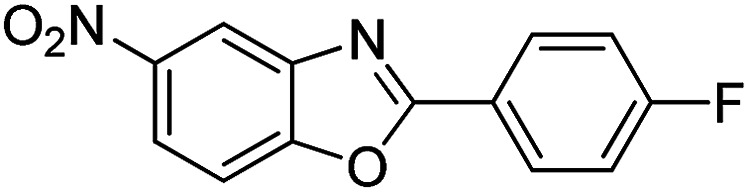	140 °C, 6 h	72
36	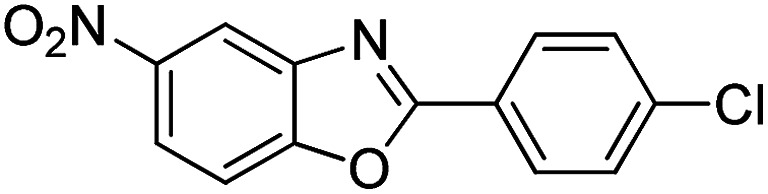	140 °C, 6 h	80
37	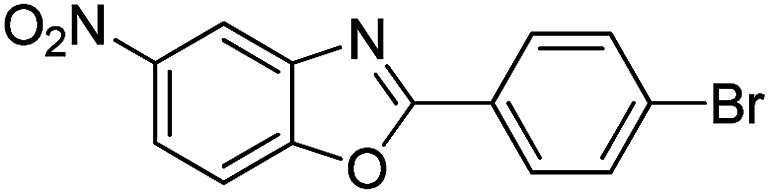	140 °C, 6 h	85

a Isolated yield.

b On 10 mmol or 20 mmol scale.

The versatility of benzothiazole and benzimidazole as substrates in the replacement of benzoxazole was also reported. The adducts resulted from the condensation of benzothiazole with various aromatic aldehydes were isolated in comparative yields with those derived from benzoxazole (Table 4, entries 1–6). Meanwhile, a failure in the formation of the desired product was noted for the case of benzimidazole (Table 4, entry 7), probably due to the existence of intermolecular hydrogen bonds between the NH of benzimidazole and DES.

**Table 4:** The arylation of benzothiazole and benzimidazole with aromatic aldehydes under current method

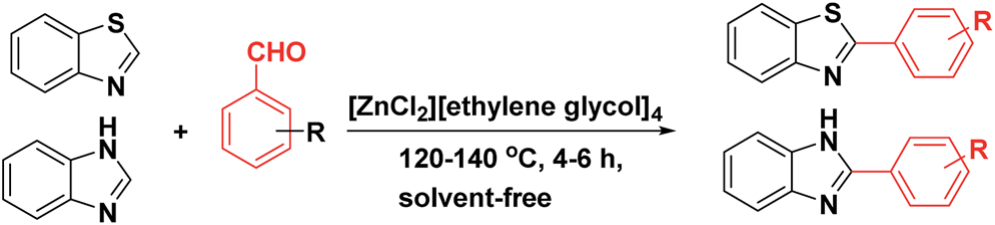			
Entry	Product	Condition	Yield^a^ (%)
1	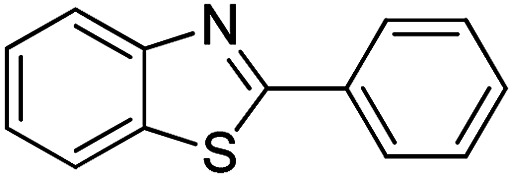	120 °C, 6 h	85
2	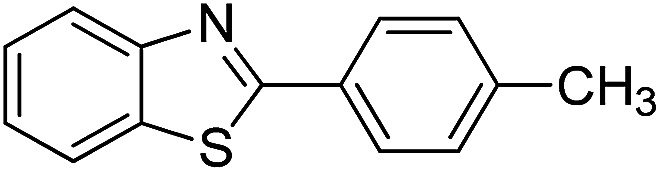	120 °C, 5 h	88
3	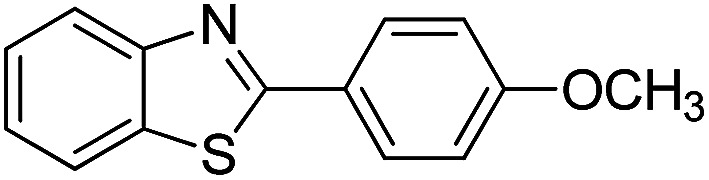	120 °C, 5 h	90
4	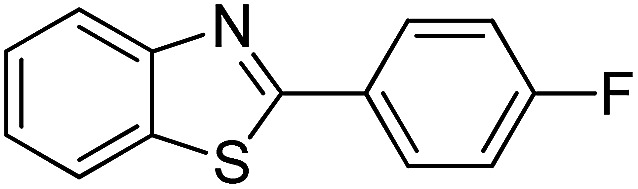	120 °C, 4 h	85
5	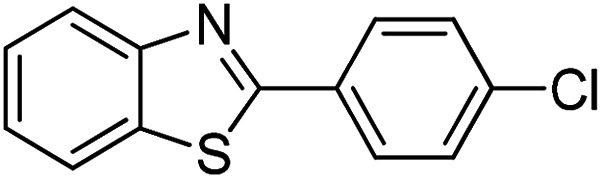	120 °C, 4 h	80
6	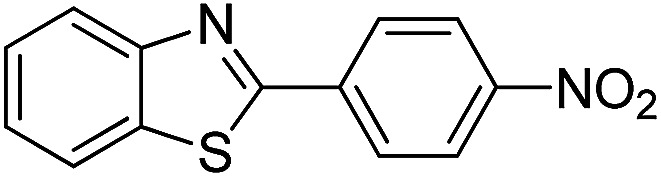	120 °C, 6.5 h	70
7	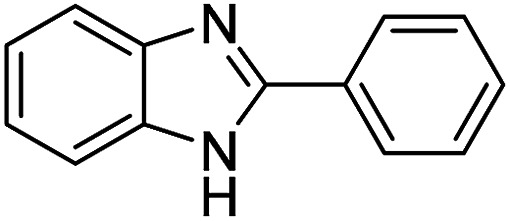	120–140 °C, 4–8 h	0

a Isolated yield.

A comparative study between the current method and previous ones was presented in Table 5. Deep eutectic solvent-catalyzed arylation of benzoxazole afforded the arylated benzoxazole products in excellent yields under a mild and simple condition without the demand for any additives as in preceding reports (Table 5, entry 6). Remarkably, no loss of catalytic activity in the recycling test of DES is the most prominent artifact of this protocol.

**Table 5:** The comparison of the current method with previous reports in the arylation of benzoxazole

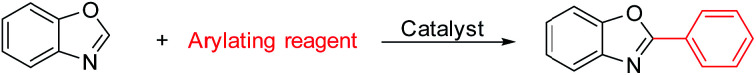				
Entry	Catalyst	Reagent	Condition	Yield (%)
1	FeSO_4_ (0.2 equiv.), H_2_O/diglyme/O_2_	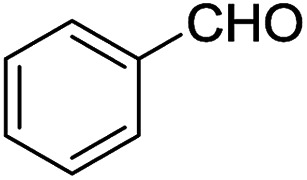	150 °C, 20 h	70 (ref. 31)
2	I_2_ (2 equiv.), PhCl, DMF	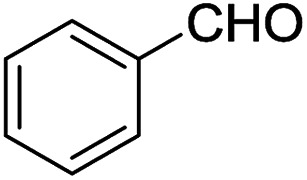	130 °C, 30 h	75 (ref. 30)
3	[Pd(π-allyl)Cl]_2_ (0.1 equiv.), PCy_3_, NaO*t*Bu (2 equiv.), DMF	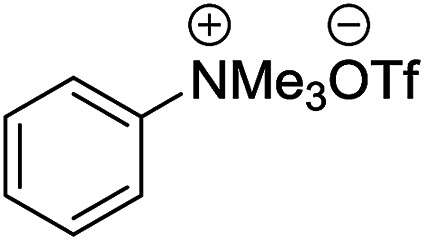	120 °C, 12 h	43 (ref. 7)
4	CuCN(PPh_3_)_2_ (10 mol%), PPh_3_, Cs_2_CO_3_, pivalonitrile	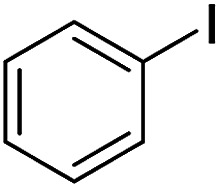	Reflux, 24 h	85 (ref. 28)
5	Ni(COD)_2_ (0.1 equiv.), dcype (0.2 equiv.), Cs_2_CO_3_ (1.5 equiv.), *p*-xylene	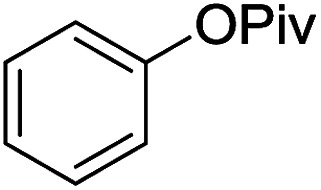	140 °C, 22 h	91 (ref. 29)
6	Current work: [ZnCl_2_][ethylene glycol]_4_ (5 mol%), solvent-free	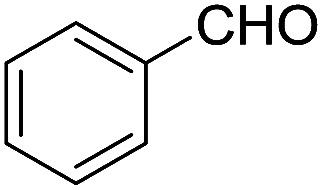	120 °C, 6 h	95

As asserted in previous literature through isotope-labeling mechanistic studies, the arylation of benzoxazoles with aromatic aldehydes underwent the ring-opening step assisted by Lewis acids such as I_2_ or FeSO_4_ to afford the key intermediate 2-aminophenol. Its nucleophilic addition to aldehydes followed by oxidative ring closure provided arylated benzoxazoles as final products.^30,31^ As an extra part in our research to check the conformability of the proposed mechanism for the same reaction catalyzed by [ZnCl_2_][ethylene glycol]_4_, we also carried out the acylation–cyclization of 2-aminophenol with benzaldehyde under the same optimized conditions which were previously applied for the arylation of benzoxazole by benzaldehyde. As the result, the same arylated benzoxazole product was obtained in a comparable yield of 85%. Additionally, in another control experiment whereby benzoxazole reacted with [ZnCl_2_][ethylene glycol]_4_ in the absence of benzaldehyde, 2-aminophenol was obtained in 62% yield. Thus, it is not doubtful that the arylation of benzoxazole studied herein must also undergo the ring-opening step prior to the condensation step with aldehydes. Although the mechanism is not clear now, the method possesses attractive merits including cheap and recyclable catalyst, non-toxicity, and wide scope of substrates.

**Fig. 4:**
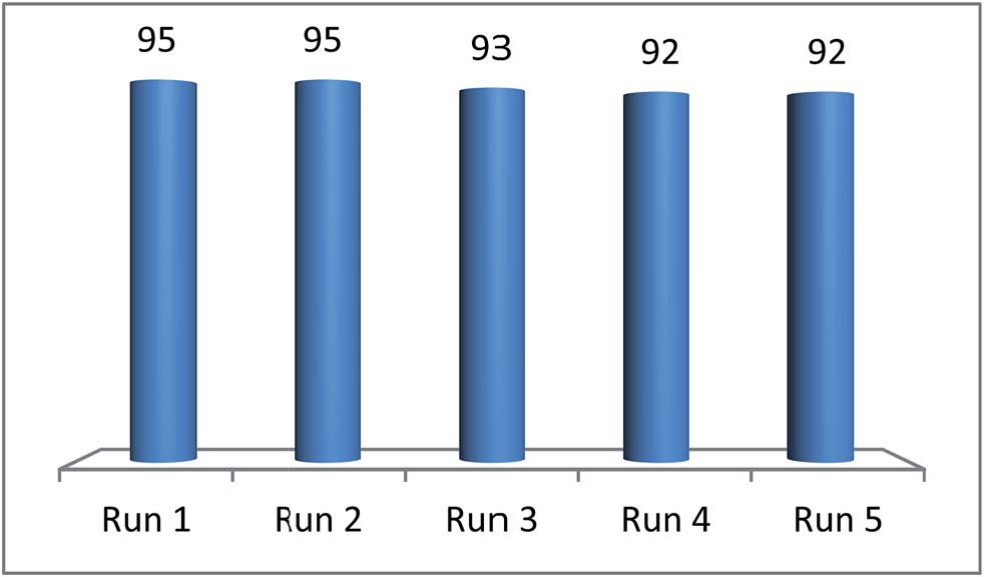
Reuse of [ZnCl_2_][ethylene glycol]_4_ catalyst in the arylation of benzoxazole with benzaldehyde.

**Fig. 5:**
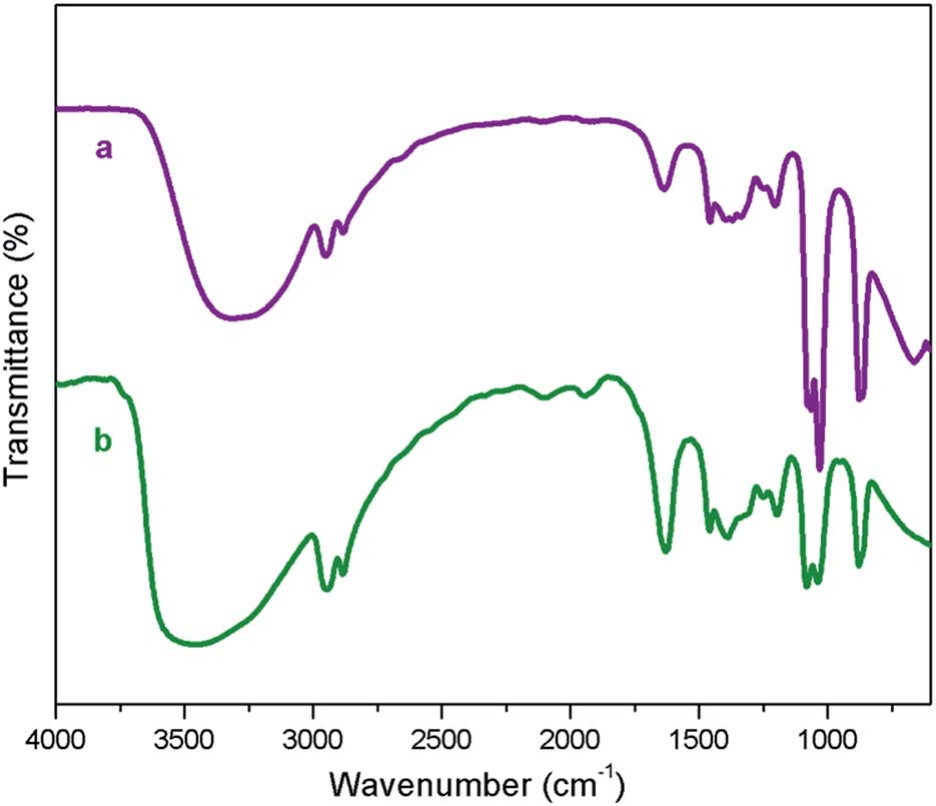
FT-IR of fresh [ZnCl_2_][ethylene glycol]_4_ (a) and [ZnCl_2_][ethylene glycol]_4_ after the fifth recovery (b).

The recyclability is an important feature for applying a catalyst in industrial processes. The recyclability of deep eutectic solvent of zinc chloride and ethylene glycol under study in this work was investigated in the model reaction. After completion of the reaction, the product was extracted with diethyl ether (10 × 5 mL), the catalyst was separated from the ethereal solution and dried under vacuum. The recovered catalyst was reused in the model reaction to the next run. As illustrated in Fig. 4, the efficiency of deep eutectic solvent was found to be constantly excellent even after five consecutive recycles. IR spectroscopy of fresh and recovered deep eutectic solvent indicated that no detectable structural degradation can be seen (Fig. 5). After each recycling test, a very small amount of DES leaching to diethyl ether phase during the work-up step was indirectly estimated by means of ICP-MS technique in which Zn content of about 0.08 ppm in the ethereal phase was determined. A slight decrease of catalytic activity was observed due to a little loss of deep eutectic solvent during the work-up process.

## Experimental

### General procedure for catalytic arylation of benzoxazole

Benzoxazole (119 mg, 1.0 mmol) was treated with benzaldehyde (53 mg, 0.5 mmol) in the presence of [ZnCl_2_][ethylene glycol]_4_ (5 mg, 0.01 mmol) at 120 °C for 6 h under solvent-free magnetic stirring. The completion of the reaction was checked by TLC and GC. The mixture was then diluted with diethyl ether (10 × 5 mL). The solvent was removed on a rotary evaporator. The crude product was purified by silica gel chromatography using acetone/petroleum ether (1/19) to afford the desired product (95% yield). The purity and identity of the product were confirmed by FT-IR, ^1^H NMR, ^13^C NMR, and MS. The recovered catalyst was activated by heating under reduced vacuum at 80 °C for 30 min and reused for next cycles.

## Conclusions

In conclusion, we have developed the first DES-mediated arylation of benzoxazoles with aromatic aldehydes at the C2 position under solvent-free condition. The most important highlight of the current method is the use of inexpensive, air- and water-stable, and recyclable deep eutectic solvent in the replacement of high-cost palladium catalysts. Interestingly, only a catalytic amount of deep eutectic solvent is requisite for the quantitative production of various 2-arylbenzoxazoles through a simplistic, environmentally benign, and low-cost synthetic procedure.

## Conflicts of interest

There are no conflicts to declare.

## Supplementary Material

RA-008-C8RA01094C-s001
