# Supplementary material for: Deep eutectic solvent-catalyzed arylation of benzoxazoles with aromatic aldehydes
Source: RSC Adv. 2018 Mar 20;8(20):11127–33. doi: 10.1039/c8ra01094c (PMC9078944; doi:10.1039/c8ra01094c)
Supplement: RA-008-C8RA01094C-s001 [file RA-008-C8RA01094C-s001.pdf]

Electronic Supplementary Material (ESI) for RSC Advances.
This journal is © The Royal Society of Chemistry 2018

Supporting Information

Deep eutectic solvent-catalyzed the arylation of benzoxazoles with aromatic aldehydes

Phuong Hoang Tran,* Anh-Hung Thi Hang

Department of Organic Chemistry, Faculty of Chemistry, University of Science, Viet Nam National University, Ho Chi Minh City 721337, Viet Nam

*To whom correspondence should be addressed: thphuong@hcmus.edu.vn (Phuong Hoang Tran)

Table of Contents

**Section S1**	Materials and Analytical Techniques	S3
**Section S2**	Procedure for the synthesis of deep eutectic solvents	S4
**Section S3**	Characterization of the products	S5-S17
**Section S4**	References	S18
**Section S5**	NMR spectra	S19-S61

Section S1. Materials and Analytical Techniques

Materials

Zinc chloride (reagent great ≥ 98%) and ethylene glycol (99.8%) was obtained from Sigma-Aldrich. Benzoxazole (98%) were purchased from Acros Organics, 5-methylbenzoxazole (97%) were obtained from Sigma-Aldrich Chemical Company, 5-chlorobenzoxazole (95%) were obtained from Sigma-Aldrich Chemical Company, 5-nitrobenzoxazole (97%) were purchased from Acros Organics. Benzaldehyde (99%), 4-methylbenzaldehyde (97%), 4-methoxybenzaldehyde (98%), 4-*tert*-butylbenzaldehyde (97%), 4-fluorobenzaldehyde (98%), 4-chlorobenzaldehyde (97%), 4-bromobenzaldehyde (99%), 3-fluorobenzaldehyde (97%), 3-bromobenzaldehyde (97%), 3-chlorobenzaldehyde (97%), 2-fluorobenzaldehyde (97%), 2-bromobenzaldehyde (98%), were obtained from Sigma-Aldrich Chemical Company.

Silica gel 230 – 400 mesh, for flash chromatography) was obtained from HiMedia Laboratories Pvt. Ltd. (India). TLC (silica gel 60 F_{254}) was obtained from Merk. Ethyl acetate (purity ≥ 99.5%), hexane (≥ 95%), chloroform (purity ≥ 99%) were obtained from Xilong Chemical Co., Ltd (China). Chloroform-d, 99.8 Atom %D, stab. with Ag was obtained from Armar (Switzerland).

All starting materials, reagent and solvents were used without further purification

Analytical Techniques

Gas chromatography-mass spectrometry measurements were carried out on an Agilent GC System 7890 equipped with a mass selective detector (Agilent 5973N) and a capillary DB-5MS column (30 m × 250 μm × 0.25 μm). Analytical thin-layer chromatography (TLC) was performed on F-254 silica gel coated aluminum plates from Merck. Silica gel column chromatography was carried out with silica gel (60, 230-400 mesh) from Merck. ^{1}H and ^{13}C nuclear magnetic resonance (NMR) spectra were recorded on a Bruker Advance II 500 MHz NMR spectrometer. Fourier Transform infrared (FT-IR) spectra were recorded from KBr pellets using a Bruker Vertex 70 system. Thermal gravimetric analysis (TGA) was performed on a TA Q500 thermal analysis system with the sample held in a platinum pan in a continuous airflow. Raman spectra were recorded on a Horiba Xplora One using a 532 nm argon ion laser. ICP-MS was recorded on a PerkinElmer 350X.

Section S2 Procedure for the Synthesis of deep eutectic solvents

$[ZnCl_2]$[ethylene glycol]$_4$: A mixture of zinc chloride (5 mmol, 0.68 g) and ethylene glycol (20 mmol, 1.240 g) was heated to 120 ^{o}C until a clear colorless liquid was obtained.

[ChCl]$[ZnCl_2]_3$: A mixture of choline chloride (5 mmol, 0.695 g) and zinc chloride

(15 mmol, 2.04 g) was heated to 100 ^{o}C until a clear colorless liquid was obtained.

$[ZnCl_2]_4$[urea]: A mixture of zinc chloride (20 mmol, 0.272 g) and urea

(5 mmol, 0.3 g) was heated to 100 ^{o}C until a clear colorless liquid was obtained.

$[ZnCl_2]$[glycerol]$_4$: A mixture of zinc chloride (5 mmol, 0.68 g) and glycerol

(20 mmol, 1,840 g) was heated to 100 ^{o}C until a clear colorless liquid was obtained.

$[ZnCl_2]$[acetamide]$_4$: A mixture of zinc chloride (5 mmol, 0.68 g) and acetamide

(20 mmol, 1.180 g) was heated to 100 ^{o}C until a clear colorless liquid was obtained.

Section S3. Characterization of the products

2-Phenylbenzoxazole[1-3]

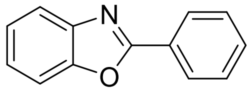

Analytical TLC on silica gel, 1/19 acetone/petroleum ether.
White solid, Yield 95%, mp = 102-103 °C.
FT-IR (KBr, 4000 – 400 cm^{-1}): 3059, 2925, 2854, 1775, 1615, 1551, 1475, 1448, 1285, 1240.
^{1}H NMR (500 MHz, $CDCl_3$): δ 8.29 – 8.25 (m, 2H), 7.80 – 7.77 (m, 1H), 7.60 – 7.57 (m, 1H), 7.55 – 7.50 (m, 3H), 7.37 – 7.34 (m, 2H).
^{13}C NMR (125 MHz, $CDCl_3$) δ 163.2, 150.9, 142.2, 131.7, 129.0, 127.8, 127.3, 125.3, 124.7, 120.1, 110.7.
GC-MS (EI, 70 eV) *m/z*: 195 ([M]$^+$)

2-(4-Methylphenyl)benzoxazole[1,2]

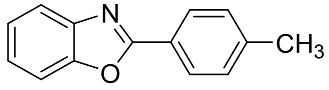

Analytical TLC on silica gel, 1/19 acetone/petroleum ether.
White solid, Yield 93%, mp = 113-114 °C.
FT-IR (KBr, 4000 – 400 cm^{-1}): 3056, 2920, 2854, 1728, 1620, 1554, 1499, 1450, 1242.
^{1}H NMR (500 MHz, $CDCl_3$) δ 8.16 – 8.14 (m, 2H), 7.77 – 7.74 (m, 1H), 7.58 – 7.55 (m, 1H), 7.36 – 7.32 (m, 4H), 2.44 (s, 3H).
^{13}C NMR (125 MHz, $CDCl_3$) δ 163.5, 150.8, 142.3, 142.2, 129.8, 127.8, 125.1, 124.7, 124.5, 120.0, 110.6, 21.8.
GC-MS (EI, 70 eV) *m/z*: 209 ([M]$^+$)

2-(4-*Tert*-butylphenyl)benzoxazole[2]

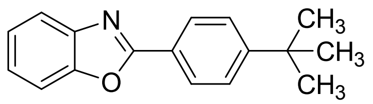

Analytical TLC on silica gel, 1/19 acetone/petroleum ether.
White solid, Yield 94%, mp = 107-108 °C.
FT-IR (KBr, 4000 – 400 cm^{-1}): 3059, 2927, 1728, 1547, 1452, 1429, 1287, 1239.
^{1}H NMR (500 MHz, $CDCl_3$) δ 8.20 – 8.18 (m, 2H), 7.75 – 7.78 (m, 1H), 7.59 – 7.57 (m, 1H), 7.56 – 7.54 (m, 2H), 7.36 – 7.32 (m, 2H), 1.38 (s, 9H).
^{13}C NMR (125 MHz, $CDCl_3$) δ 163.4, 155.4, 150.8, 142.1, 127.7, 126.1, 125.1, 124.7, 124.4, 120.0, 110.7, 35.2, 31.3.
GC-MS (EI, 70 eV) *m/z*: 251 ([M]$^+$)

2-(4-Methoxyphenyl)benzoxazole[2]

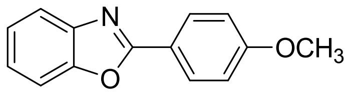

Analytical TLC on silica gel, 1/19 acetone/petroleum ether.
White solid, Yield 95%, mp = 98-100 °C.
FT-IR (KBr, 4000 – 400 cm^{-1}): 3050, 2924, 2849, 1615, 1501, 1450, 1420, 1244.
^{1}H NMR (500 MHz, $CDCl_3$) δ 8.21 – 8.18 (m, 2H), 7.75 – 7.73 (m, 1H), 7.56 – 7.54 (m, 1H), 7.35 – 7.30 (m, 2H), 7.04 – 7.01 (m, 2H),

3.89 (s, 3H).
^{13}C NMR (125 MHz, $CDCl_3$) *δ* 163.3, 162.6, 150.8, 142.1, 129.7, 124.8, 124.7, 119.7, 119.7, 114.6, 110.6, 55.6.
GC-MS (EI, 70 eV) *m/z*: 225 ($[M]^+$)

2-(4-Nitrophenyl)benzoxazole[3]

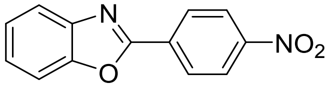

Analytical TLC on silica gel, 1/19 acetone/petroleum ether.
Yellow solid, Yield 75%, mp = 250 - 252 ^{o}C.
FT-IR (KBr, 4000 – 400 cm^{-1}): 2925, 2854, 1678, 1610, 1534, 1449, 1237.
^{1}H NMR (500 MHz, $CDCl_3$) *δ* 8.15 (dd, *J* = 8.0, 1.0 Hz, 1H), 7.89 (dd, *J* = 8.0, 1.0 Hz, 1H), 7.83 – 7.80 (m, 1H), 7.74 (td, *J* = 8.0, 1.0 Hz, 1H), 7.69 (td, *J* = 8.0, 1.0 Hz, 1H), 7.59 – 7.57 (m, 1H), 7.42 – 7.37 (m, 2H).
^{13}C NMR (125 MHz, $CDCl_3$) *δ* 158.9, 151.2, 141.7, 132.4, 132.0, 131.6, 126.2, 125.1, 124.3, 120.9, 111.1.
GC-MS (EI, 70 eV) *m/z*: 240 ($[M]^+$)

2-(4-Fluorophenyl)benzoxazole[2]

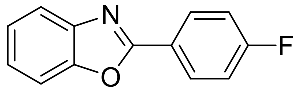

Analytical TLC on silica gel, 1/19 acetone/petroleum ether.
White solid, Yield 80%, mp = 99 - 99.5 ^{o}C.
FT-IR (KBr, 4000 – 400 cm^{-1}): 3061, 2925, 1619, 1584, 1582, 1542, 1473, 1448, 1247, 1225.
^{1}H NMR (500 MHz, $CDCl_3$) *δ* 8.27 – 8.23 (m, 2H), 7.78 – 7.74 (m, 1H), 7.58 – 7.55 (m, 1H), 7.37 – 7.33 (m, 2H), 7.23 – 7.18 (m, 2H).
^{13}C NMR (125 MHz, $CDCl_3$) *δ* 165.0 (d, *J* = 251 Hz), 162.3, 150.9, 142.2, 130.0 (d, *J*=8.8 Hz), 130.0 (d, *J* = 255.1 Hz) 125.0 (d, *J* = 59.5 Hz), 123.7 (d, *J*=3.3 Hz), 120.1, 116.3 (d, *J*=22 Hz), 110.7.
GC-MS (EI, 70 eV) *m/z*: 213 ($[M]^+$)

2-(4-Chlorophenyl)benzoxazole[2]

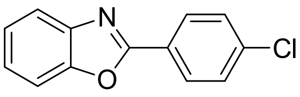

Analytical TLC on silica gel, 1/19 acetone/petroleum ether.
White solid, Yield 92%, mp = 146 - 147.5 ^{o}C.
FT-IR (KBr, 4000 – 400 cm^{-1}): 3061, 2925, 1610, 1584, 1582, 1542, 1473, 1448, 1247, 1225.
^{1}H NMR (500 MHz, acetone-d_6) δ 8.26 – 8.23 (m, 2H), 8.27 – 8.22 (m, 2H), 7.77 (m, 1H), 7.71–7.69 (m, 1H), 7.65 – 7.63 (m, 2H), 7.46 – 7.39 (m, 2H).
^{13}C NMR (125 MHz, acetone-d_6) δ 161.73, 150.81, 142.08, 137.23, 129.34, 128.99, 125.93, 125.59, 124.85, 120.02, 110.70.
GC-MS (EI, 70 eV) *m/z*: 229 ($[M]^+$)

2-(3-Fluorophenyl)benzoxazole[2]

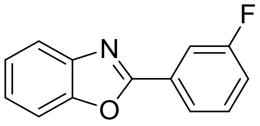

Analytical TLC on silica gel, 1/19 acetone/petroleum ether.
White solid, Yield 70%, mp = 99-100 °C.
FT-IR (KBr, 4000 – 400 cm^{-1}): 3061, 2925, 1650, 1584, 1582, 1542, 1473, 1448, 1247, 1225.
^{1}H NMR (500 MHz, $CDCl_3$): δ 8.05 – 8.04(m, 1H), 7.96 – 7.94 (m, 1H), 7.79 – 7.77 (m, 1H), 7.59-7.58 (m, 1H), 7.51-7.47 (m, 1H), 7.38 – 7.36 (m, 2H), 7.25 – 7.21 (m, 1H).
^{13}C NMR (125 MHz, $CDCl_3$) δ 163.0 (d, J = 245.5 Hz), 150.9, 142.1, 130.8 (d, J = 8.1 Hz), 129.3 (d, J = 8.5 Hz), 125.6, 124.9, 123.5 (d, J = 3 Hz), 120.4, 118.7, 118.6 (d, J = 21.3 Hz),114.7 (d, J= 23.9 Hz), 110.8.
GC-MS (EI, 70 eV) *m/z*: 213 ($[M]^+$)

2-(3-Bromophenyl)benzoxazole[3]

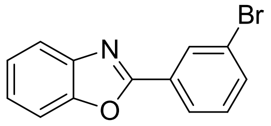

Analytical TLC on silica gel, 1/19 acetone/petroleum ether.
White solid, Yield 90%, mp = 129-131 °C.
FT-IR (KBr, 4000 – 400 cm^{-1}): 3423, 3055, 2926, 1613, 1570, 1545, 1451, 1426, 1287, 1240.
^{1}H NMR: (500 MHz, $CDCl_3$): δ 8.42 (t, J = 1.5 Hz, 1H), 8.20 – 8.18 (m, 1H), 7.79 – 7.77(m, 1H), 7.67 (m, 1H), 7.66 – 7.65 (m, 1H), 7.42 – 7.37 (m, 3H).
^{13}C NMR (125 MHz, $CDCl_3$)**:** δ 161.7, 151.0, 142.0, 134.6, 130.7, 130.6, 129.2, 126.3, 125.7, 125.0, 123.2, 120.4, 110.9.
GC-MS (EI, 70 eV) *m/z*: 273 ($[M]^+$)

2-(2-Fluorophenyl)benzoxazole[4]

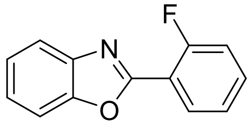

Analytical TLC on silica gel, 1/19 acetone/petroleum ether.
White solid, Yield 72%, mp = 93-95 °C.
FT-IR (KBr, 4000 – 400 cm^{-1}): 3061, 2925, 1719, 1584, 1582, 1542, 1473, 1448, 1247, 1225.
^{1}H NMR (500 MHz, $CDCl_3$) δ 8.24 (td, J = 7.5, 2.0 Hz, 1H), 7.85 – 7.81(m, 1H), 7.63 – 7.59 (m, 1H), 7.54 – 7.49 (m, 1H), 7.41 – 7.36 (m, 2H), 7.32 – 7.25 (m, 2H).
^{13}C NMR (125 MHz, $CDCl_3$)**:** δ 161.0 (d, J = 257.3 Hz), 159.6 (d, J = 5.3 Hz), 150.7, 141.9, 133.2 (d, J =8.6), 130.7, 125.6, 124.8, 124.6 (d, J = 3.8 Hz),120.5, 117.2 (d, J = 21,4), 115.7 (d, J = 10.4 Hz), 110.8.
GC-MS (EI, 70 eV) *m/z*: 213 ($[M]^+$)

2-(2-Chlorophenyl)benzoxazole[5]

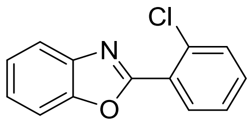

Analytical TLC on silica gel, 1/19 acetone/petroleum ether.
White solid, Yield 90%, mp = 100-102 °C.
FT-IR (KBr, 4000 – 400 cm^{-1}): 2925, 1608, 1584, 1569, 1533, 1470, 1452, 1343, 1237, 1185.

^{1}H NMR (500 MHz, $CDCl_3$)**:** δ 8.16 – 8.14 (m, 1H), 7.87 – 7.84 (m, 1H), 7.64 – 7.61 (m, 1H), 7.58 – 7.56 (m, 1H), 7.47 – 7.37 (m, 4H).
^{13}C NMR (125 MHz, $CDCl_3$)**:** δ 161.1, 150.8, 141.8, 133.7, 132.1, 132.0, 131.5, 127.1, 126.5, 125.7, 124.8, 120.7, 110.9.
GC-MS (EI, 70 eV) *m/z*: 229 ($[M]^+$)

2-(2-Hydroxyphenyl)benzoxazole[3]

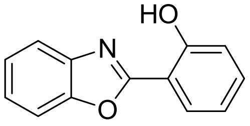

Analytical TLC on silica gel, 1/19 acetone/petroleum ether.
White solid, Yield 93%, mp = 120-122 °C.
FT-IR (KBr, 4000 – 400 cm^{-1}): 2921, 2851, 1630, 1587, 1543, 1487, 1452, 1244, 1155.
^{1}H NMR (500 MHz, $CDCl_3$)**:** δ 8.04 (dd, J = 8.0, 1.5 Hz, 1H), 7.74 (m, 1H), 7.63 – 7.60 (m, 1H), 7.47 – 7.42 (m, 1H), 7.41 – 7.37 (m, 2H), 7.13 (d, J = 8.5 Hz, 1H), 7.03 – 7.00 (m, 1H).
^{13}C NMR (125 MHz, $CDCl_3$)**:** δ 133.72, 127.29, 125.54, 125.17, 119.72, 119.43, 117.59, 110.82.
GC-MS (EI, 70 eV) *m/z*: 213 ($[M]^+$)

2-(Pyridine-4-yl)benzoxazole[6]

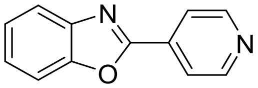

Analytical TLC on silica gel, 1/19 acetone/petroleum ether.
White solid, Yield 80%, mp = 102-104°C.
FT-IR (KBr, 4000 – 400 cm^{-1}): 3460, 2946, 1628, 878.
^{1}H NMR (500 MHz, $CDCl_3$): δ 8.82 (d, J = 5.5 Hz, 2H), 8.09 (d, J = 6.0 Hz, 2H), 7.84 – 7.80 (m, 1H), 7.63 (m, 1H), 7.42 (m, 2H).
^{13}C NMR (125 MHz, $CDCl_3$): δ 161.0, 151.1, 150.9, 141.9, 134.6, 126.5, 125.3, 121.2, 120.9, 111.1.
GC-MS (EI, 70 eV) *m/z*: 196 ($[M]^+$)

5-Methyl-2-phenylbenzoxazole[2]

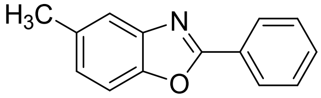

Analytical TLC on silica gel, 1/19 acetone/petroleum ether.
White solid, Yield 94%, mp = 112-115 °C.
FT-IR (KBr, 4000 – 400 cm^{-1}): 3422, 2922, 2869, 1647, 1550, 1474, 1334, 1261.
^{1}H NMR (500 MHz, acetone-d_6**)** δ 8.25 – 8.23 (m, 2H), 7.62 – 7.59 (m, 3H), 7.57 – 7.56 (m, 2H), 7.24 (d, J = 8.5, 1H), 2.47 (s, 3H).
^{13}C NMR (125 MHz, $CDCl_3$): δ 163.3, 149.2, 142.5, 134.5, 131.5, 129.0, 127.7, 127.5, 126.4, 120.1, 110.1, 21.6.
GC-MS (EI, 70 eV) *m/z*: 209 ($[M]^+$)

5-Methyl-2-(*p*-tolyl)benzoxazole[7]

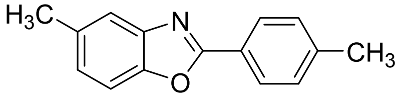

Analytical TLC on silica gel, 1/19 acetone/petroleum ether.
White solid, Yield 90%, mp = 134-135 °C.
FT-IR (KBr, 4000 – 400 cm^{-1}): 3304, 2921, 2856, 1615, 1501, 1450,

1333, 1262.
^{1}H NMR (500 MHz, acetone-d_6) δ 8.11 (d, J = 8.0 Hz, 2H), 7.53 – 7.51 (m, 2H), 7.39 (d, J = 8.0 Hz, 2H), 7.22 – 7.19 (m, 1H), 2.46 (s, 3H), 2.43 (s, 3H).
^{13}C NMR (125 MHz, acetone-d_6) δ 163.2, 149.2, 142.7, 142.3, 134.5, 129.9, 127.5, 126.3, 124.8, 119.9, 110.1, 20.9, 20.8.
GC-MS (EI, 70 eV) *m/z*: 223 ([M]$^+$)

5-Methyl-2-(4-*tert*-butylphenyl)benzoxazole[4]

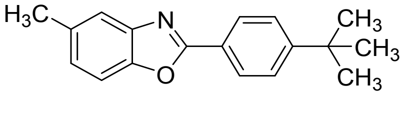

Analytical TLC on silica gel, 1/19 acetone/petroleum ether.
White solid, Yield 90%, mp = 137-140 °C.
FT-IR (KBr, 4000 – 400 cm^{-1}): 2956, 2902, 2865, 1611, 1572, 1552, 1493, 1457, 1262, 1230.
^{1}H NMR (500 MHz, acetone-d_6) δ 8.56 (d, J = 2.0 Hz, 1H), 8.34 (dd, J = 9.0, 2.0 Hz, 1H), 8.20 (d, J = 8.5 Hz, 2H), 7.92 (d, J = 9.0 Hz, 1H), 7.69 (d, J = 9.0 Hz, 2H), 2.81 (s, 3H), 1.39 (s, 9H).
^{13}C NMR (125 MHz, acetone-d_6) δ 162.9, 155.0, 149.0, 142.5, 134.3, 127.2, 126.1, 126.0, 124.6, 119.7, 109.9, 34.7, 30.5, 20.5.
GC-MS (EI, 70 eV) *m/z*: 265 ([M]$^+$)

5-Methyl-2-(4-methoxyphenyl)benzoxazole[7]

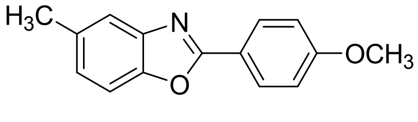

Analytical TLC on silica gel, 1/19 acetone/petroleum ether.
White solid, Yield 95%, mp = 112 °C.
FT-IR (KBr, 4000 – 400 cm^{-1}): 3050, 2924, 2854, 1608, 1558, 1450, 1420, 1254, 1018.
^{1}H NMR (500 MHz, DMSO-d_6) δ 8.12 (d, J = 9.0 Hz, 2H), 7.61 (d, J = 8.0 Hz, 1H), 7.55 (s, 1H), 7.20 (d, J = 8.5 Hz, 1H), 7.15 (d, J = 9.0 Hz, 2H), 3.87 (s, 3H), 2.44 (s, 3H).
^{13}C NMR (125 MHz, DMSO-d_6) δ 162.5, 148.7, 142.1, 134.7, 129.5, 126.5, 119.6, 119.5, 119.2, 115.2, 110.6, 55.9, 21.3.
GC-MS (EI, 70 eV) *m/z*: 239 ([M]$^+$)

5-Methyl-2-(4-chlorophenyl)benzoxazole[4]

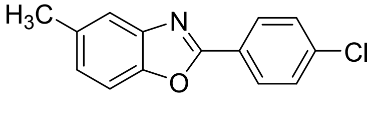

Analytical TLC on silica gel, 1/19 acetone/petroleum ether.
White solid, Yield 85%, mp = 125 °C.
FT-IR (KBr, 4000 – 400 cm^{-1}): 3050, 2957, 2849, 1615, 1501, 1450, 1420, 1287.
^{1}H NMR (500 MHz, $CDCl_3$): δ 8.18 (d, J = 9.0 Hz, 2H), 7.55 (s, 1H), 7.50 (d, J = 8.5 Hz, 2H), 7.45 (d, J = 8.0 Hz, 1H), 7.17 (d, J = 9.0 Hz, 1H), 2.49 (s, 3H).
^{13}C NMR (125 MHz, acetone-d_6): δ 162.3, 149.2, 142.4, 137.7, 134.7, 129.3, 128.9, 126.6, 126.0, 120.1, 110.1, 21.6.
GC-MS (EI, 70 eV) *m/z*: 243 ([M]$^+$)

5-Methyl-2-(4-fluorophenyl)benzoxazole[4]

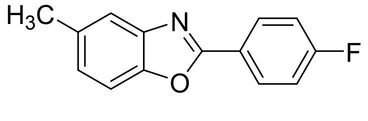

Analytical TLC on silica gel, 1/19 acetone/petroleum ether.

White solid, Yield 75%, mp = 170 °C.
FT-IR (KBr, 4000 – 400 cm^{-1}): 3050, 2957, 2849, 1615, 1501, 1450, 1420, 1287.
^{1}H NMR (500 MHz, $CDCl_3$) δ 8.27 – 8.17 (m, 2H), 7.53 (s, 1H), 7.42 (d, J = 8.5 Hz, 1H), 7.21 – 7.12 (m, 3H), 2.47 (s, 3H).
^{13}C NMR (125 MHz, $CDCl_3$): δ 164.9 (d, J = 250.9 Hz), 162.4, 149.2, 142.4, 134.6, 129.9 (d, J = 8.8 Hz), 126.4, 123.8 (d, J = 3 Hz), 120.0, 116.2 (d, J = 22.1 Hz), 110.0, 21.6.
GC-MS (EI, 70 eV) *m/z*: 227 ($[M]^+$)

5-Methyl-2-(4-hydroxyphenyl)benzoxazole[8]

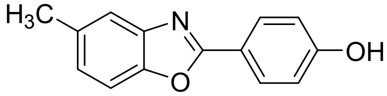

Analytical TLC on silica gel, 1/19 acetone/petroleum ether.
White solid, Yield 70%, mp = 220-221 °C.
FT-IR (KBr, 4000 – 400 cm^{-1}): 3050, 2957, 2849, 1615, 1501, 1450, 1420, 1287.
^{1}H NMR (500 MHz, acetone-d_6): δ 9.23 (s, 1H), 8.10 (d, J = 8.5 Hz, 2H), 7.50 (d, J = 8.5 Hz, 2H), 7.18 (d, J = 8.5 Hz, 1H), 7.03 (d, J = 8.5 Hz, 2H), 2.45 (s, 3H).
^{13}C NMR (125 MHz, acetone-d_6): δ 161.9, 154.5, 149.0, 143.6, 135.0, 132.8, 130.2, 126.5, 120.3, 116.8, 110.6, 21.4.
GC-MS (EI, 70 eV) *m/z*: 225 ($[M]^+$)

5-Methyl-2-(pyridin-4-yl)benzoxazole[9]

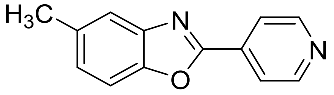

Analytical TLC on silica gel, 1/19 acetone/petroleum ether.
White solid, Yield 82%, mp = 129 - 130 °C.
FT-IR (KBr, 4000 – 400 cm^{-1}): 3406, 2921, 1612, 1537, 1414, 1344, 1068, 808.
^{1}H NMR (500 MHz, $CDCl_3$): δ 8.80 (d, J = 4.5 Hz, 2H), 8.07 (d, J = 5.0 Hz, 2H), 7.59 (s, 1H), 7.48 (d, J = 8.5 Hz, 1H), 7.23 (d, J = 8.0 Hz, 1H), 2.50 (s, 3H).
^{13}C NMR (125 MHz, $CDCl_3$): δ 160.8, 150.7, 149.3, 142.1, 135.2, 134.8, 127.7, 121.1, 120.6, 110.4, 21.6.
GC-MS (EI, 70 eV) *m/z*: 210 ($[M]^+$)

5-Chloro-2-phenylbenzoxazole[5]

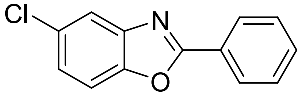

Analytical TLC on silica gel, 1/19 acetone/petroleum ether.
White solid, Yield 95%, mp = 102-104 °C.
FT-IR (KBr, 4000 – 400 cm^{-1}): 3051, 2920, 1650, 1584, 1582, 1542, 1473, 1438, 1267, 1221.
^{1}H NMR (500 MHz, acetone-d_6) δ 8.23 (d, J = 7.5 Hz, 2H), 7.77 (d, J = 1.5 Hz, 1H), 7.70 (d, J = 7.5 Hz, 1H), 7.59 – 7.64 (m, 3H), 7.42 (dd, J = 9.0, 1.5 Hz, 1H).
^{13}C NMR (125 MHz, acetone-d_6) δ 165.3, 150.6, 144.5, 133.1, 130.7, 130.2, 128.6, 127.7, 126.4, 120.7, 112.9.
GC-MS (EI, 70 eV) *m/z*: 229 ($[M]^+$)

5-Chloro-2-(*p*-tolyl)benzoxazole[10]

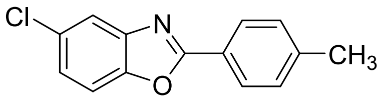

Analytical TLC on silica gel, 1/19 acetone/petroleum ether.
White solid, Yield 95%, mp = 138-140 °C.
FT-IR (KBr, 4000 – 400 cm^{-1}): 3061, 2925, 1610, 1551, 1479, 1331, 1258.
^{1}H NMR (500 MHz, acetone-d_6) δ 8.13 (d, J = 8.0 Hz, 2H), 7.75 (d, J = 2.0 Hz, 1H), 7.70 (d, J = 9.0 Hz, 1H), 7.40 – 7.33 (m, 4H), 2.44 (s, 3H).
^{13}C NMR (125 MHz, acetone-d_6) δ 165.6, 150.5, 144.6, 143.8, 130.8, 130.6, 128.6, 126.2, 125.0, 120.5, 112.8, 21.7.
GC-MS (EI, 70 eV) *m/z*: 243 ($[M]^+$)

5-Chloro-2-(4-*tert*-butylphenyl)benzoxazole[11]

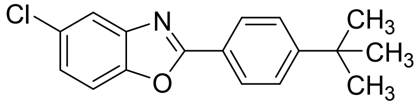

Analytical TLC on silica gel, 1/19 acetone/petroleum ether.
White solid, Yield 90%, mp = 140-142 °C.
FT-IR (KBr, 4000 – 400 cm^{-1}): 3061, 2919, 2851, 1596, 1582, 1552, 1478, 1459, 1260, 1200.
^{1}H NMR (500 MHz, acetone-d_6) δ 8.17 (d, J = 8.5 Hz, 2H), 7.76 (s, 1H), 7.70 (d, J = 8.5 Hz, 1H), 7.66 (d, J = 8.5 Hz, 2H), 7.41 (dd, J = 8.5, 2.0 Hz, 1H), 1.38 (s, 9H).
^{13}C NMR (125 MHz, acetone-d_6) δ 165.5, 156.7, 150.6, 144.6, 130.6, 128.6, 127.2, 126.2, 125.0, 120.5, 112.8, 35.9, 31.5.
GC-MS (EI, 70 eV) *m/z*: 285 ($[M]^+$)

5-Chloro-2-(4-methoxyphenyl)benzoxazole[10]

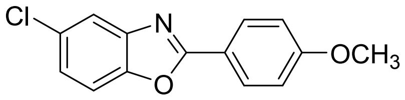

Analytical TLC on silica gel, 1/19 acetone/petroleum ether.
White solid, Yield 95%, mp = 148-150 °C.
FT-IR (KBr, 4000 – 400 cm^{-1}): 3742, 2962, 1609, 1554, 1496, 1451, 1417, 1251, 1197.
^{1}H NMR (500 MHz, acetone-d_6) δ 8.19 – 8.17 (m, 2H), 7.72 (d, J = 2.0 Hz, 1H), 7.67 (d, J = 8.5 Hz, 1H), 7.38 (dd, J = 8.5, 2.0 Hz, 1H), 7.16 – 7.14 (m, 2H), 3.92 (s, 3H).
^{13}C NMR (125 MHz, acetone-d_6) δ 165.5, 164.1, 150.5, 144.8, 130.5, 126.6, 125.8, 120.3, 120.0, 115.7, 112.6, 56.2.
GC-MS (EI, 70 eV) *m/z*: 259 ($[M]^+$)

5-Chloro-2-(4-fluororophenyl)benzoxazole[12]

Analytical TLC on silica gel, 1/19 acetone/petroleum ether.
White solid, Yield 75%, mp = 156-157 °C.

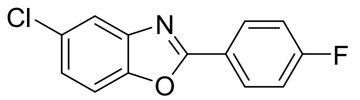

FT-IR (KBr, 4000 – 400 cm^{-1}): 3061, 2922, 1610, 1551, 1481, 1450, 1331, 1260, 1227.
^{1}H NMR (500 MHz, acetone-d_6) δ 8.31 (dd, J = 8.5, 5.5 Hz, 2H), 7.78 (s, 1H), 7.73 (d, J = 8.6 Hz, 1H), 7.47 – 7.38 (m, 3H).
^{13}C NMR (125 MHz, acetone-d_6): δ 166.0 (d, J = 250 Hz), 164.3, 150.5, 144.3, 131.1 (d, J = 9.0 Hz), 130.6, 126.3, 124.2, 120.5, 117.2 (d, J = 22.4 Hz), 112.7.
GC-MS (EI, 70 eV) *m/z*: 247 ($[M]^+$)

5-Chloro-2-(4-chlorophenyl)benzoxazole[10]

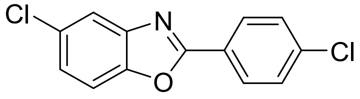

Analytical TLC on silica gel, 1/19 acetone/petroleum ether.
White solid, Yield 95%, mp = 190-192 °C.
FT-IR (KBr, 4000 – 400 cm^{-1}): 3061, 2925, 1610, 1549, 1542, 1480, 1449, 1260, 1197.
^{1}H NMR (500 MHz, acetone-d_6) δ 8.28 – 8.23 (m, 2H), 7.80 (d, J = 1.5 Hz, 1H), 7.74 (d, J = 8.5 Hz, 1H), 7.69 – 7.65 (m, 2H), 7.46 (dd, J = 8.5, 1.5 Hz, 1H).
^{13}C NMR (125 MHz, acetone-d_6): δ 164.6, 150.7, 144.4, 138.8, 130.9, 130.5, 130.3, 126.8, 126.6, 120.8, 113.0.
GC-MS (EI, 70 eV) *m/z*: 263

5-Chloro-2-(4-bromophenyl)benzoxazole[13]

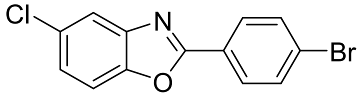

Analytical TLC on silica gel, 1/19 acetone/petroleum ether.
White solid, Yield 85%, mp = 191-192 °C.
FT-IR (KBr, 4000 – 400 cm^{-1}): 3064, 2922, 1733, 1608, 1589, 1548, 1477, 1449, 1331, 1260, 1228.
^{1}H NMR (500 MHz, acetone-d_6): δ 8.20 – 8.14 (m, 1H), 7.81 (m, 1H), 7.73 (d, J = 8.5 Hz, 1H), 7.45 (dd, J = 9.0, 2.0 Hz, 1H).
^{13}C NMR (125 MHz, acetone-d_6): δ 164.2, 150.4, 144.1, 133.2, 130.6, 130.1, 127.0, 126.7, 126.5, 120.5, 112.7.
GC-MS (EI, 70 eV) *m/z*: 308

5-Chloro-2-(4-hydroxyphenyl)benzoxazole[8]

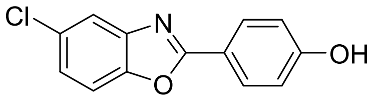

Analytical TLC on silica gel, 1/19 acetone/petroleum ether.
White solid, Yield 75%, mp = 185-187 °C.
FT-IR (KBr, 4000 – 400 cm^{-1}): 3162, 2920, 2850, 1666, 1596, 1553, 1446, 1448, 1333, 1286, 1217.
^{1}H NMR (500 MHz, acetone-d_6): δ 8.13 – 8.09 (m, 2H), 7.69 (dd, J = 8.5, 2.0 Hz, 2H), 7.38 (dd, J = 8.5, 2.0 Hz, 1H), 7.07 – 7.04 (m, 2H).
^{13}C NMR (126 MHz, acetone-d_6): δ 162.1, 150.2, 144.7, 130.6, 125.5, 121.6, 120.0, 118.9, 117.0, 112.4, 88.3.
GC-MS (EI, 70 eV) *m/z*: 245 ($[M]^+$)

5-Chloro-2-(pyridin-4-yl)benzoxazole[9]

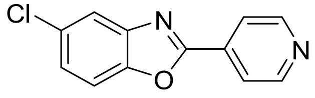

Analytical TLC on silica gel, 1/19 acetone/petroleum ether.
White solid, Yield 85%, mp = 152 - 153 °C.
FT-IR (KBr, 4000 – 400 cm^{-1}): 3431, 2922, 1612, 1568, 1539, 1449, 1057, 874.
^{1}H NMR (500 MHz, ($CDCl_3$): *δ* 8.83 (d, *J* = 5.5 Hz, 2H), 8.07 (dd, *J* = 4.5, 1.5 Hz, 2H), 7.80 (d, *J* = 2.0 Hz, 1H), 7.55 (d, *J* = 8.5 Hz, 1H), 7.40 (dd, *J* = 8.5, 2.0 Hz, 1H).
^{13}C NMR (125 MHz, ($CDCl_3$): *δ* 162.0, 150.8, 149.6, 143.0, 134.2, 130.9, 126.9, 121.3, 120.8, 111.9.
GC-MS (EI, 70 eV) *m/z*: 230 ([M]$^{+}$)

5-Nitro-2-phenylbenzoxazole[10]

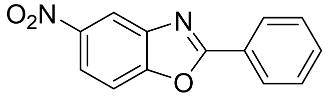

Analytical TLC on silica gel, 1/19 acetone/petroleum ether.
Yellow solid, Yield 70%, mp = 121 °C.
FT-IR (KBr, 4000 – 400 cm^{-1}): 3107, 2922, 2852, 1704, 1604, 1525, 1473, 1448, 1247, 1285.
^{1}H NMR (500 MHz, DMSO-d_6) *δ* 8.68 (d, *J* = 2.5 Hz, 1H), 8.35 (dd, *J* = 9.0, 2.0 Hz, 1H), 8.26 – 8.24 (m, 2H), 8.06 (d, *J* = 9.0 Hz, 1H), 7.71 – 7.65 (m, 3H).
^{13}C NMR (125 MHz, DMSO-d_6) *δ* 165.4, 154.0, 145.1, 141.9, 132.9, 129.5, 127.8 (d, *J* = 14.3 Hz), 125.5, 121.5 (d, *J* = 16.8 Hz), 115.7 (d, *J* = 12.5 Hz), 111.8.
GC-MS (EI, 70 eV) *m/z*: 240 ([M]$^{+}$)

5-Nitro-2-(*p*-tolyl)benzoxazole[14]

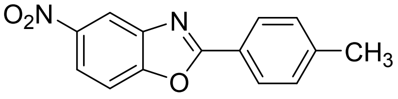

Analytical TLC on silica gel, 1/19 acetone/petroleum ether.
Yellow solid, Yield 75%, mp = 125-126 °C.
FT-IR (KBr, 4000 – 400 cm^{-1}): 3467, 3106, 2964, 2869, 1723, 1531, 1462, 1420, 1130.
^{1}H NMR (500 MHz, acetone-d_6) *δ* 8.31 – 8.27 (m, 2H), 7.57 – 7.55 (m, 2H), 7.40 – 7.35 (m, 2H), 7.24 (d, *J* = 9.0 Hz, 1H), 2.47 (s, 3H).
^{13}C NMR (125 MHz, acetone-d_6) *δ* 166.7, 164.7, 150.0, 143.3, 135.5, 130.8, 130.7, 127.30, 124.8, 120.7, 117.2, 117.0, 110.9, 21.4.
GC-MS (EI, 70 eV) *m/z*: 254 ([M]$^{+}$)

5-Nitro-2-(4-*tert*-butylphenyl)benzoxazole[15]

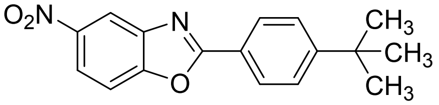

Analytical TLC on silica gel, 1/19 acetone/petroleum ether.
White solid, Yield 75%, mp = 154-156 °C.
FT-IR (KBr, 4000 – 400 cm^{-1}): 3106, 2964, 2869, 1723, 1531, 1462, 1410, 1267.
^{1}H NMR (500 MHz, acetone-d_6) *δ* 8.18 – 8.16 (m, 2H), 7.66 – 7.63 (m, 2H), 7.56 – 7.54 (m, 2H), 7.23 – 7.21 (m, 1H), 1.38 (s, 9H).
^{13}C NMR (125 MHz, acetone-d_6) *δ* 166.9, 157.3, 155.3, 146.4, 143.6, 132.1, 129.7, 128.7, 127.2, 124.2, 122.0, 116.4, 112.1, 35.8, 31.4.
GC-MS (EI, 70 eV) *m/z*: 296 ([M]$^{+}$)

5-Nitro-2-(4-methoxyphenyl)benzoxazole[10]

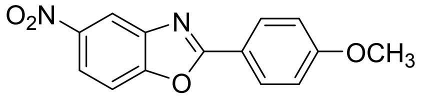

Analytical TLC on silica gel, 1/19 acetone/petroleum ether.
Yellow solid, Yield 80%, mp = 182-183 °C.
FT-IR (KBr, 4000 – 400 cm^{-1}): 3457, 2964, 1723, 1631, 1462, 1410, 1267.
^{1}H NMR (500 MHz, acetone-d_6) δ 8.55 (d, J = 2.5 Hz, 1H), 8.34 (dd, J = 9.0, 2.5 Hz, 1H), 8.25 – 8.23 (m, 2H), 7.91 (d, J = 9.0 Hz, 1H), 7.20 – 7.18 (m, 2H), 3.95 (s, 3H).
^{13}C NMR (125 MHz, acetone-d_6) δ 163.5, 154.4, 142.8, 129.8, 120.8, 118.3, 115.2, 114.8, 111.0, 55.2.
GC-MS (EI, 70 eV) *m/z*: 270 ([M]$^+$)

5-Nitro-2-(4-fluorophenyl)benzoxazole[14]

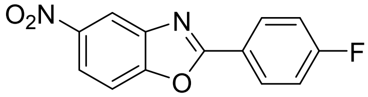

Analytical TLC on silica gel, 1/19 acetone/petroleum ether.
White solid, Yield 72%, mp = 202-205 °C.
FT-IR (KBr, 4000 – 400 cm^{-1}): 3061, 2925, 1650, 1584, 1582, 1542, 1473, 1448, 1247, 1225.
^{1}H NMR (500 MHz, acetone-d_6): δ 8.60 (d, J = 2.3 Hz, 1H), 8.39 – 8.35 (m, 3H), 7.96 (d, J = 8.9 Hz, 1H), 7.47 – 7.41 (m, 2H).
^{13}C NMR (125 MHz, acetone-d_6): δ 166.2 (d, J = 250.8 Hz), 155.3, 131.3 (d, J = 9.3 Hz), 123.5, 122.0, 117.3 (d, J = 22.5 Hz), 116.5, 112.1.
GC-MS (EI, 70 eV) *m/z*: 258 ([M]$^+$)

5-Nitro-2-(4-chlorophenyl)benzoxazole[10]

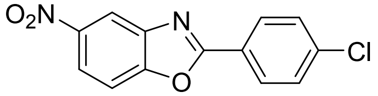

Analytical TLC on silica gel, 1/19 acetone/petroleum ether.
White solid, Yield 80%, mp = 207-210 °C.
FT-IR (KBr, 4000 – 400 cm^{-1}): 3420, 3105, 2964, 2853, 1723, 1528, 1434, 1410, 1256.
^{1}H NMR (500 MHz, acetone-d_6): δ 8.62 (d, J = 2.0 Hz, 1H), 8.39 (dd, J = 8.5, 2.0 Hz, 1H), 8.31 – 8.29 (m, 2H), 7.97 (d, J = 9 Hz, 1H), 7.72 – 7.68 (m, 2H).
^{13}C NMR (125 MHz, acetone-d_6): δ 155.4, 139.3, 130.4, 130.3, 129.5, 125.3, 122.2, 116.6, 114.8, 112.2, 103.5.
GC-MS (EI, 70 eV) *m/z*: 274 ([M]$^+$)

5-Nitro-2-(4-bromophenyl)benzoxazole[16]

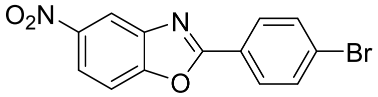

Analytical TLC on silica gel, 1/19 acetone/petroleum ether.
Yellow solid, Yield 85%, mp = 210-212 °C.
FT-IR (KBr, 4000 – 400 cm^{-1}): 3061, 2925, 1650, 1584, 1582, 1542,

1473, 1448, 1247, 1225.
^{1}H NMR (500 MHz, acetone-d_6): δ 7.78 – 7.55 (m, 5H), 7.33 (d, J = 16.5 Hz, 1H), 6.81 (d, J = 16.0 Hz, 1H).
^{13}C NMR (125 MHz, acetone-d_6): δ 142.0 (d, J = 11.3), 135.1 (d, J = 26.3), 133.4, 132.9 (d, J = 3.8), 132.5, 130.8 (d, J =17.5), 130.4, 128.7, 127.1, 124.7, 124.6.
GC-MS (EI, 70 eV) *m/z*: 318 ($[M]^+$)

2-Phenylbezothiazole[17]

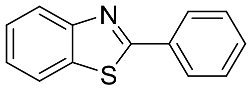

Analytical TLC on silica gel, 1/19 ethyl acetate/hexane.
White solid, Yield 85%, mp = 125 °C.
FT-IR (KBr, 4000 – 400 cm^{-1}): 3435, 3063, 1508, 1283, 1027, 763.
^{1}H NMR (500 MHz, $CDCl_3$) δ 8.12 – 8.08 (m, 3H), 7.91 (d, J = 8.0 Hz, 1H), 7.52 – 7.48 (m, 4H), 7.41 – 7.36 (m, 1H).
^{13}C NMR (125 MHz, $CDCl_3$): δ 168.2, 154.3, 135.2, 133.8, 131.1, 129.2, 127.7, 126.5, 125.3, 123.4, 121.6.
GC-MS (EI, 70 eV) *m/z*: 211 ($[M]^+$)

2-(4-Methoxyphenyl)benzothiazole[17]

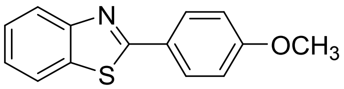

Analytical TLC on silica gel, 1/19 ethyl acetate/hexane.
White solid, Yield 88%, mp = 123 °C.
FT-IR (KBr, 4000 – 400 cm^{-1}): 3408, 2926, 1599, 1478, 1255, 1023, 830.
^{1}H NMR (500 MHz, $CDCl_3$): δ 8.06 – 8.01 (m, 3H), 7.88 (d, J = 8.0 Hz, 1H), 7.49 – 7.45 (m, 1H), 7.38 – 7.33 (m, 1H), 7.02 – 6.98 (m, 2H), 3.89 (s, 3H).
^{13}C NMR (125 MHz, $CDCl_3$): δ 168.0, 162.1, 154.3, 135.0, 129.3, 126.6, 126.4, 125.0, 123.0, 121.7, 114.5, 55.6.
GC-MS (EI, 70 eV) *m/z*: 241 ($[M]^+$)

2-(4-Methylphenyl)benzothiazole[17]

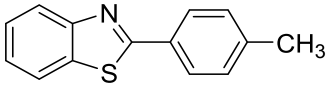

Analytical TLC on silica gel, 1/19 ethyl acetate/hexane.
White solid, Yield 90%, mp = 95 – 96 °C.
FT-IR (KBr, 4000 – 400 cm^{-1}): 3427, 2919, 1605, 1477, 1216.
^{1}H NMR (500 MHz, $CDCl_3$): δ 8.10 – 8.04 (m, 1H), 7.99 (d, J = 8.0 Hz, 2H), 7.88 (dd, J = 8.0, 0.5 Hz, 1H), 7.50 – 7.46 (m, 1H), 7.39 – 7.35 (m, 1H), 7.29 (d, J = 8.0 Hz, 2H), 2.42 (s, 3H).
^{13}C NMR (125 MHz, $CDCl_3$): δ 168.3, 154.3, 141.5, 135.1, 131.1, 129.8, 127.6, 126.4, 125.1, 123.2, 121.7, 21.6.
GC-MS (EI, 70 eV) *m/z*: 225 ($[M]^+$)

2-(4-Fluorophenyl)benzothiazole[18]

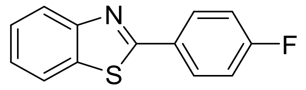

Analytical TLC on silica gel, 1/19 ethyl acetate/hexane.
White solid, Yield 85%, mp = 110 ^{o}C.
FT-IR (KBr, 4000 – 400 cm^{-1}): 3430, 2922, 1639, 1480, 1226, 964.
^{1}H NMR (500 MHz, $CDCl_3$): δ 8.10 – 8.05 (m, 3H), 7.89 (d, J = 8.0 Hz, 1H), 7.52 – 7.48 (m, 1H), 7.41 – 7.37 (m, 1H), 7.20 – 7.16 (m, 2H).
^{13}C NMR (125 MHz, $CDCl_3$): δ 166.9, 164.6 (d, J = 250.4 Hz), 154.3, 135.2, 130.1 (d, J = 3.3 Hz), 129.7 (d, J = 8.6 Hz), 126.5, 125.4, 123.4, 121.7, 116.3 (d, J = 22 Hz)
GC-MS (EI, 70 eV) *m/z*: 229 ($[M]^+$)

2-(4-Chlorophenyl)benzothiazole[17]

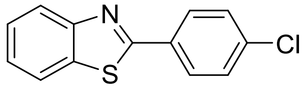

Analytical TLC on silica gel, 1/19 ethyl acetate/hexane.
White solid, Yield 80%, mp = 119 ^{o}C.
FT-IR (KBr, 4000 – 400 cm^{-1}): 3432, 3053, 1635, 1505, 1311, 1086, 826.
^{1}H NMR (500 MHz, $CDCl_3$) δ 8.09 – 8.05 (m, 1H), 8.04 – 8.01 (m, 2H), 7.90 (d, J = 8.0 Hz, 1H), 7.52 – 7.44 (m, 3H), 7.40 (m, 1H).
^{13}C NMR (125 MHz, $CDCl_3$): δ 166.8, 154.3, 137.2, 135.2, 132.3, 129.4, 128.9, 126.6, 125.6, 123.5, 121.8.
GC-MS (EI, 70 eV) *m/z*: 245 ($[M]^+$)

2-(4-Nitrophenyl)benzothiazole[19]

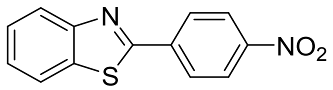

Analytical TLC on silica gel, 1/19 ethyl acetate/hexane.
Yellow solid, Yield 70%, mp = 207 ^{o}C.
FT-IR (KBr, 4000 – 400 cm^{-1}): 3429, 2922, 1518, 1339, 1101, 965.
^{1}H NMR (500 MHz, $CDCl_3$): δ 8.37 – 8.34 (m, 2H), 8.29 – 8.26 (m, 2H), 8.13 (d, J = 8.0 Hz, 1H), 7.96 (d, J = 8.0 Hz, 1H), 7.58 – 7.53 (m, 1H), 7.48 – 7.45 (m, 1H).
^{13}C NMR (125 MHz, $CDCl_3$): δ 165.0, 154.3, 149.2, 139.3, 135.7, 128.4, 127.1, 126.4, 124.5, 124.1, 122.0.
GC-MS (EI, 70 eV) *m/z*: 256 ($[M]^+$)

Section S4. References

1. Teo, Y. C.; Riduan, S. N.; Zhang, Y. *Green Chem.* **2013,** *15*, 2365-2368.
2. Tang, L.; Guo, X.; Yang, Y.; Zha, Z.; Wang, Z. *Chem. Commun. (Camb)* **2014,** *50*, 6145-6148.
3. Patil, M. R.; Bhanushali, J. T.; Nagaraja, B. M.; Keri, R. S. *C. R. Chimie* **2017**.

4. Zhou, Q.; Zhang, J. F.; Cao, H.; Zhong, R.; Hou, X. F. *J. Org. Chem.* **2016,** *81*, 12169-12180.
5. Gupta, R.; Sahu, P. K.; Sahu, P. K.; Srivastava, S. K.; Agarwal, D. D. *Catal. Communi.* **2017,** *92*, 119-123.
6. Wu, A.; Chen, Q.; Liu, W.; You, L.; Fu, Y.; Zhang, H. *Org. Lett.* **2017**.
7. Steinberg, D. F.; Turk, M. C.; Kalyani, D. *Tetrahedron* **2017,** *73*, 2196-2209.
8. Huang, L.; Zhang, W.; Zhang, X.; Yin, L.; Chen, B.; Song, J. *Bioorg. Med. Chem. Lett.* **2015,** *25*, 5299-5305.
9. Yamada, S.; Murakami, K.; Itami, K. *Org. Lett.* **2016,** *18*, 2415-2418.
10. Srivastava, A.; Shukla, G.; Singh, M. S. *Tetrahedron* **2017,** *73*, 879-887.
11. Reyes, H.; Beltran, H. I.; Rivera-Becerril, E. *Tetrahedron Lett.* **2011,** *52*, 308-310.
12. Prakash, O.; Batra, A.; Sharma, V.; Saini, R. K.; Verma, R. S. *J. Indian Chem. Soc.* **2003,** *80*, 1031-1034.
13. Wang, L.; Ma, Z.-G.; Wei, X.-J.; Meng, Q.-Y.; Yang, D.-T.; Du, S.-F.; Chen, Z.-F.; Wu, L.-Z.; Liu, Q. *Green Chem.* **2014,** *16*, 3752-3757.
14. Vosooghi, M.; Arshadi, H.; Saeedi, M.; Mahdavi, M.; Jafapour, F.; Shafiee, A.; Foroumadi, A. *J. Fluorine Chem.* **2014,** *161*, 83-86.
15. Yalçın, İ.; Şener, E.; Özden, T.; Özden, S.; Akın, A. *Eur. J. Med. Chem.* **1990,** *25*, 705-708.
16. Szlachcic, P.; Fedorchuk, A. A.; Danel, A.; Jarosz, B.; El Naggar, A. M.; Albassam, A. A.; Wojciechowski, A.; Gondek, E.; Uchacz, T.; Stadnicka, K.; Lakshminarayana, G.; Kityk, I. V. *Dyes Pigments* **2017,** *141*, 333-341.
17. Nguyen, L. A.; Ngo, Q. A.; Retailleau, P.; Nguyen, T. B. *Green Chem.* **2017,** *19*, 4289-4293.
18. Wang, X.; Miao, D.; Li, X.; Hu, R.; Yang, Z.; Gu, R.; Han, S. *Tetrahedron* **2017,** *73*, 5194-5199.
19. Wang, D.; Albero, J.; García, H.; Li, Z. *J. Catal.* **2017,** *349*, 156-162.

Section S5. NMR spectra

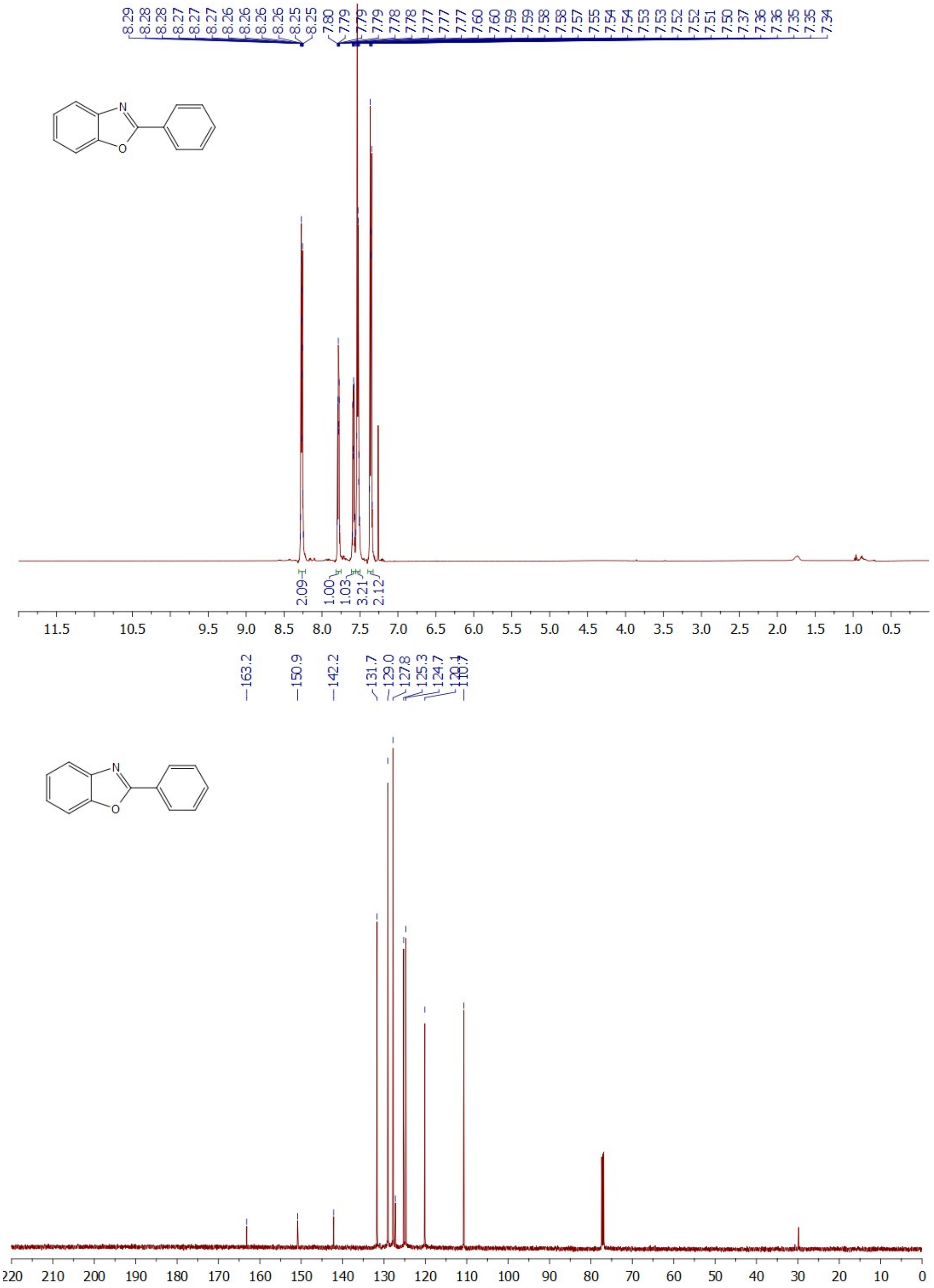

Figure 1. ^{1}H (top) and ^{13}C (bottom) NMR spectra of 2-Phenylbenzoxazole

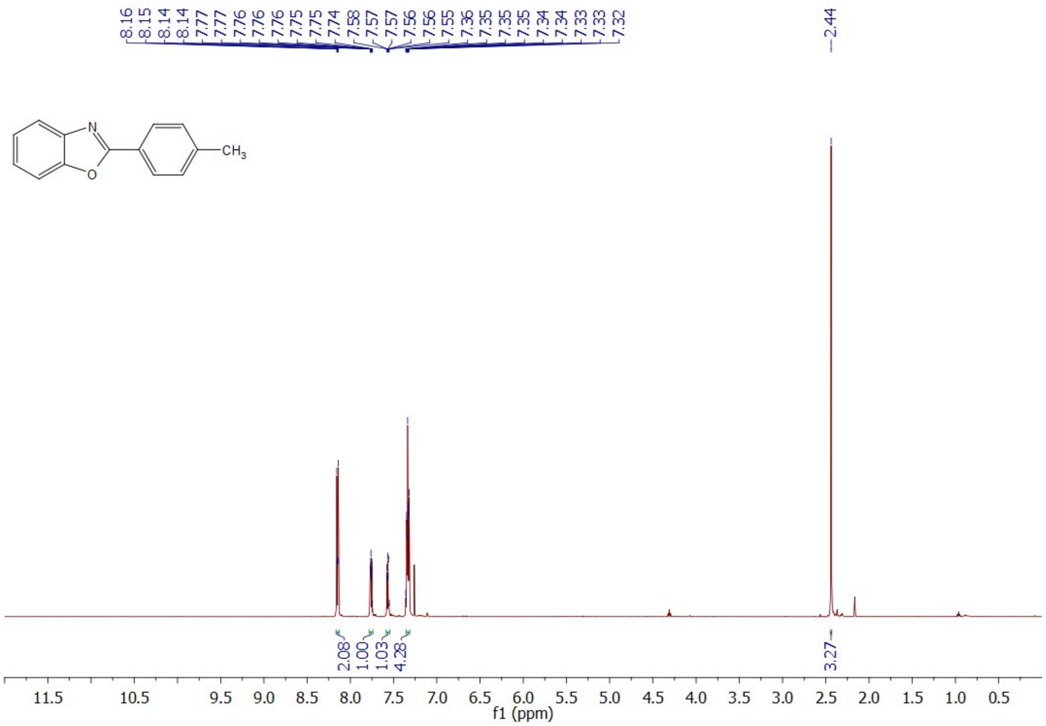

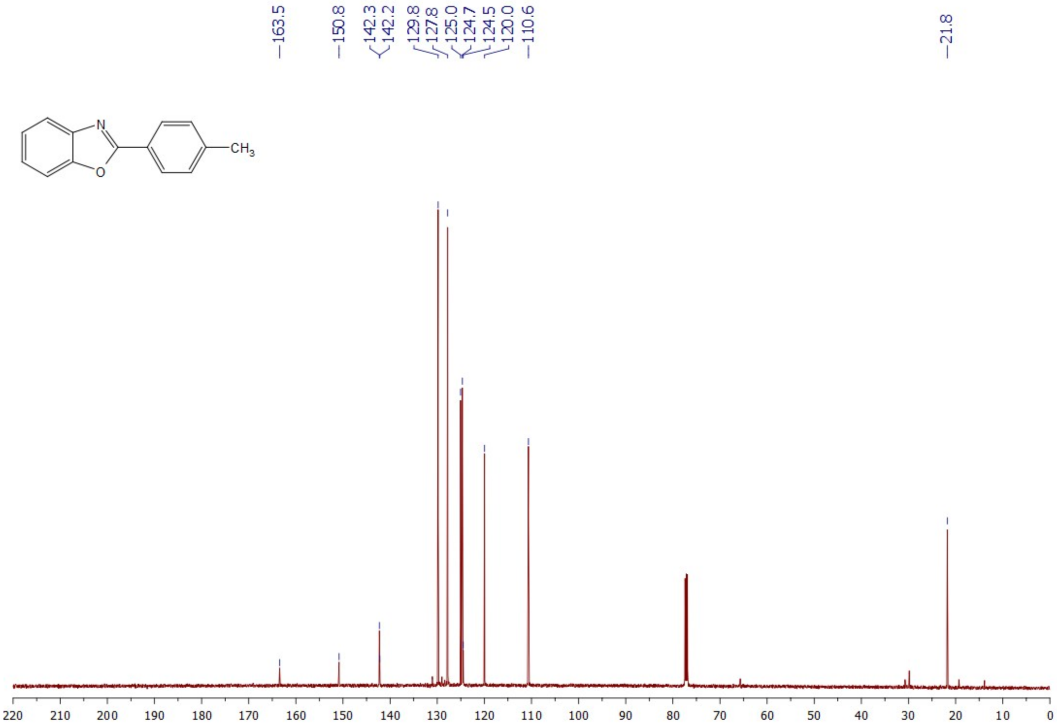

Figure 2. ^{1}H (top) and ^{13}C (bottom) NMR spectra of 2-(4-Methylphenyl)benzoxazole

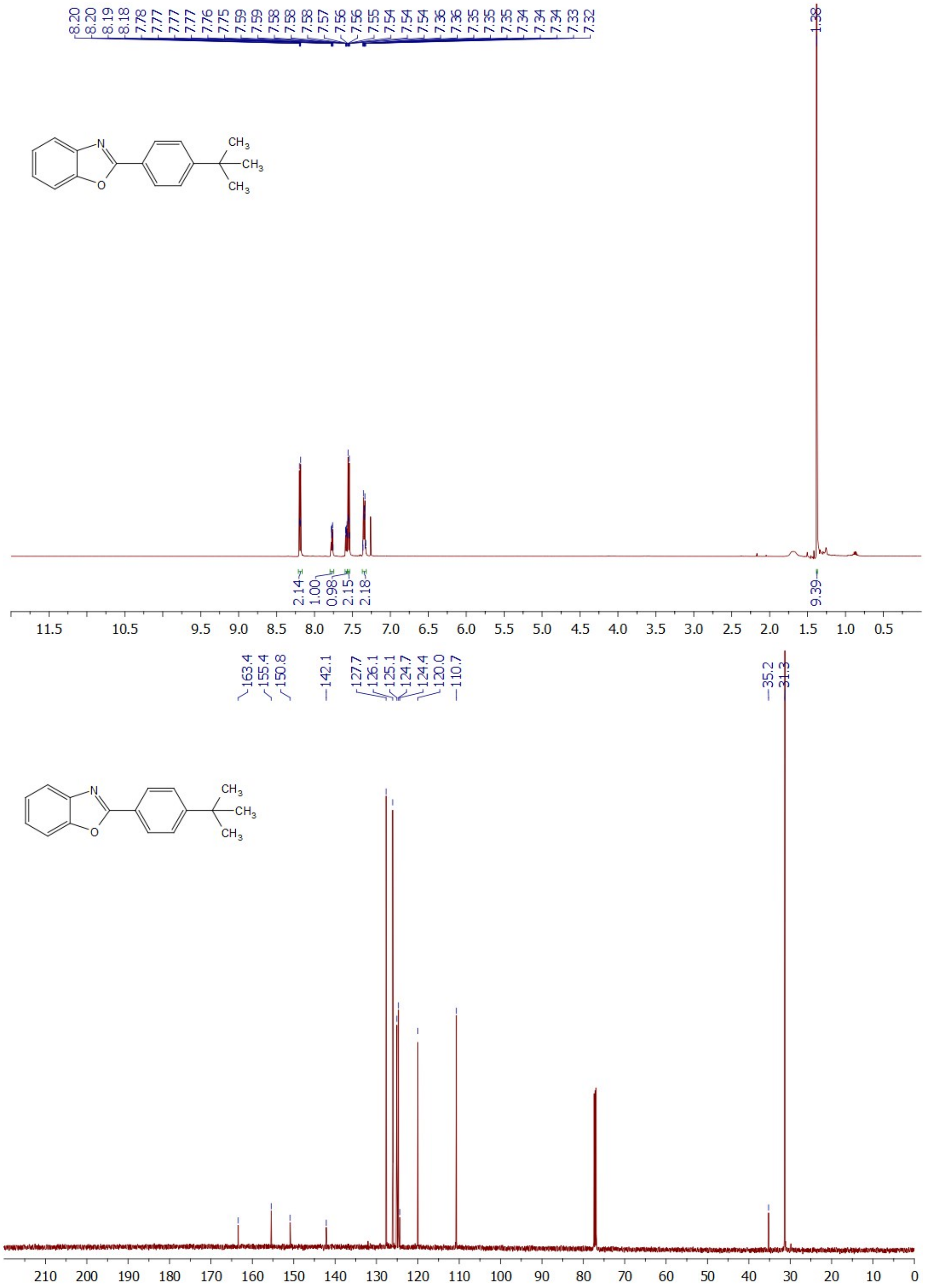

Figure 3. ^{1}H (top) and ^{13}C (bottom) NMR spectra of 2-(4-*tert*-butylphenyl)benzoxazole

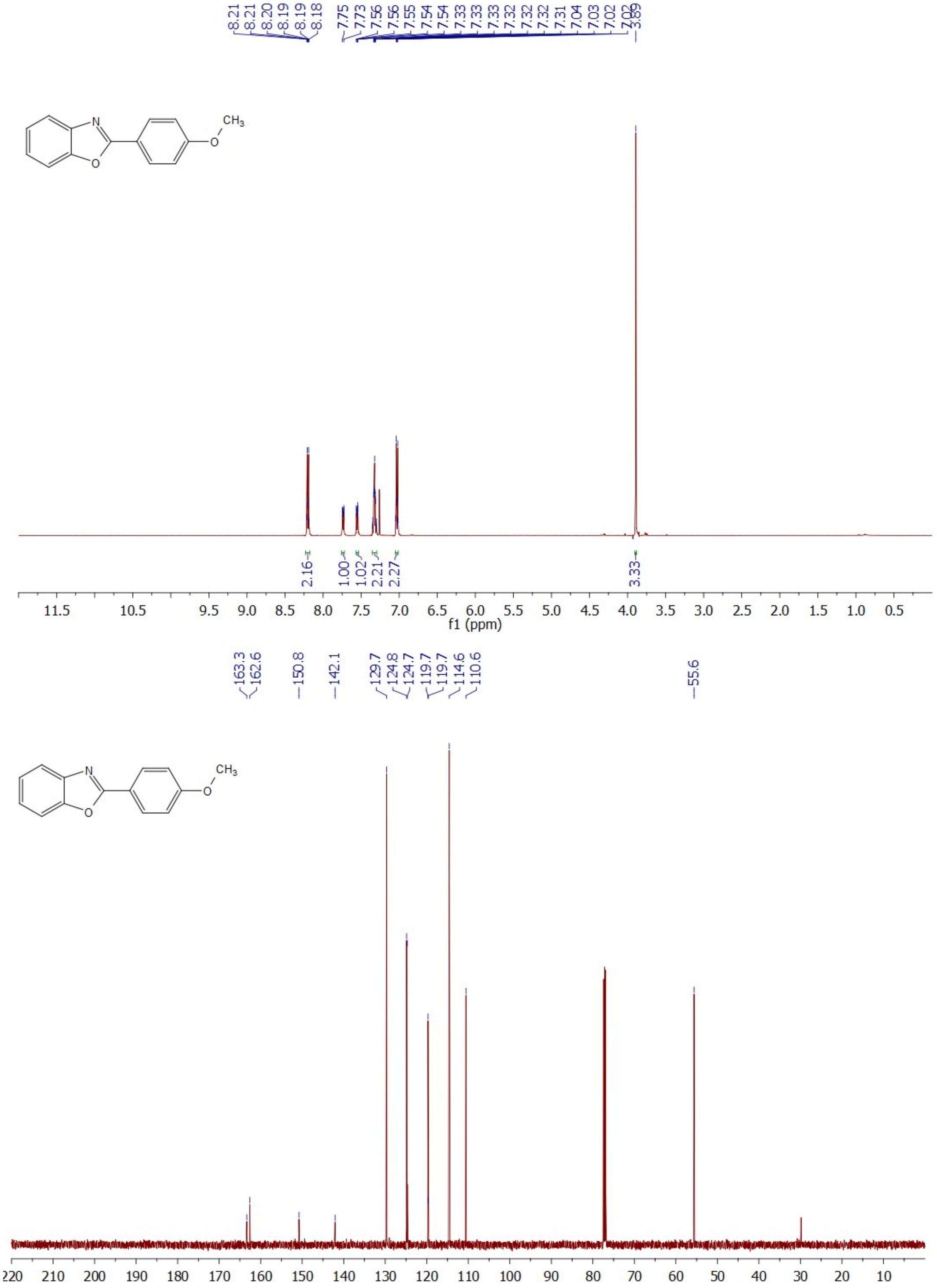

Figure 4. ^{1}H (top) and ^{13}C (bottom) NMR spectra of 2-(4-Methoxyphenyl)benzoxazole

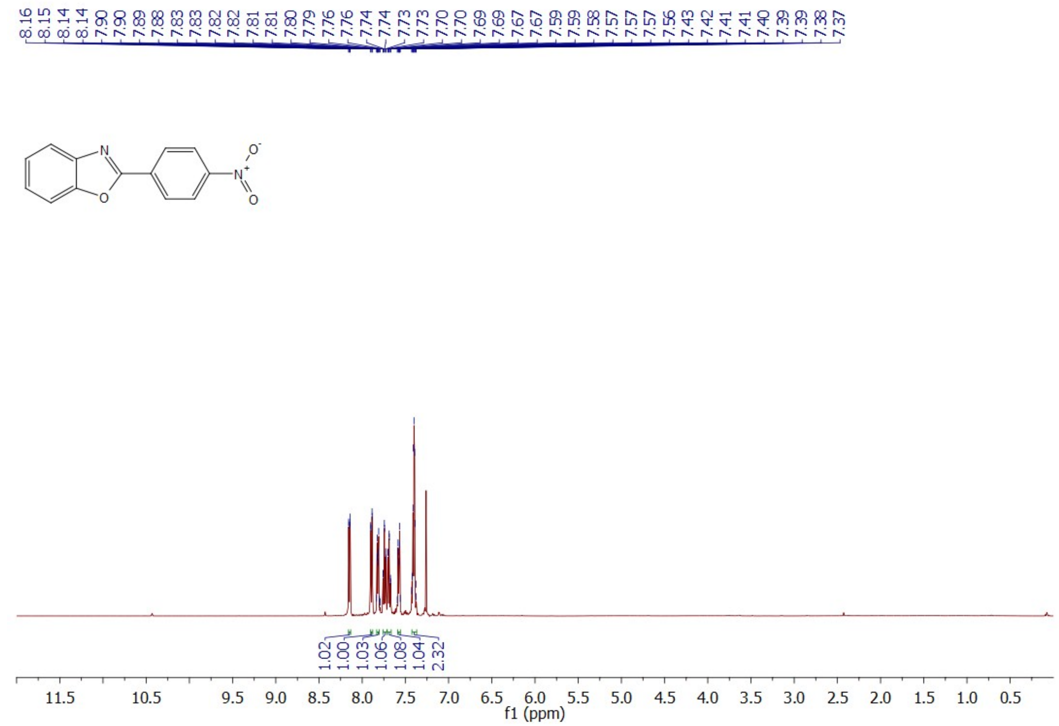

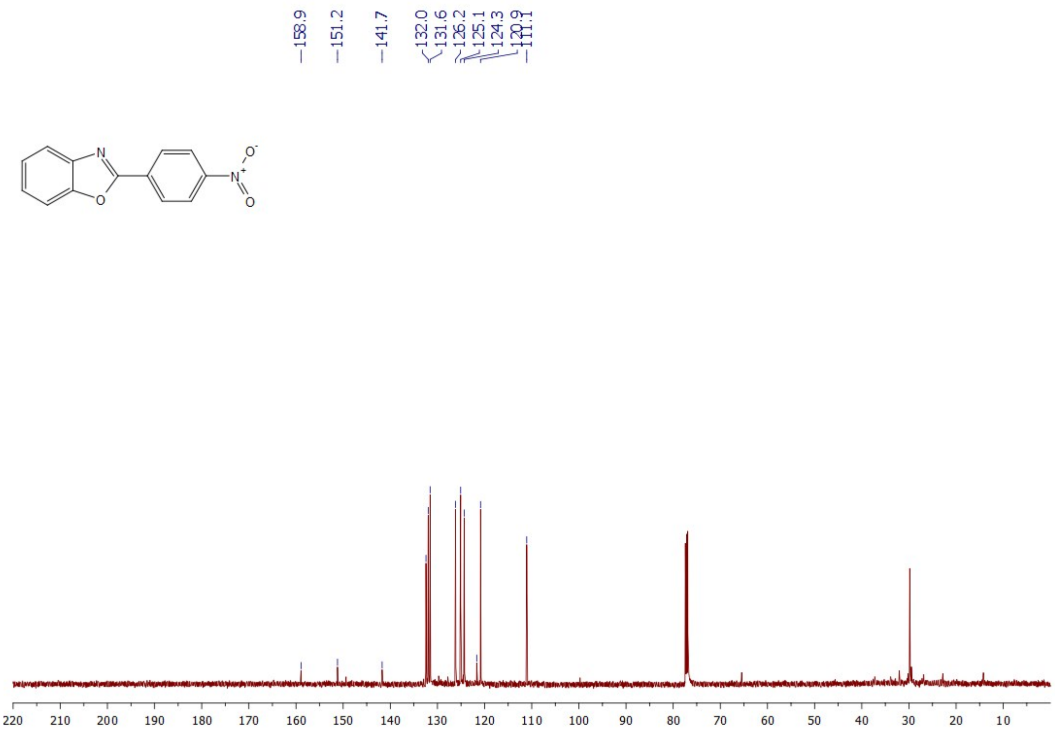

Figure 5. ^{1}H (top) and ^{13}C (bottom) NMR spectra of 2-(4-Nitrophenyl)benzoxazole

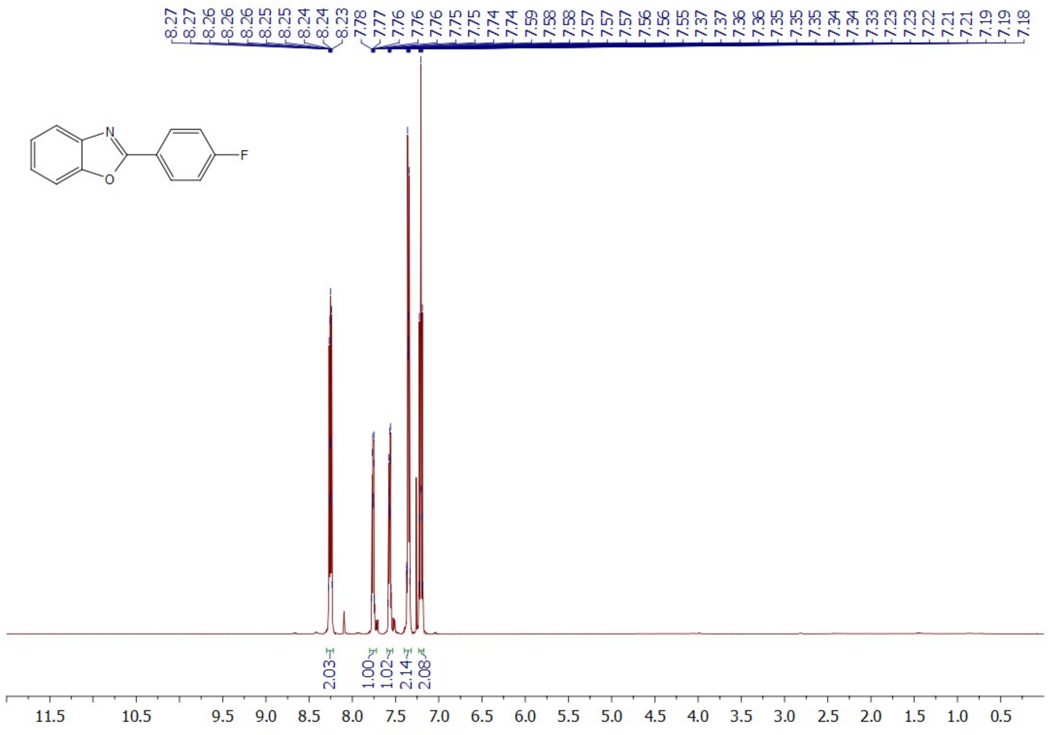

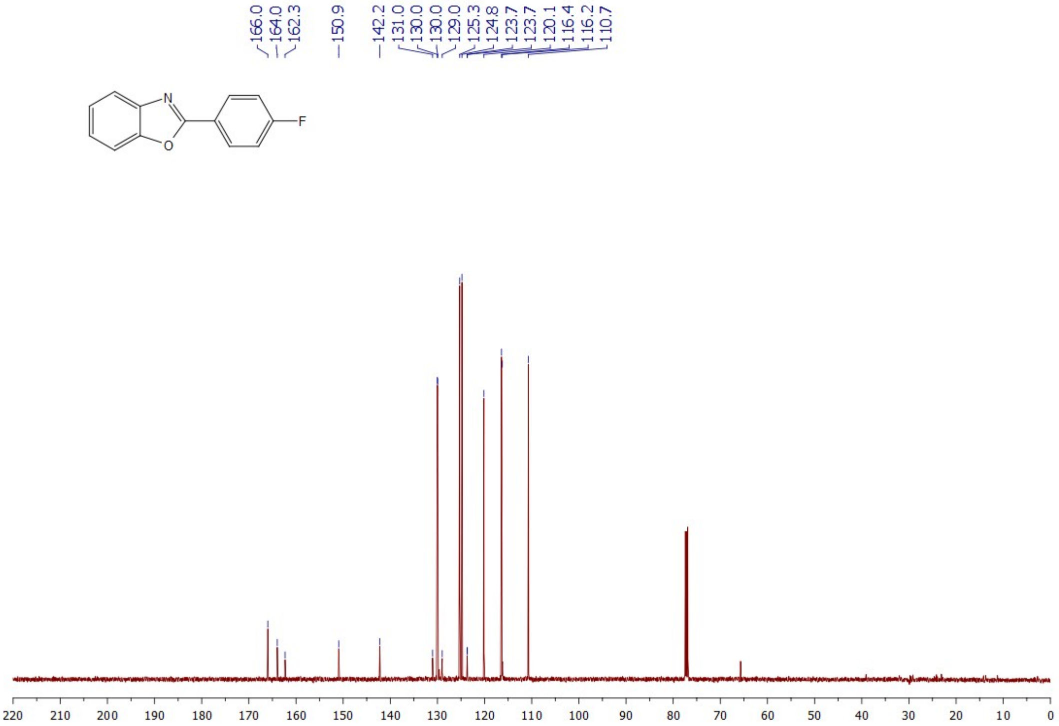

Figure 6. ^{1}H (top) and ^{13}C (bottom) NMR spectra of 2-(4-Fluorophenyl)benzoxazole

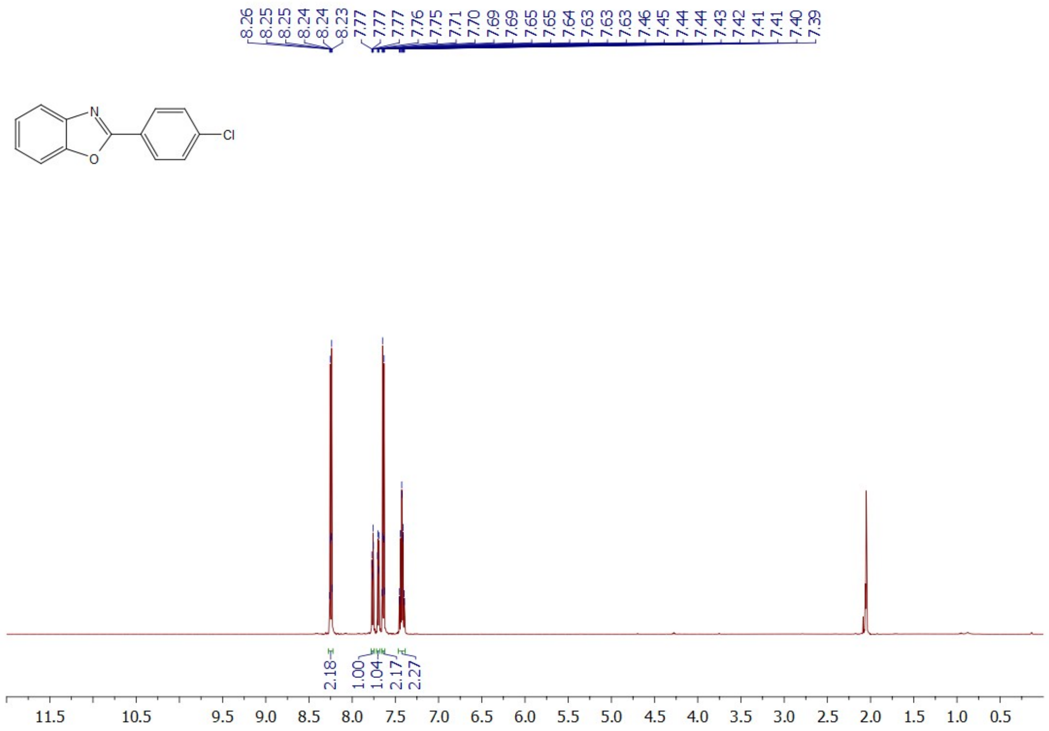

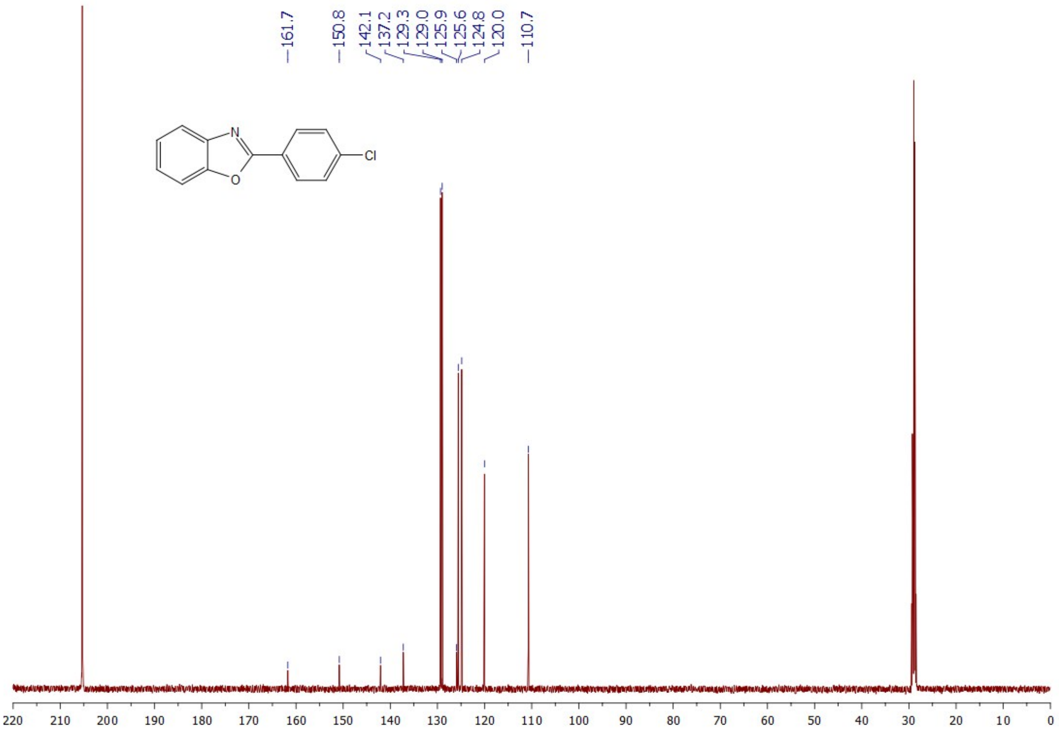

Figure 7. ^{1}H (top) and ^{13}C (bottom) NMR spectra of 2-(4-Chlorophenyl)benzoxazole

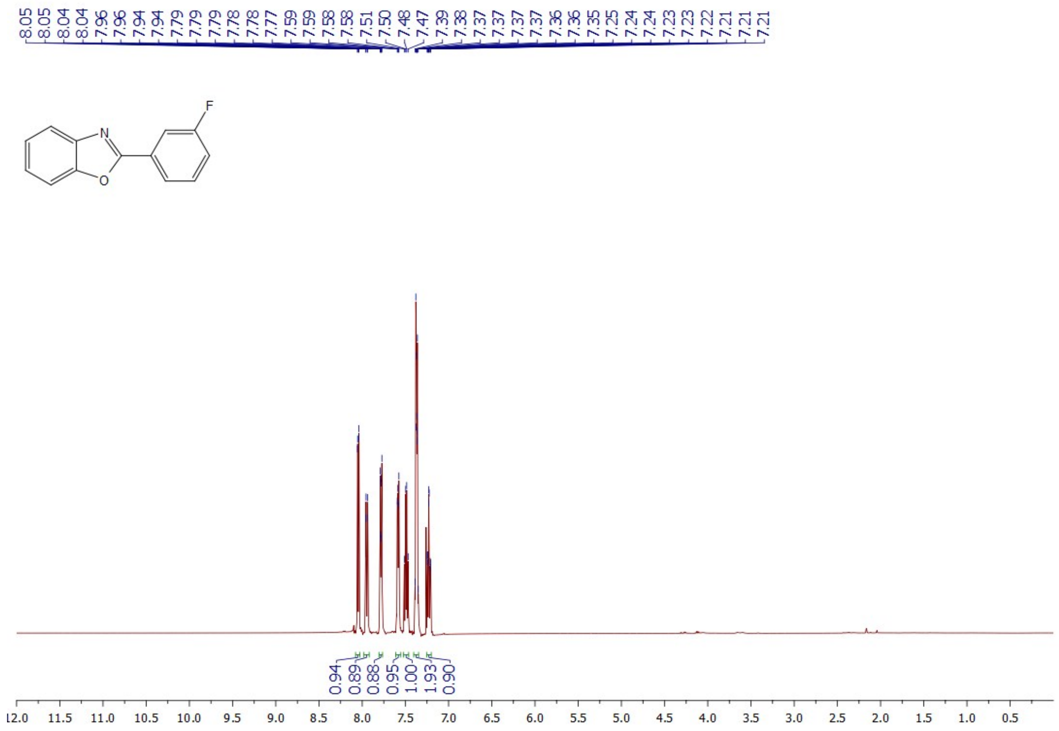

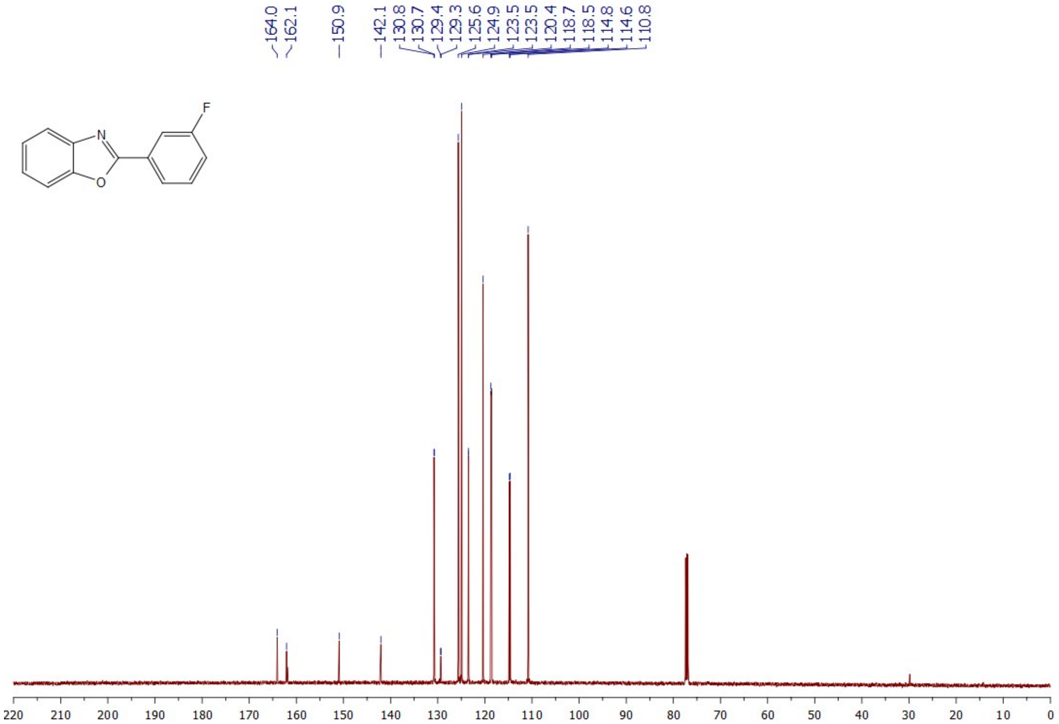

Figure 8. ^{1}H (top) and ^{13}C (bottom) NMR spectra of 2-(3-Fluorophenyl)benzoxazole

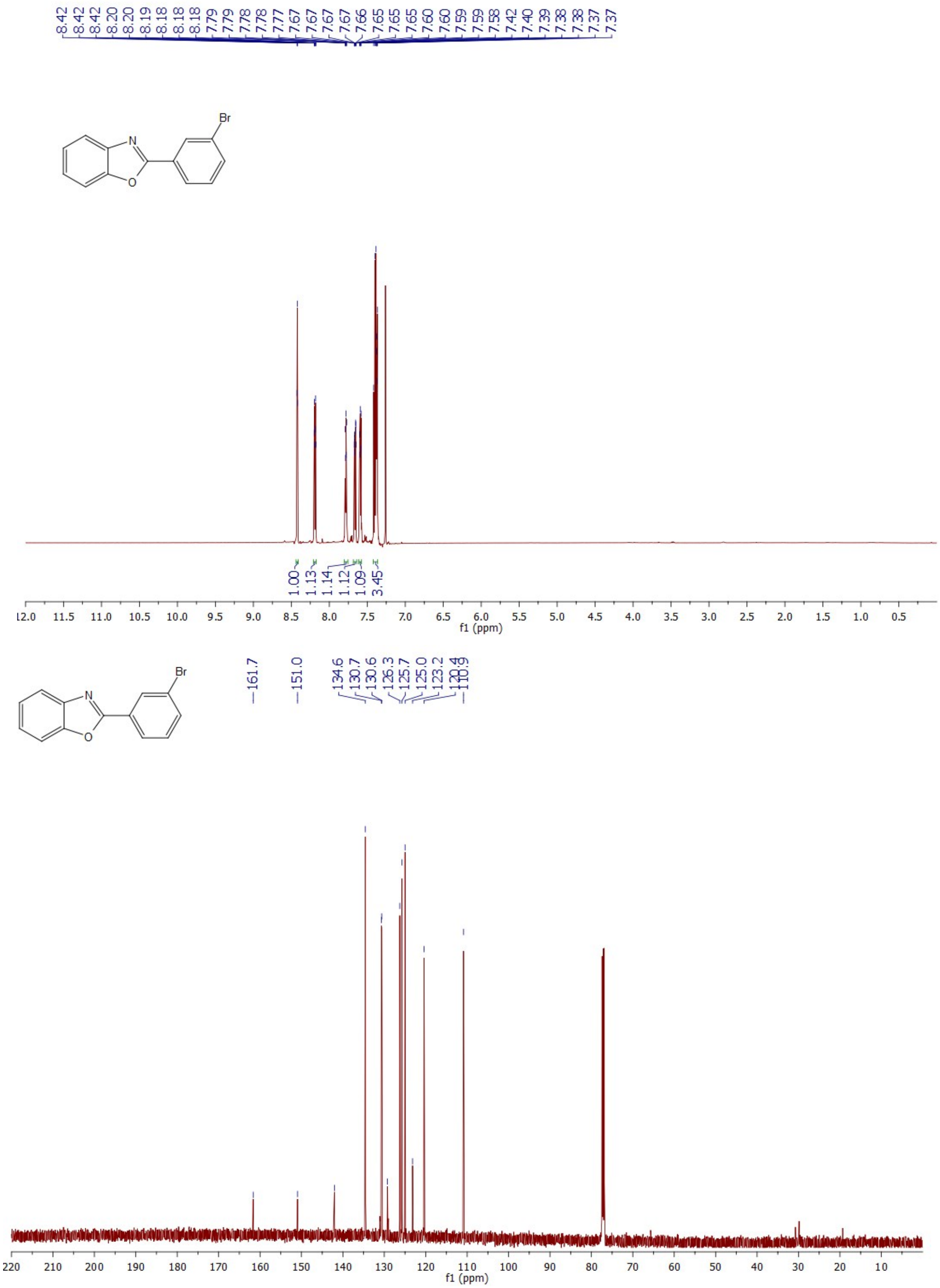

Figure 9. ^{1}H (top) and ^{13}C (bottom) NMR spectra of 2-(3-Bromophenyl)benzoxazole

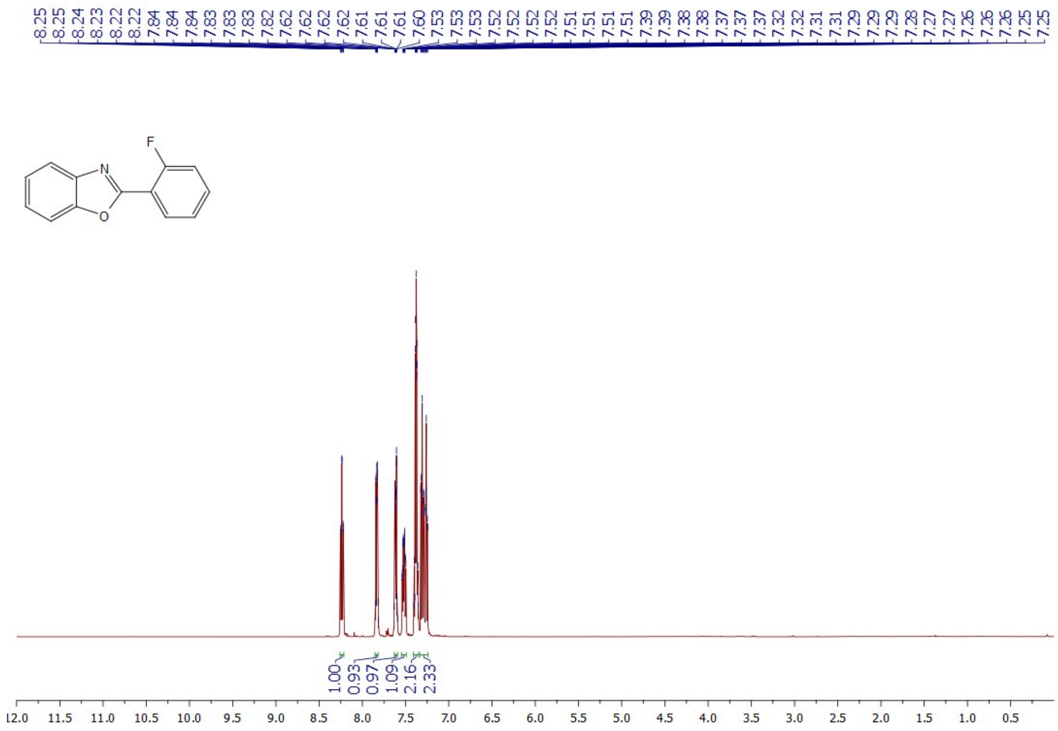

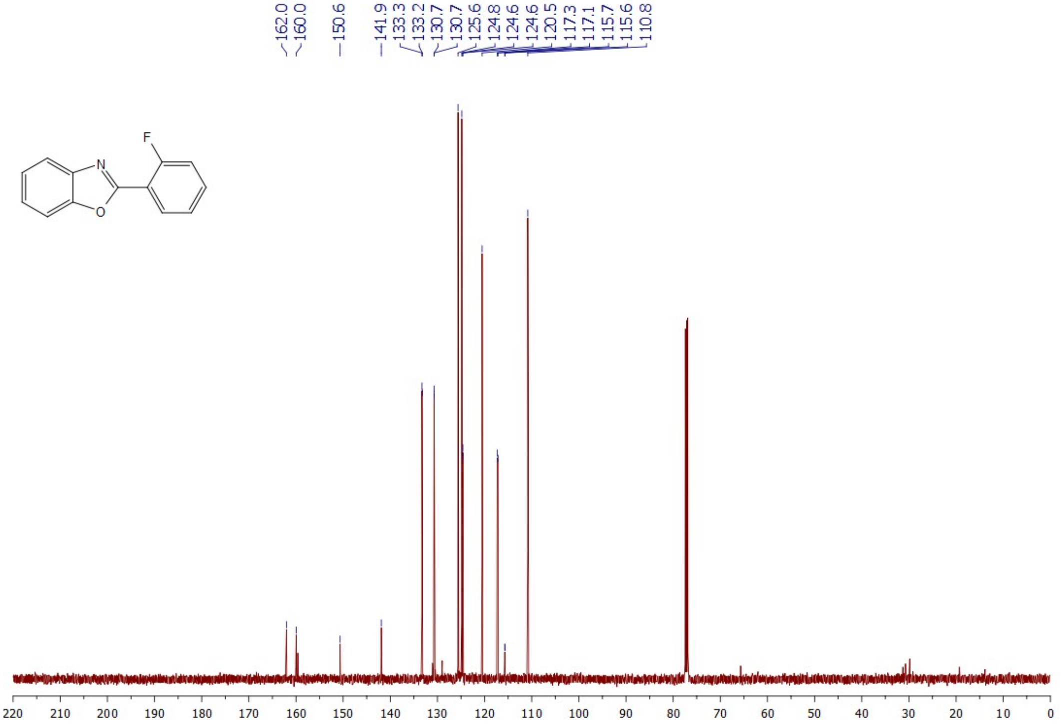

Figure 10. ^{1}H (top) and ^{13}C (bottom) NMR spectra of 2-(2-Fluorophenyl)benzoxazole

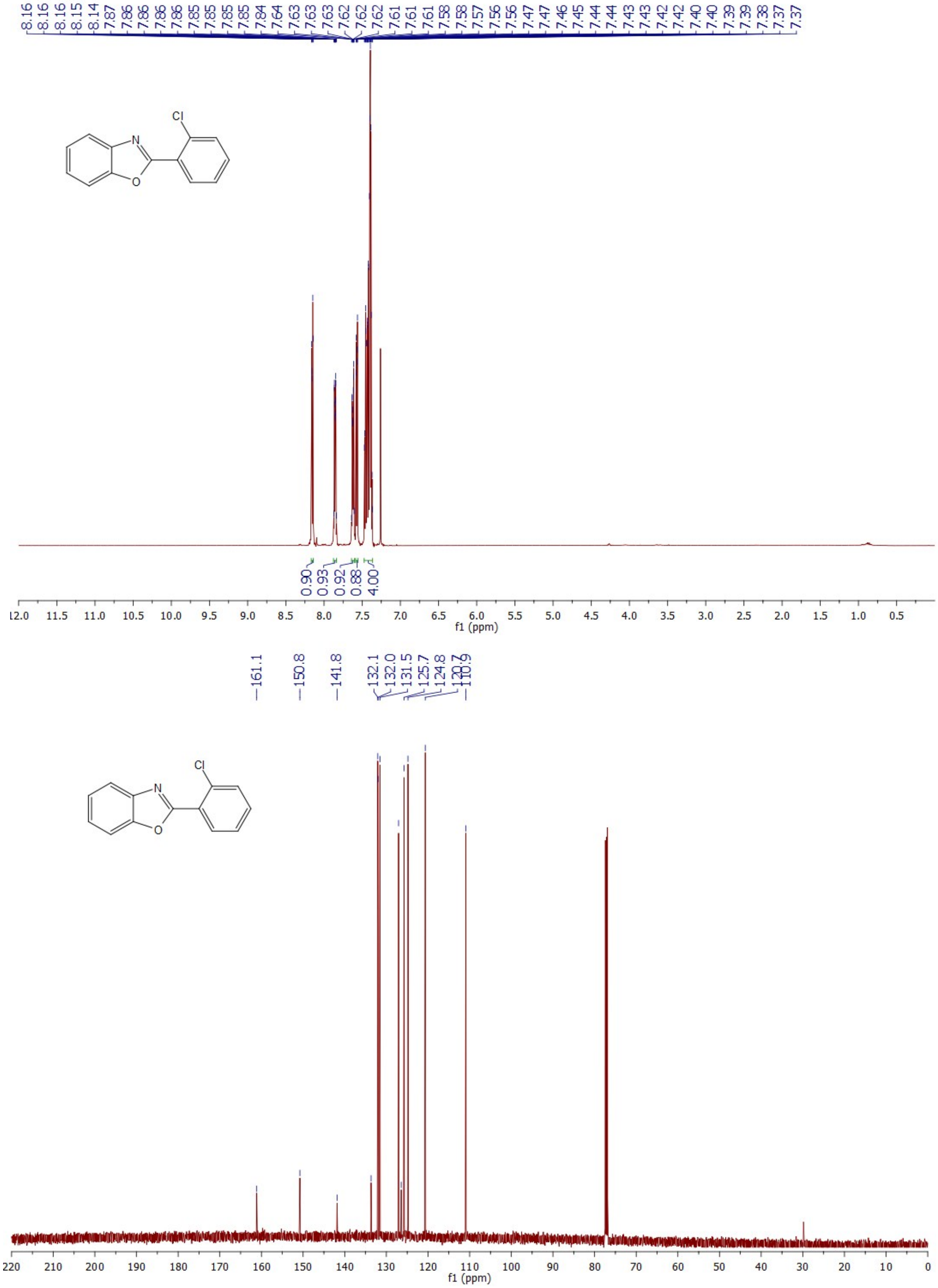

Figure 11. ^{1}H (top) and ^{13}C (bottom) NMR spectra of 2-(2-Chlorophenyl)benzoxazole

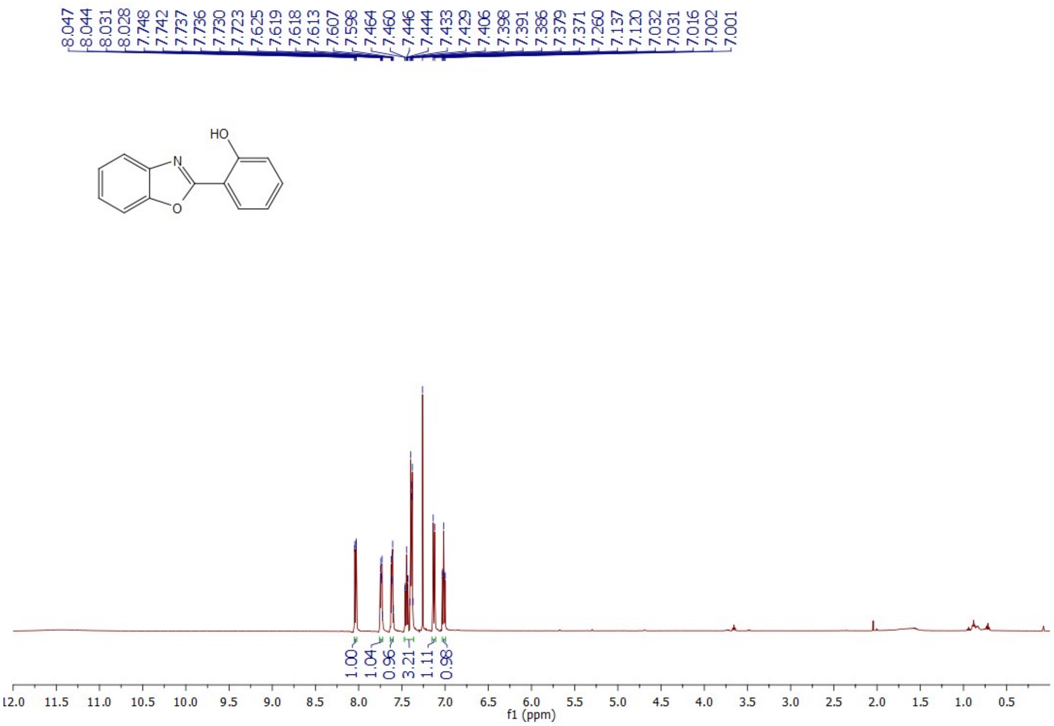

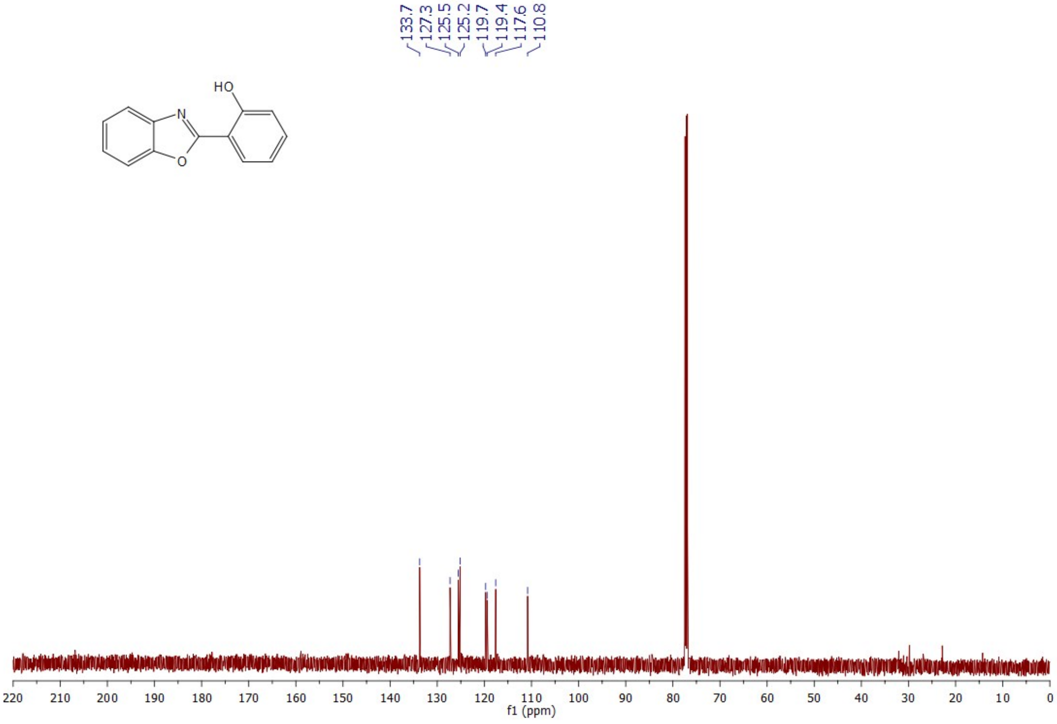

Figure 12. ^{1}H (top) and ^{13}C (bottom) NMR spectra of 2-(2-Hydroxyphenyl)benzoxazole

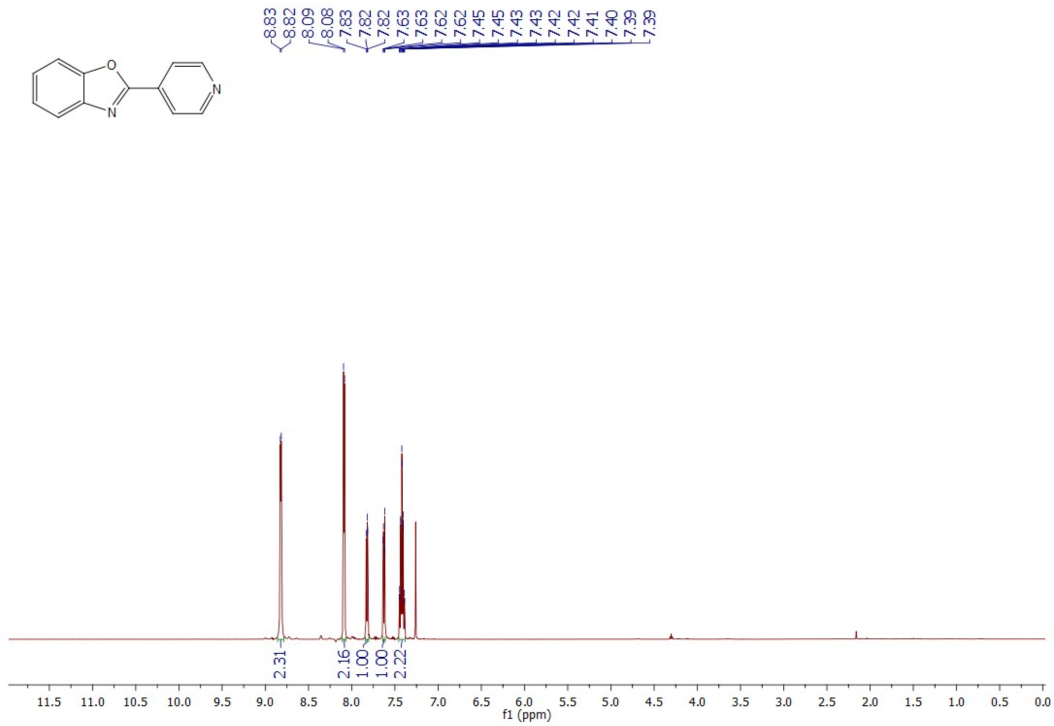

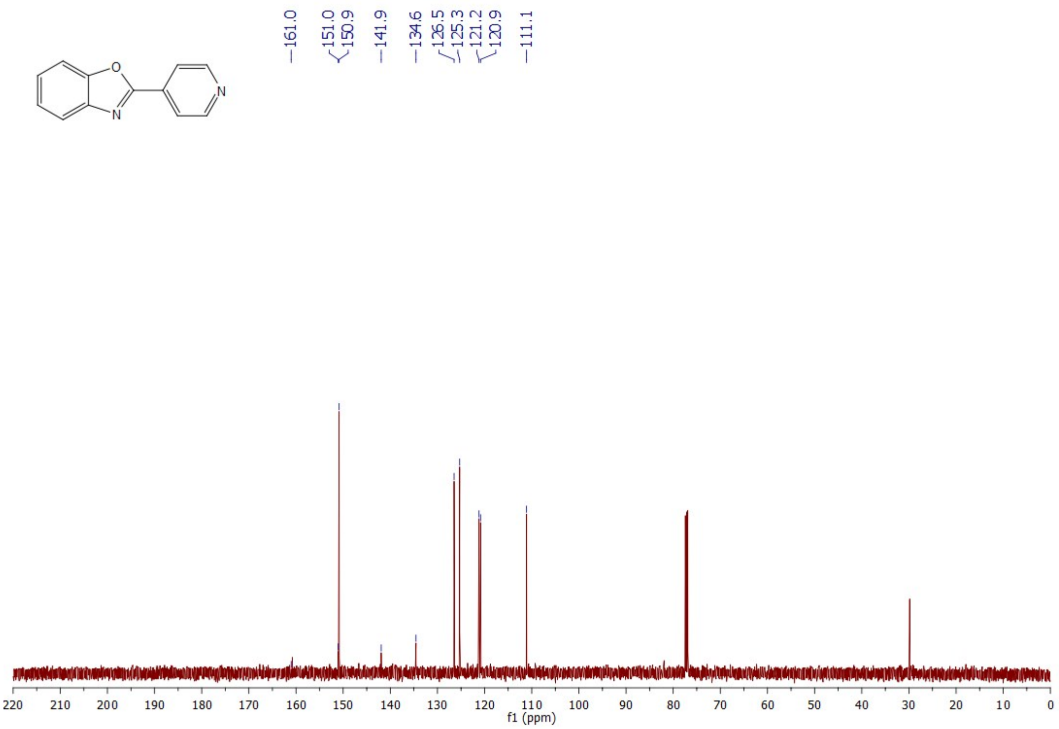

Figure 13. ^{1}H (top) and ^{13}C (bottom) NMR spectra of 2-(Pyridine-4-yl)benzoxazole

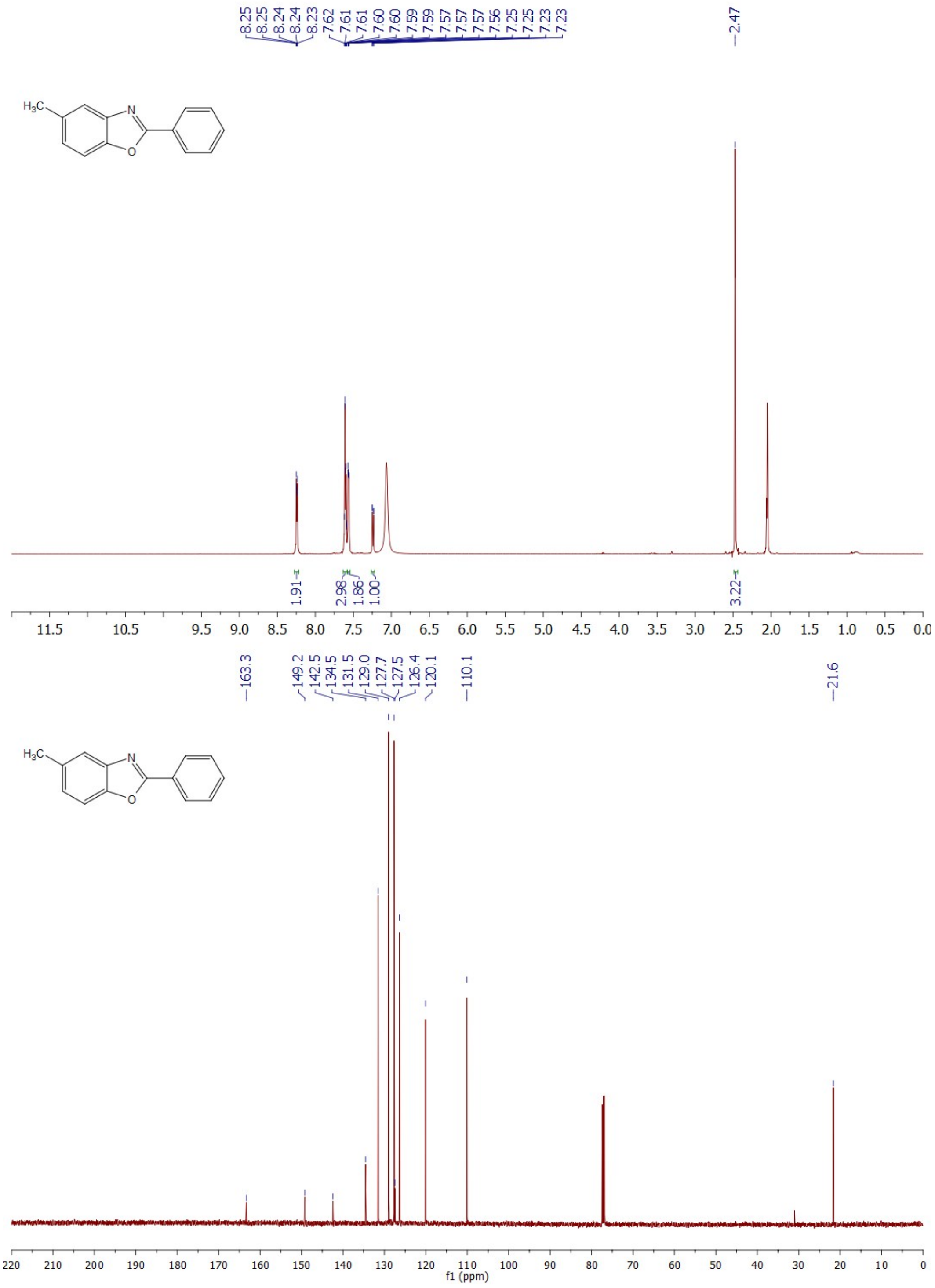

Figure 14. ^{1}H (top) and ^{13}C (bottom) NMR spectra of 5-Methyl-2-phenylbenzoxazole

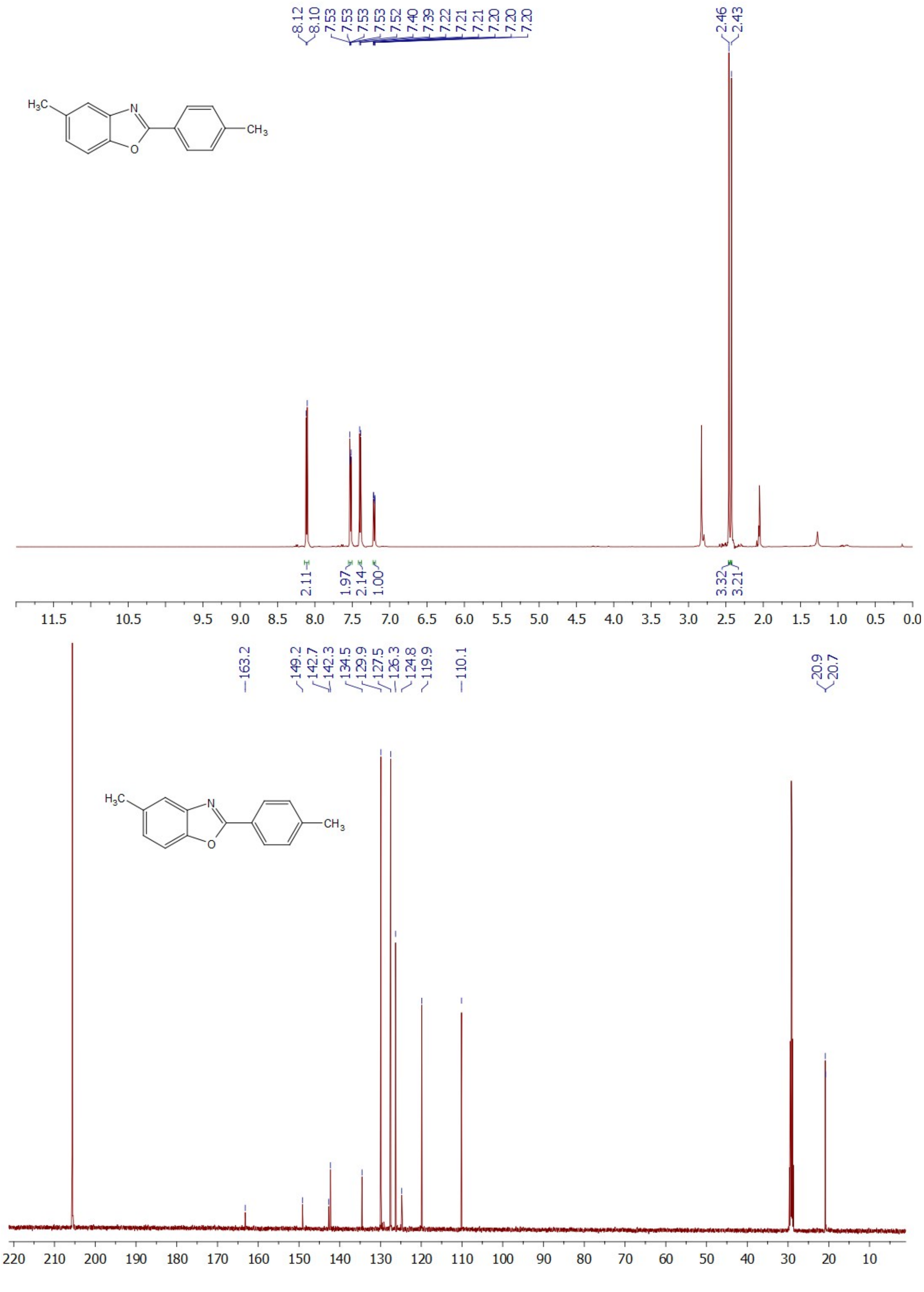

Figure 15. ^{1}H (top) and ^{13}C (bottom) NMR spectra of 5-Methyl-2-(*p*-tolyl)benzoxazole

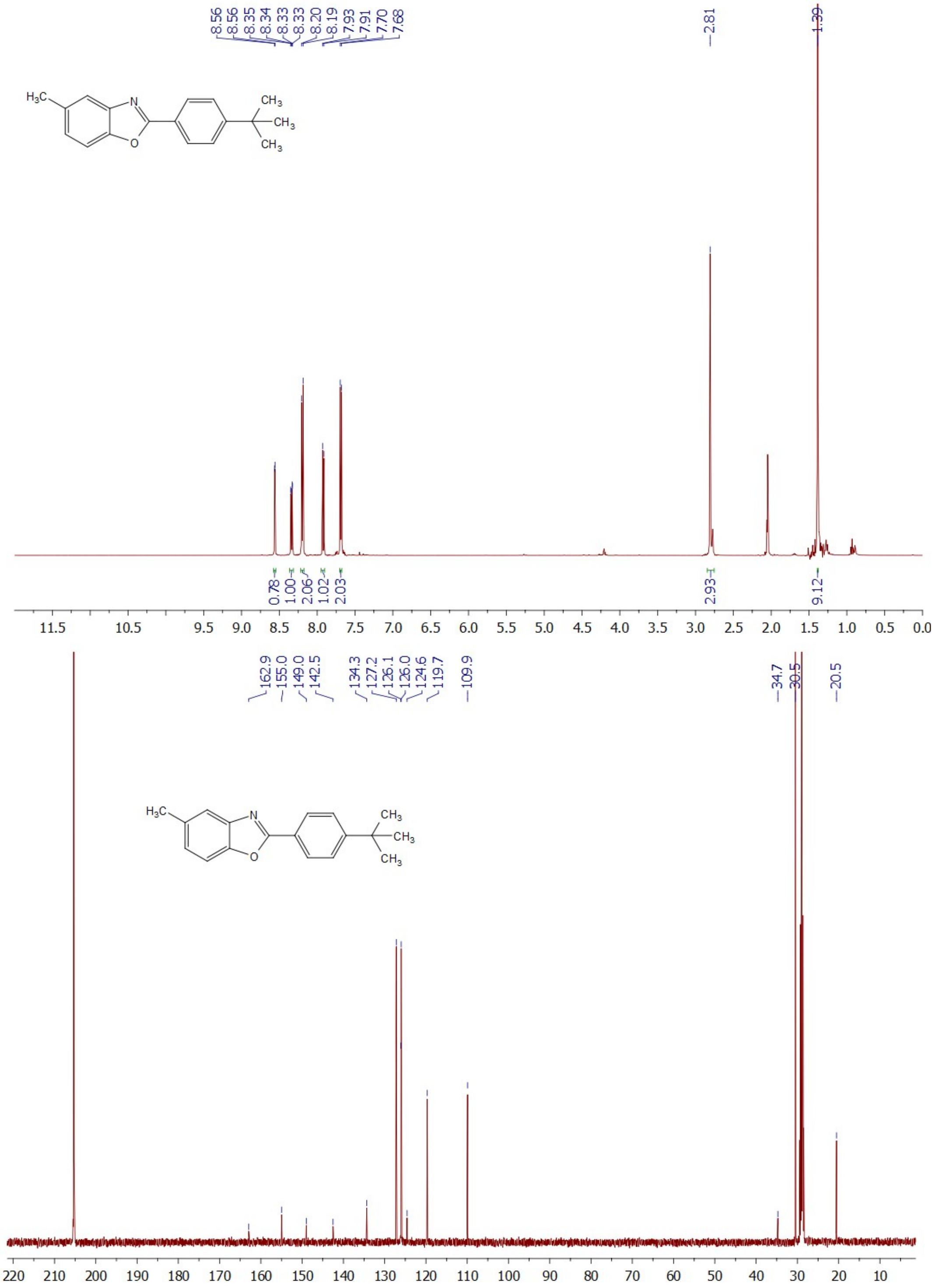

Figure 16. ^{1}H (top) and ^{13}C (bottom) NMR spectra of 5-Methyl-2-(4-*tert*-butylphenyl)benzoxazole

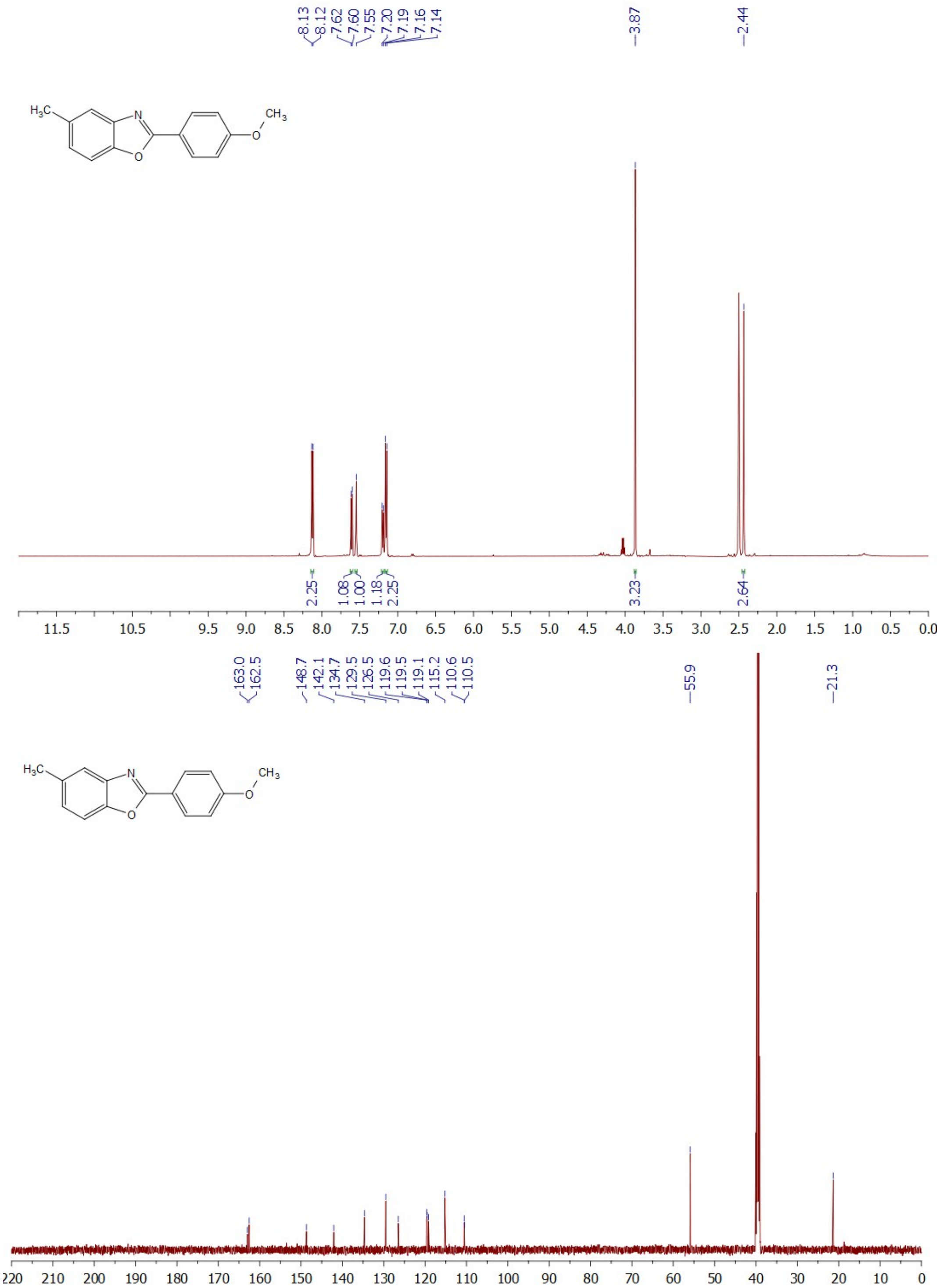

Figure 17. ^{1}H (top) and ^{13}C (bottom) NMR spectra of 5-Methyl-2-(4-methoxyphenyl)benzoxazole

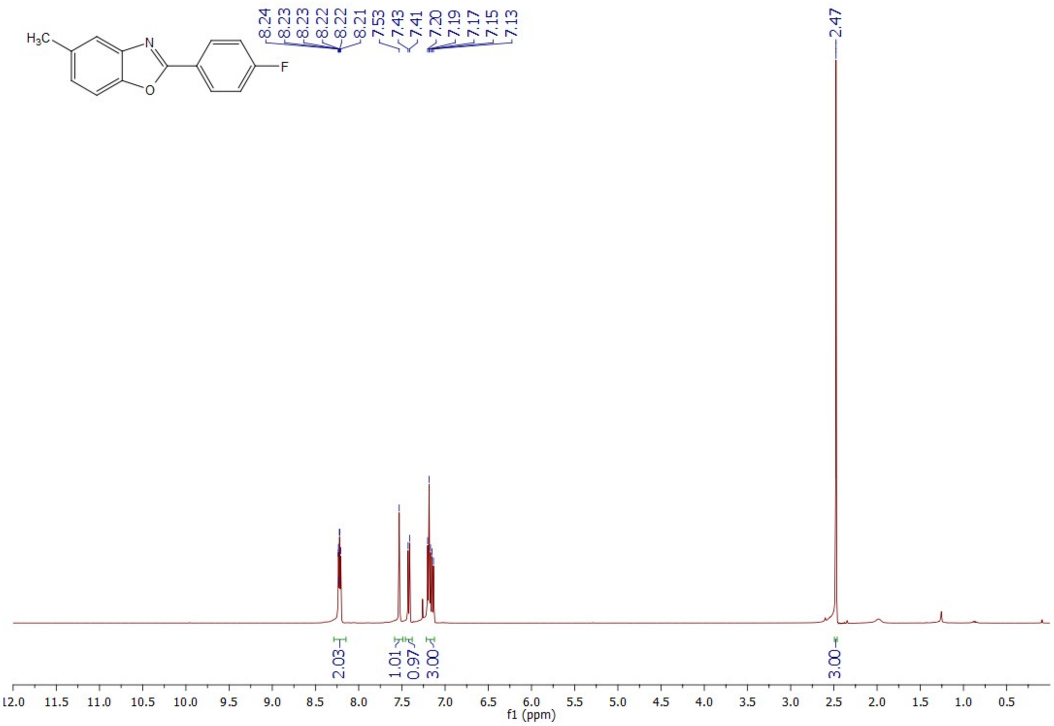

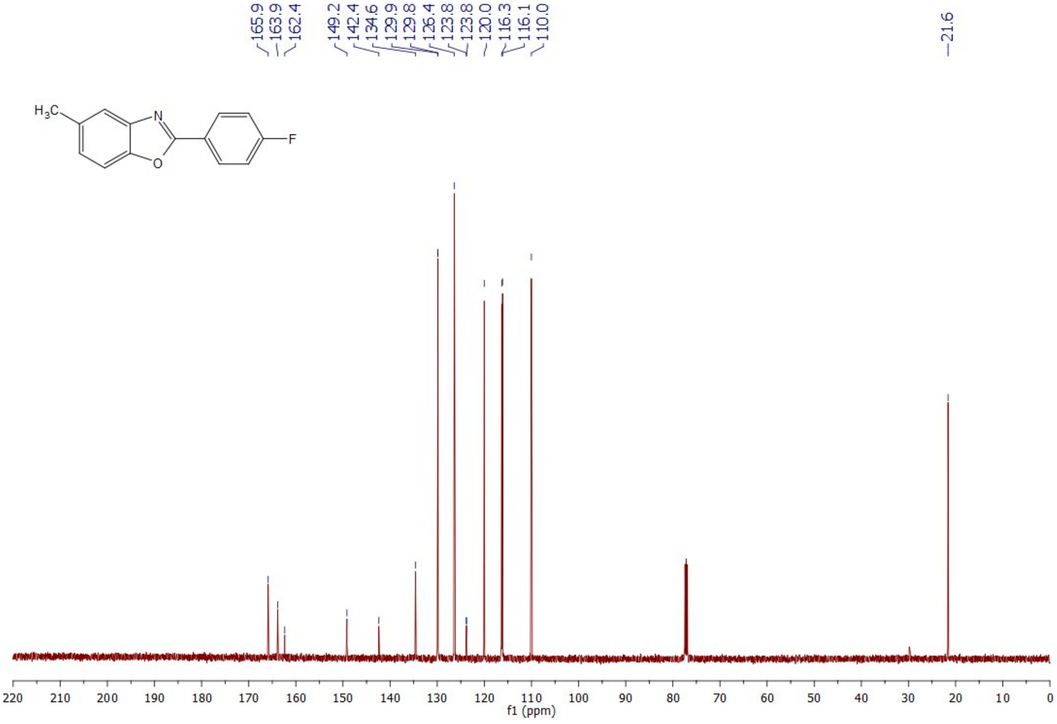

Figure 18. ^{1}H (top) and ^{13}C (bottom) NMR spectra of 5-Methyl-2-(4-fluorophenyl)benzoxazole

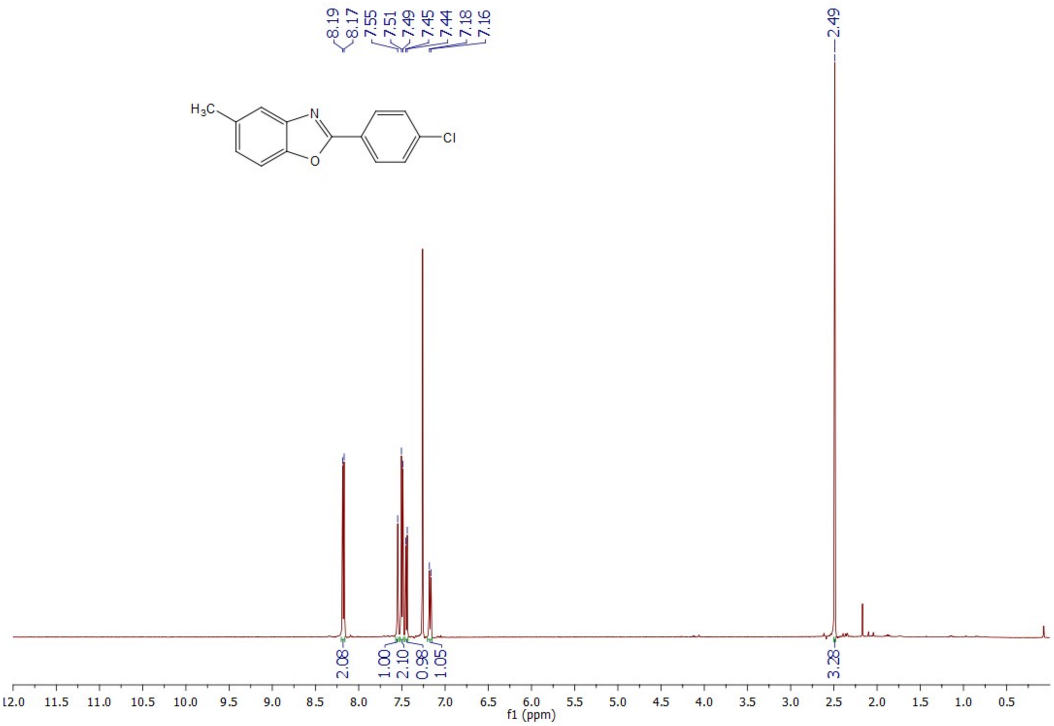

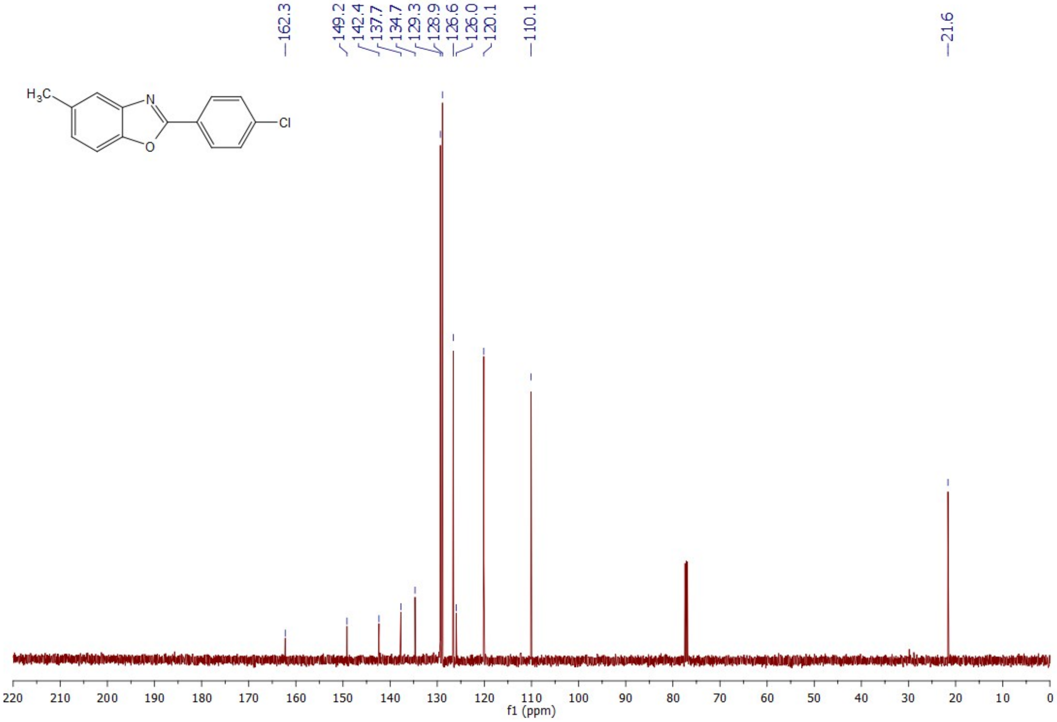

Figure 19. ^1H (top) and ^{13}C (bottom) NMR spectra of 5-Methyl-2-(4-chlorophenyl)benzoxazole

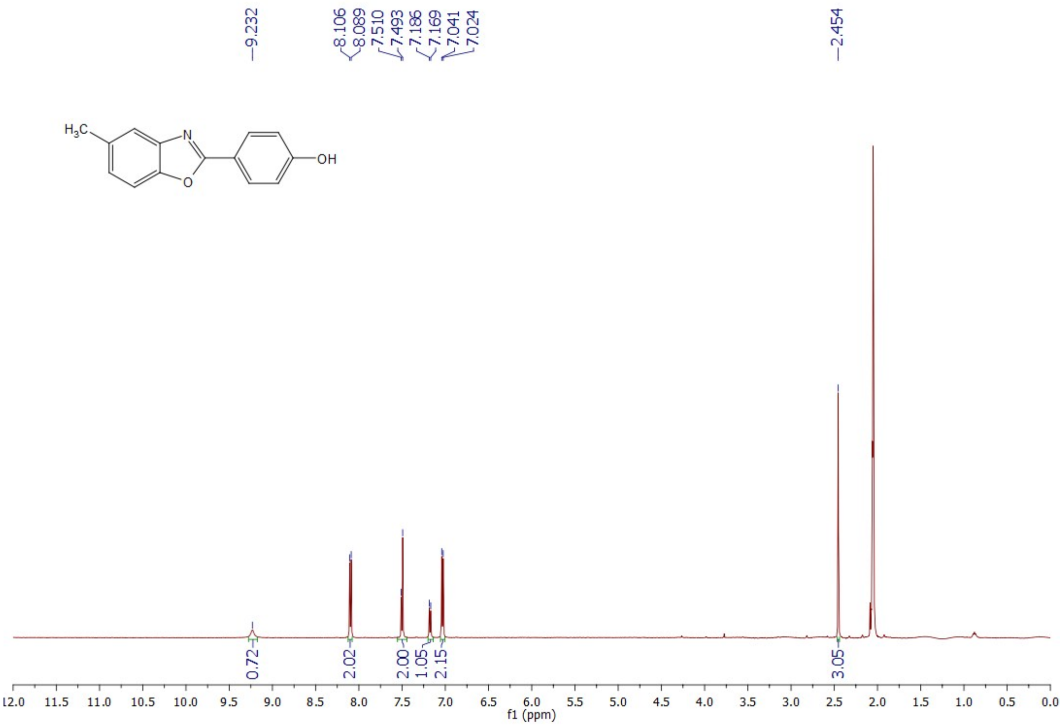

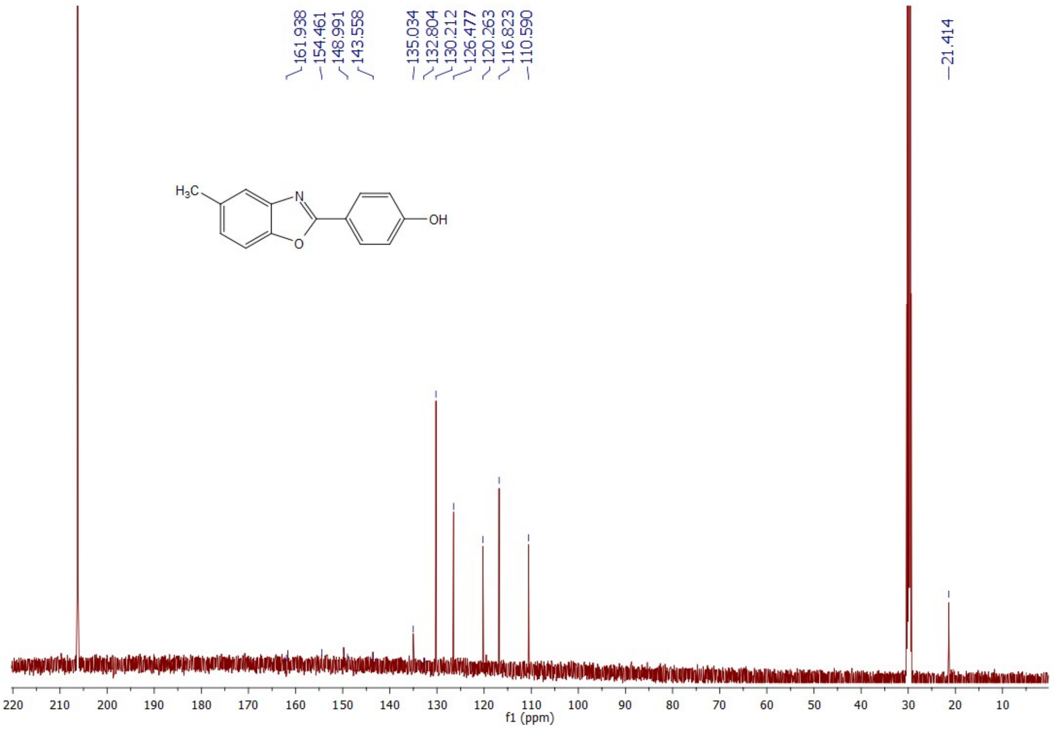

Figure 20. ^{1}H (top) and ^{13}C (bottom) NMR spectra of 5-Methyl-2-(4-hydroxyphenyl)benzoxazole

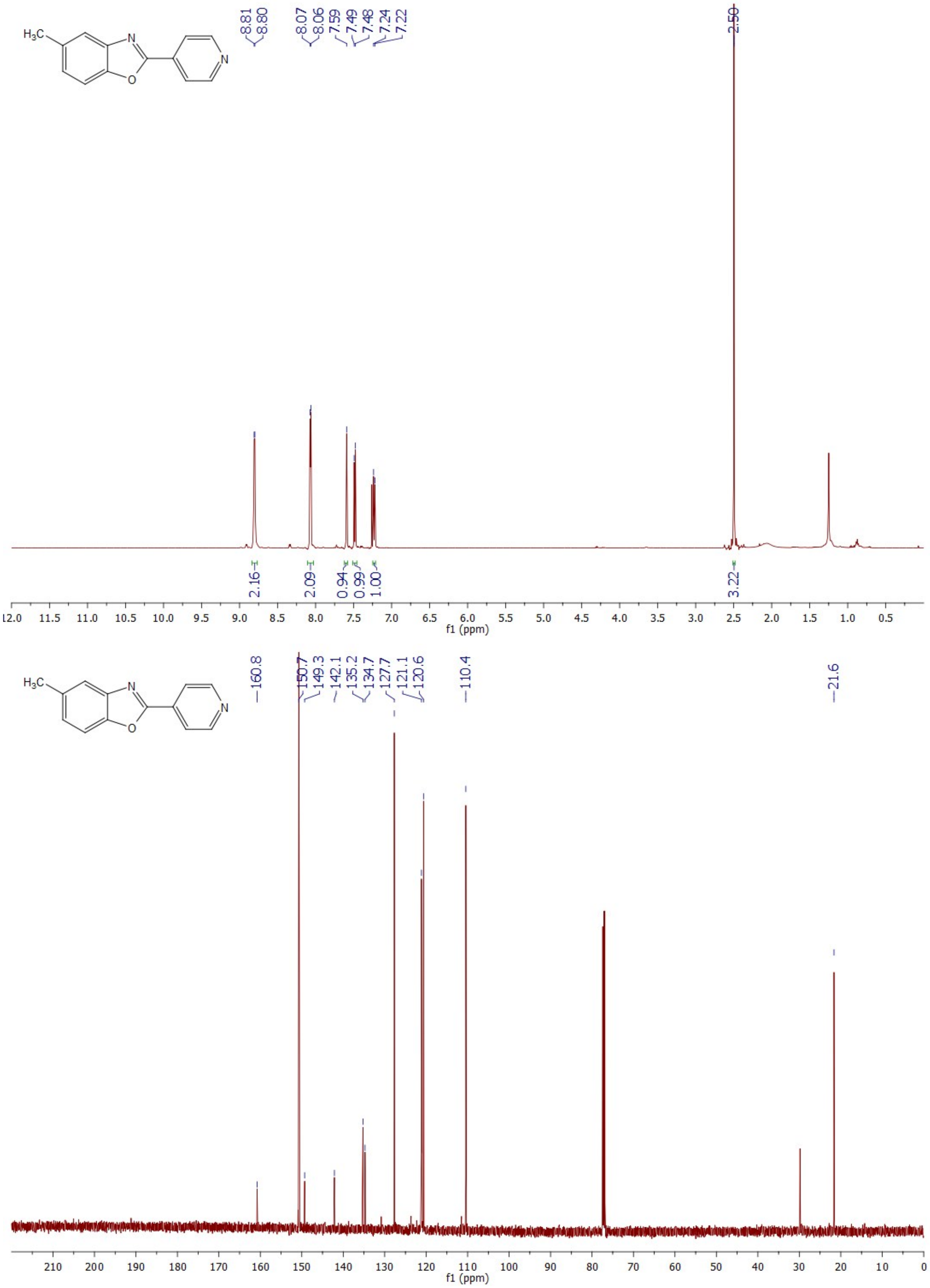

Figure 21. ^{1}H (top) and ^{13}C (bottom) NMR spectra of 5-Methyl-2-(pyridin-4-yl)benzoxazole

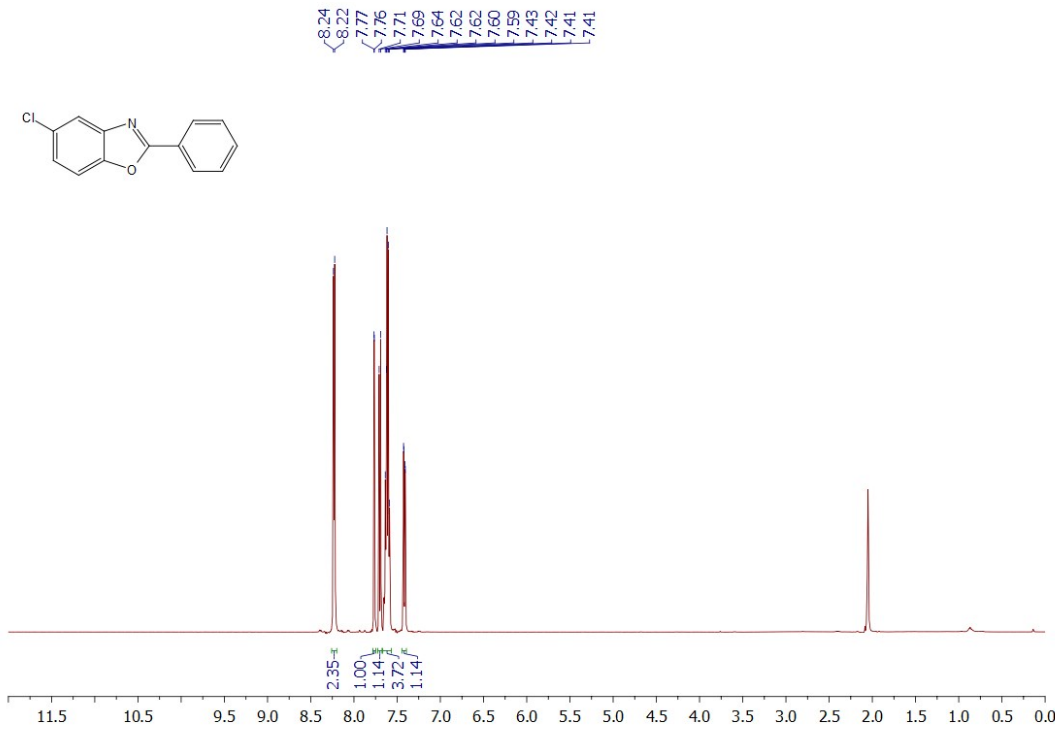

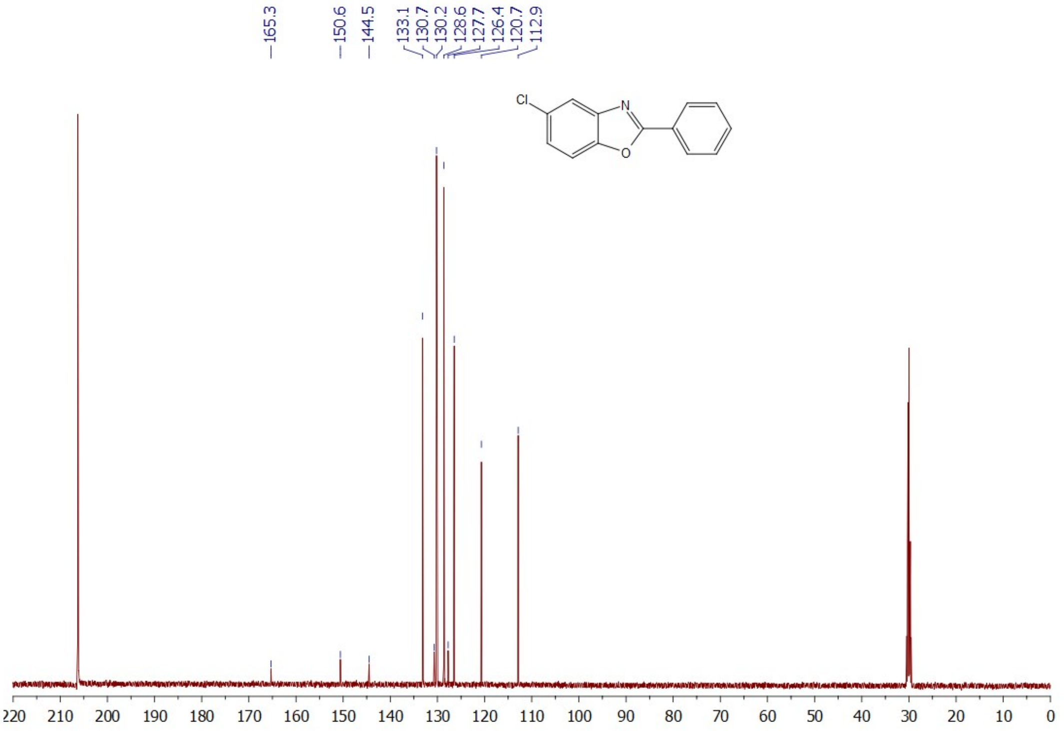

Figure 22. ^{1}H (top) and ^{13}C (bottom) NMR spectra of 5-Chloro-2-phenylbenzoxazole

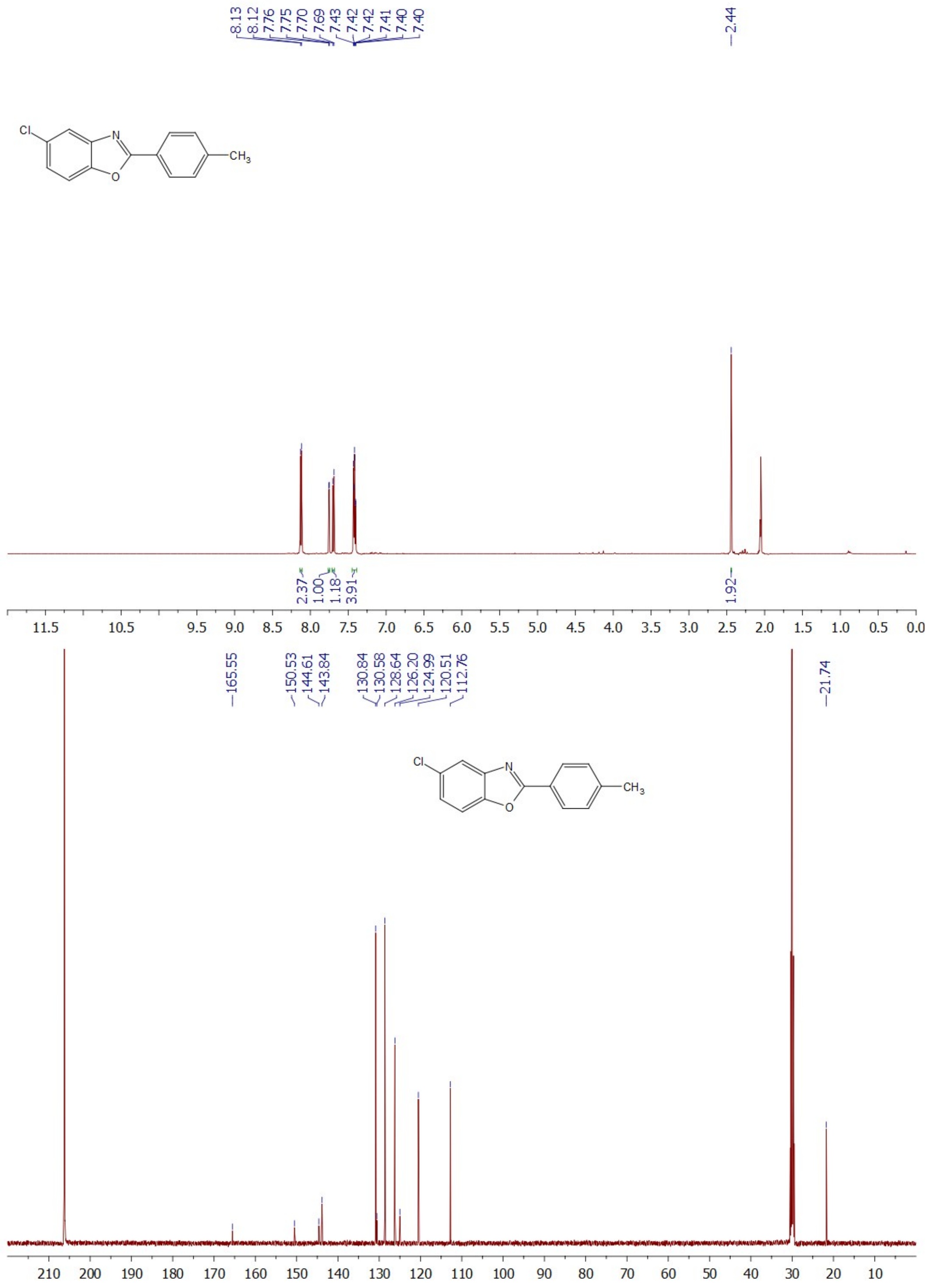

Figure 23. ^{1}H (top) and ^{13}C (bottom) NMR spectra of 5-Chloro-2-(*p*-tolyl)benzoxazole

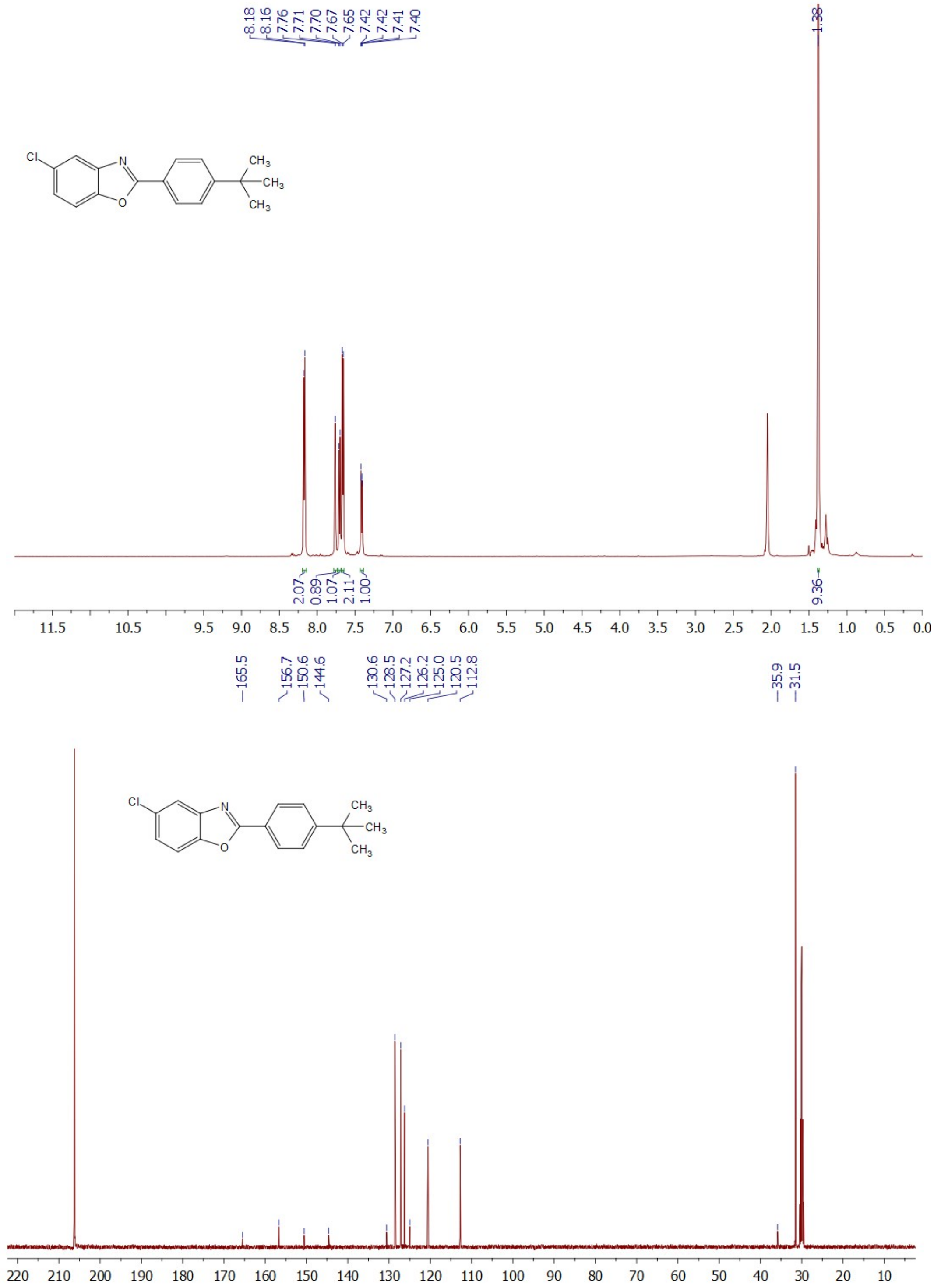

Figure 24. ^{1}H (top) and ^{13}C (bottom) NMR spectra of 5-Chloro-2-(4-*tert*-butylphenyl)benzoxazole

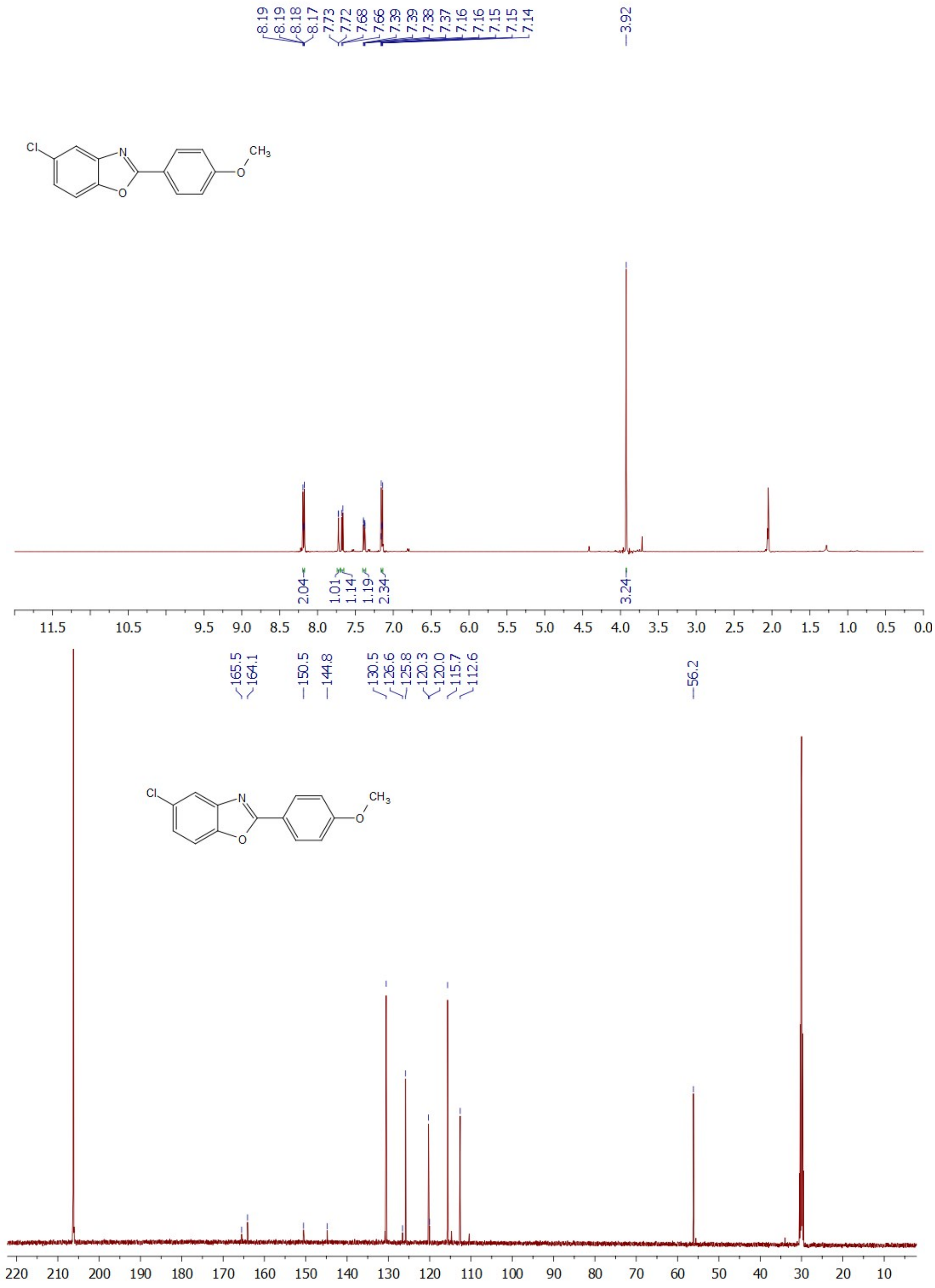

Figure 25. ^{1}H (top) and ^{13}C (bottom) NMR spectra of 5-Chloro-2-(4-methoxyphenyl)benzoxazole

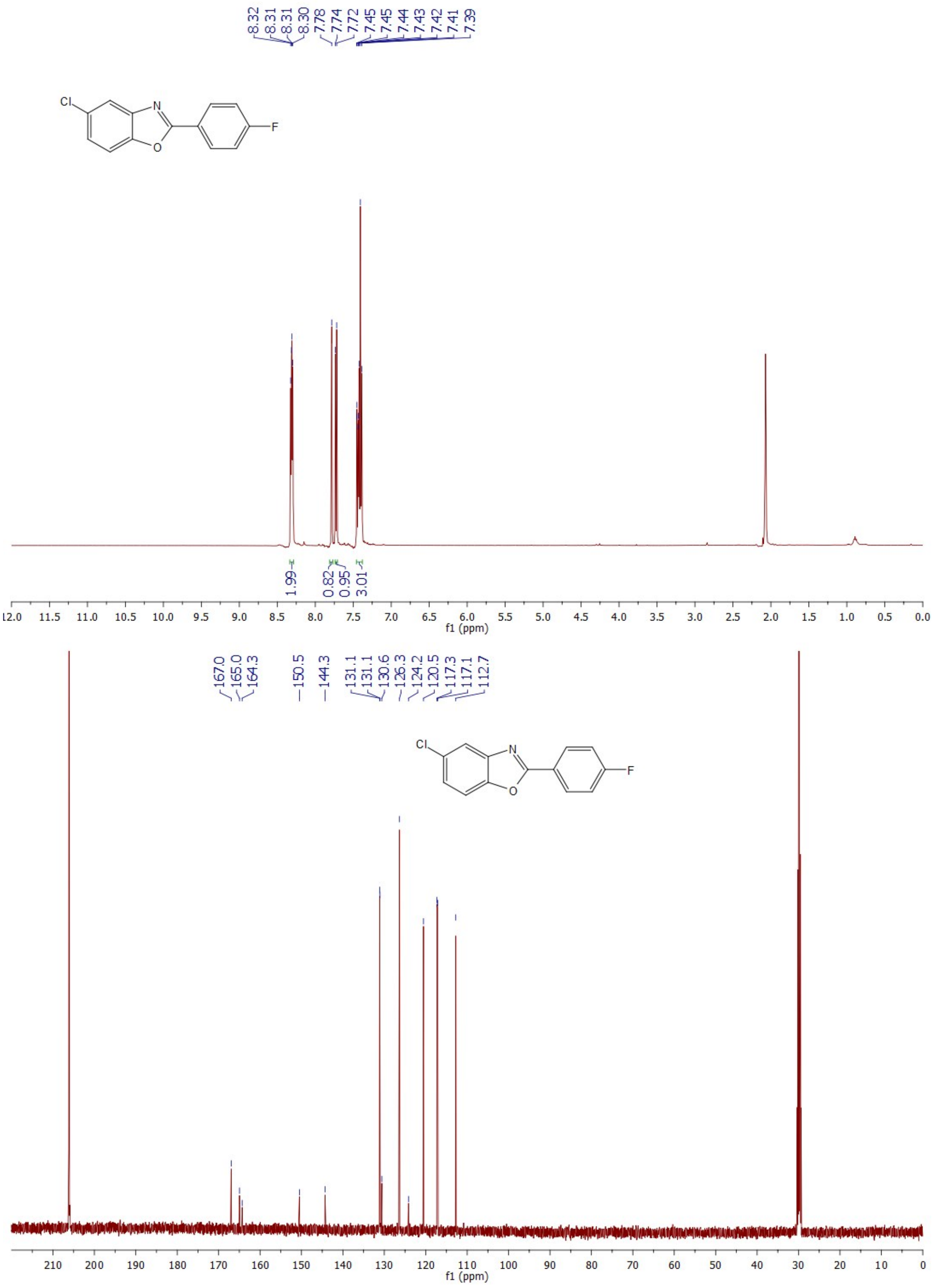

Figure 26. ^{1}H (top) and ^{13}C (bottom) NMR spectra of 5-chloro-2-(4-Fluorophenyl)benzoxazole

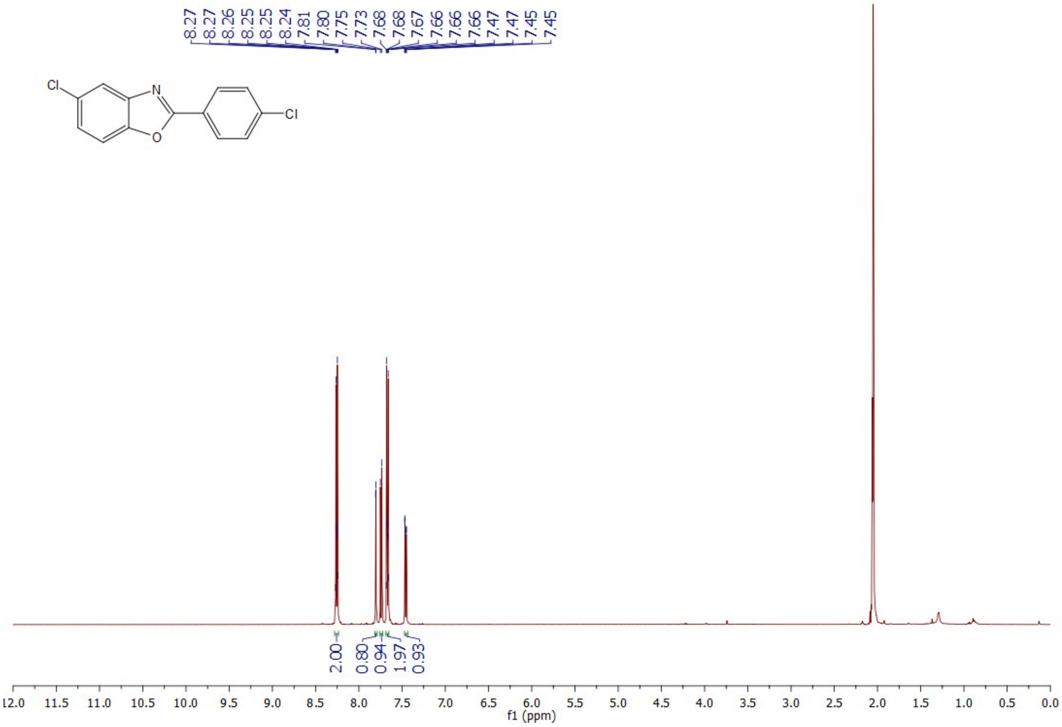

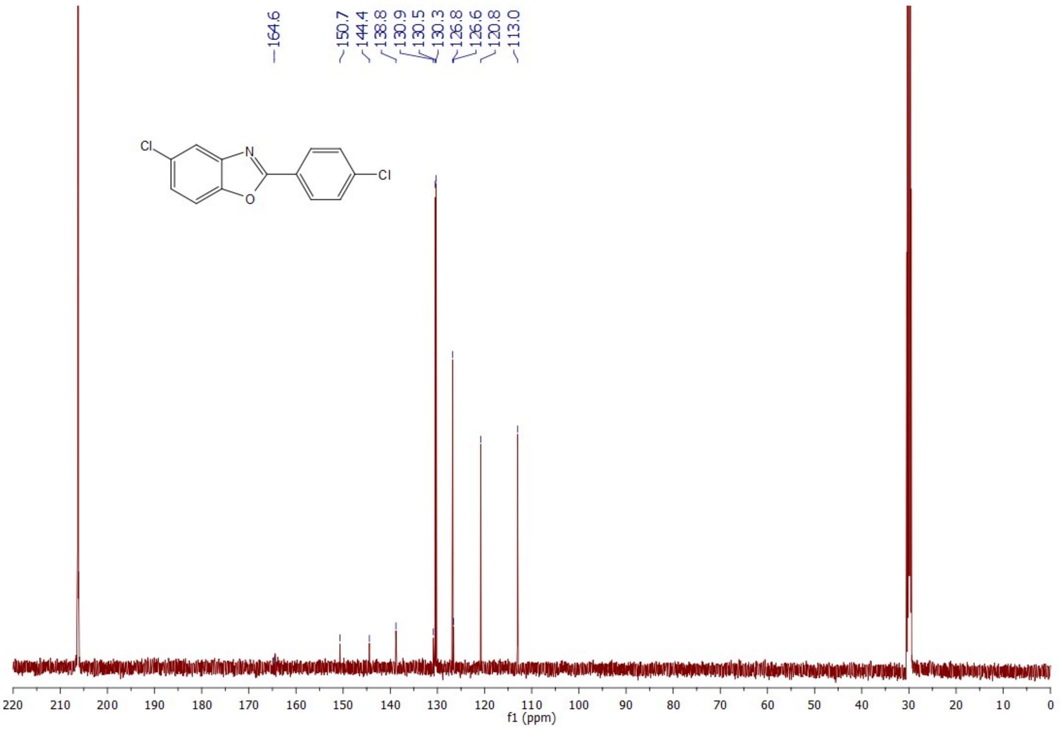

Figure 27. ^{1}H (top) and ^{13}C (bottom) NMR spectra 5-Chloro-2-(4-chlorophenyl)benzoxazole

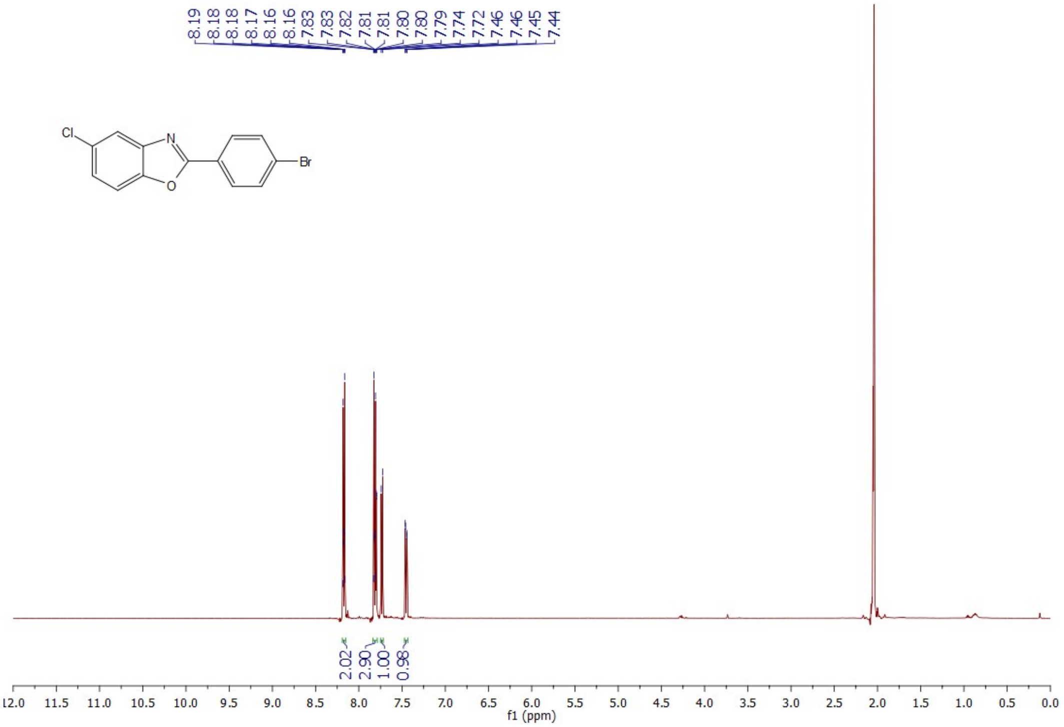

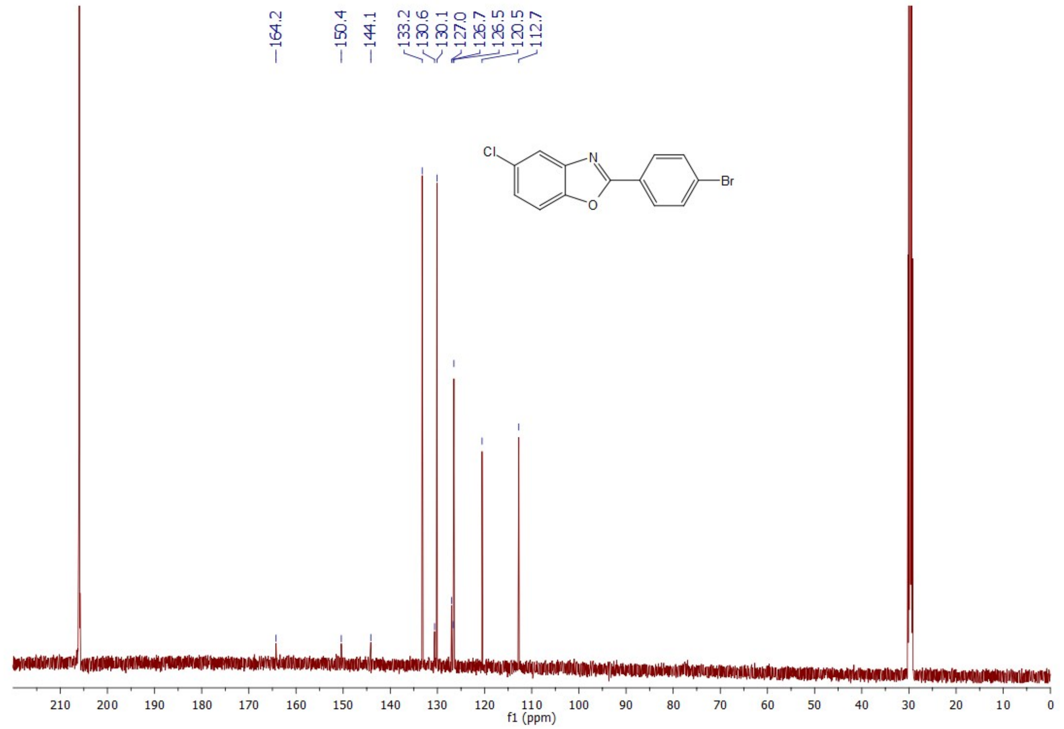

Figure 28. ^{1}H (top) and ^{13}C (bottom) NMR spectra of 5-Chloro-2-(4-bromophenyl)benzoxazole

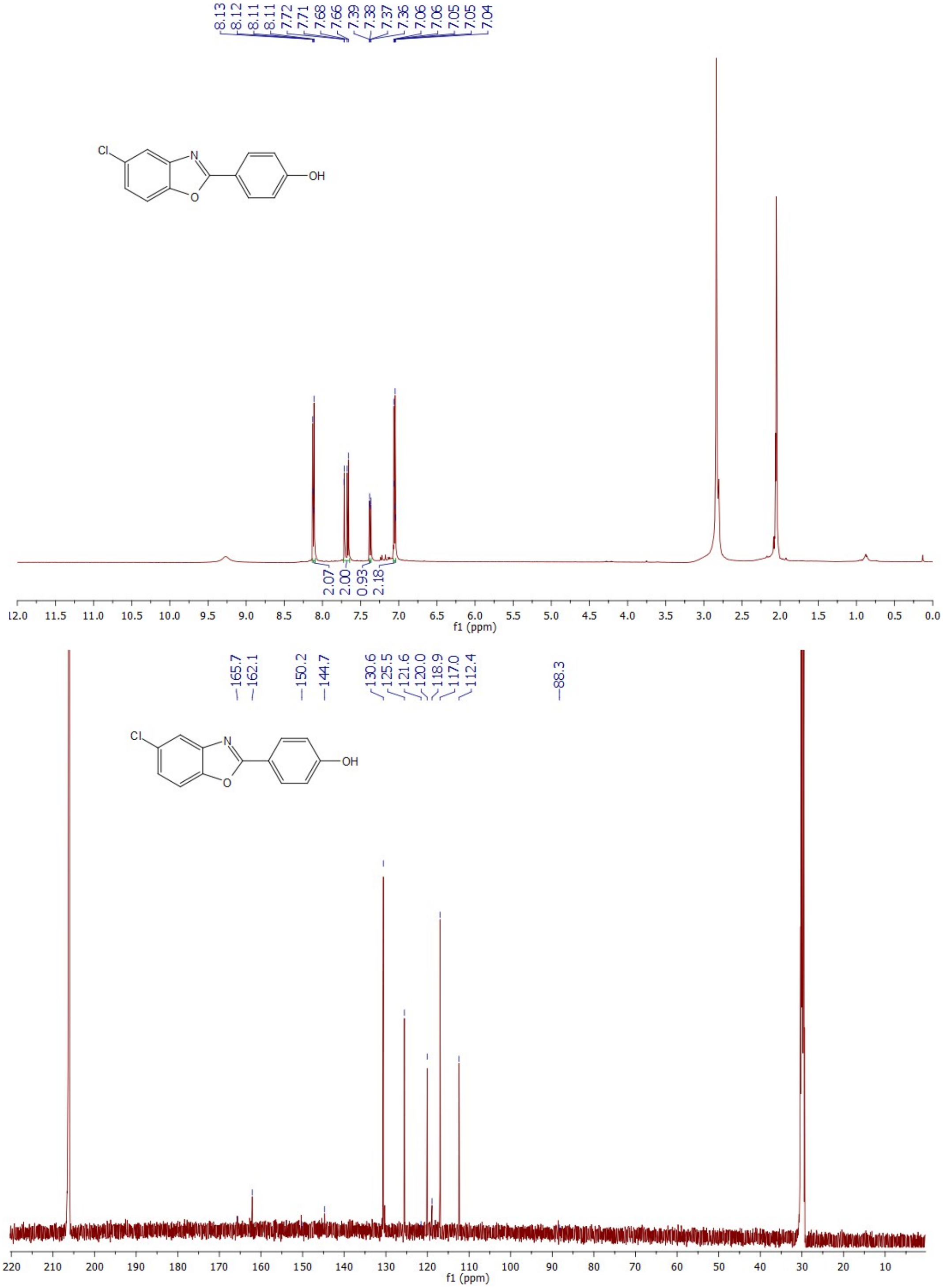

Figure 29. ^{1}H (top) and ^{13}C (bottom) NMR spectra of 5-chloro-2-(4-hydroxyphenyl)benzoxazole

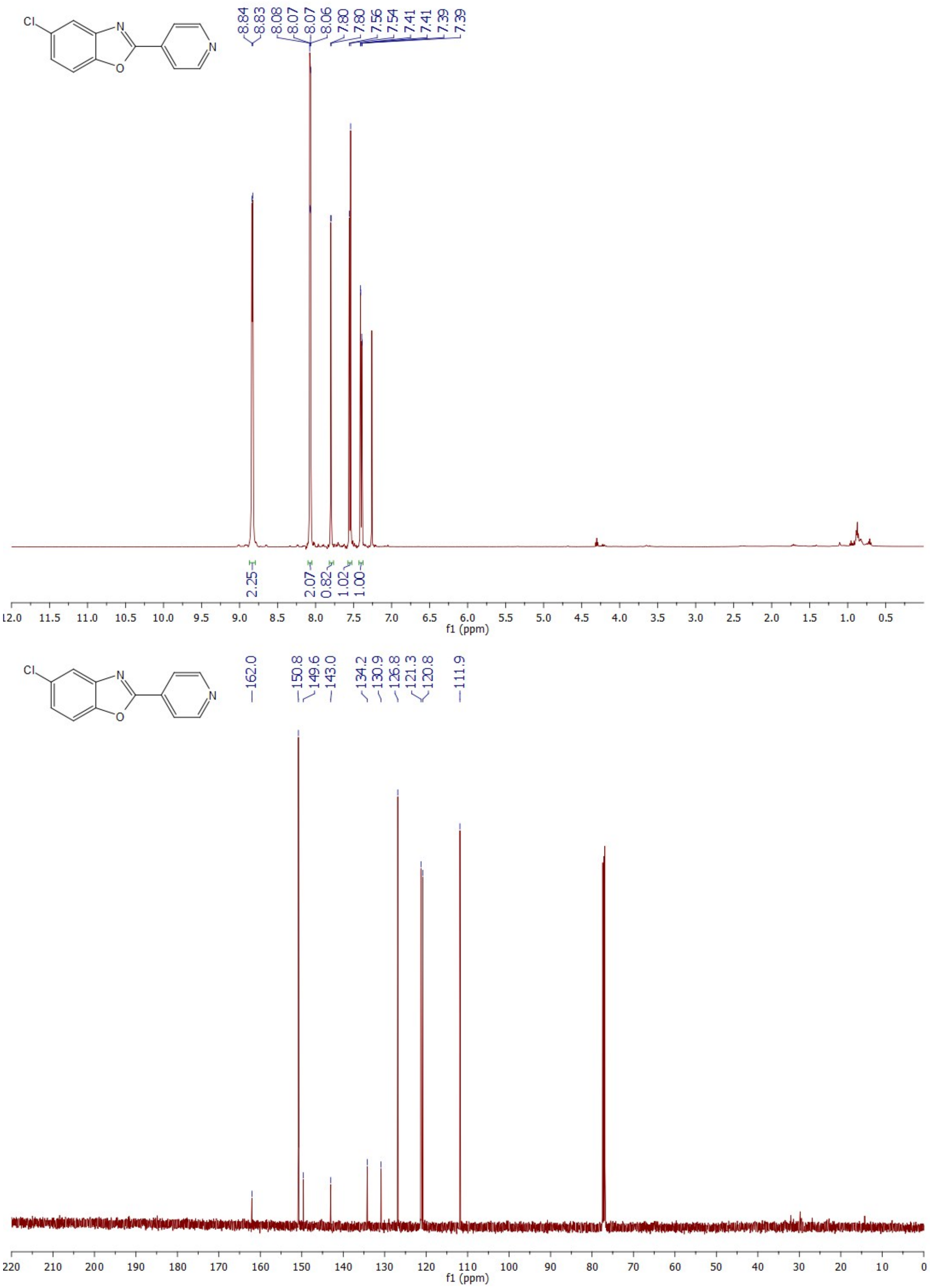

Figure 30. ^{1}H (top) and ^{13}C (bottom) NMR spectra of 5-Chloro-2-(pyridin-4-yl)benzoxazole

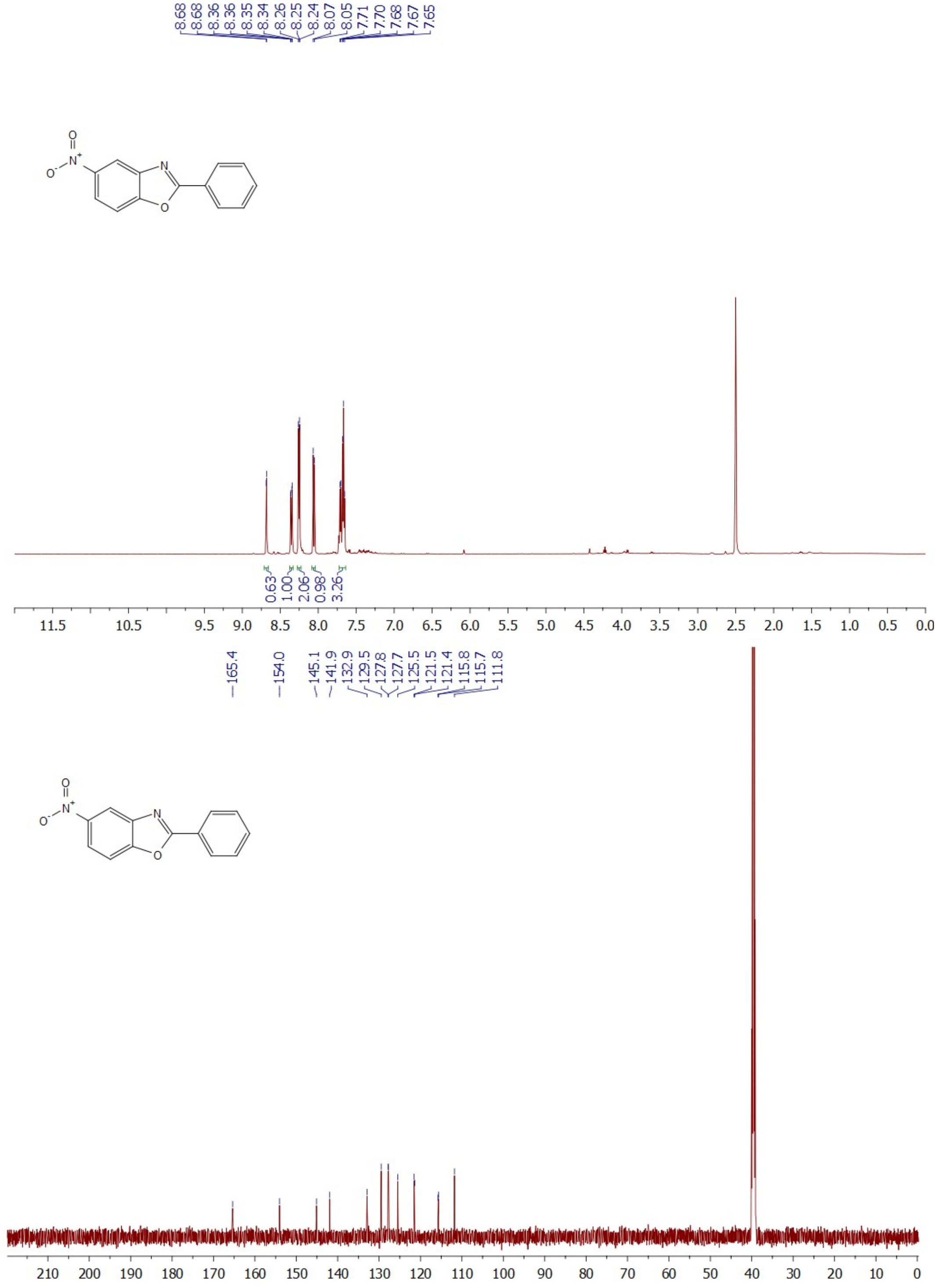

Figure 31. ^{1}H (top) and ^{13}C (bottom) NMR spectra of 5-nitro-2-phenylbenzoxazole

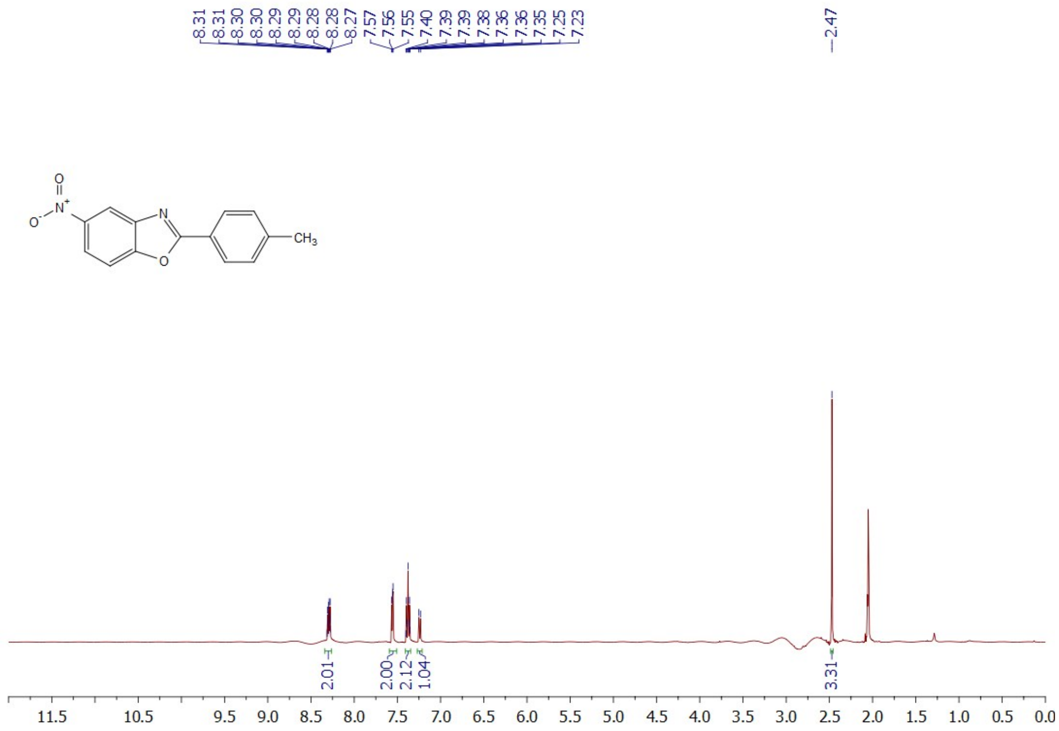

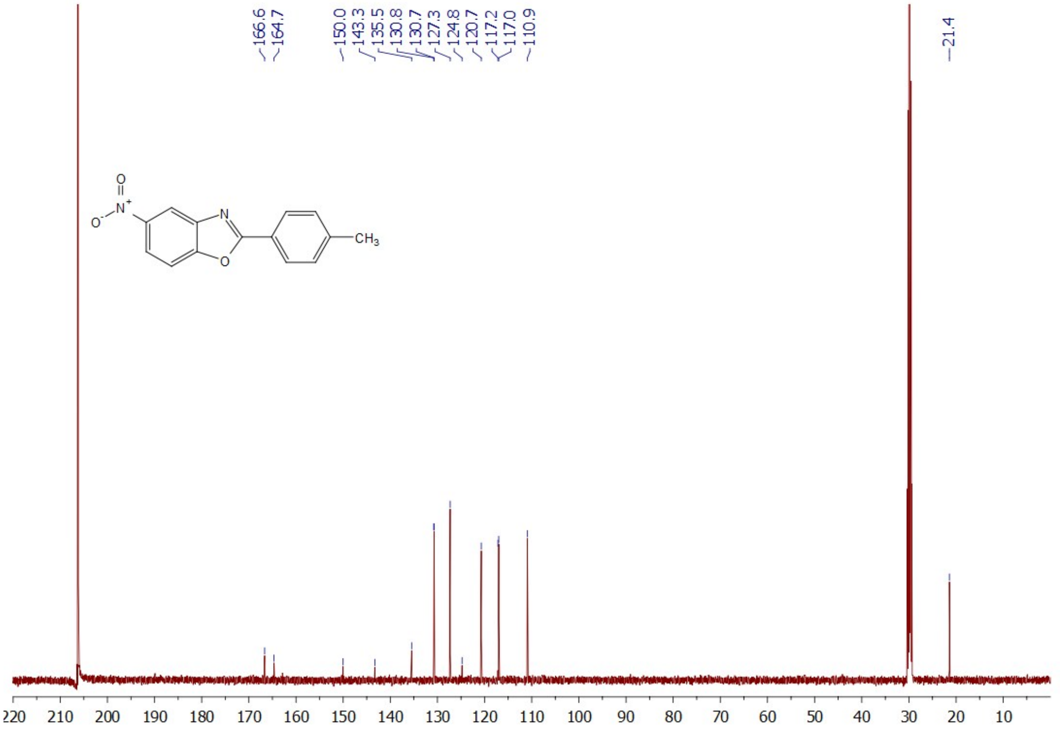

Figure 32. ^{1}H (top) and ^{13}C (bottom) NMR spectra of 5-Nitro-2-(4-methylphenyl)benzoxazole

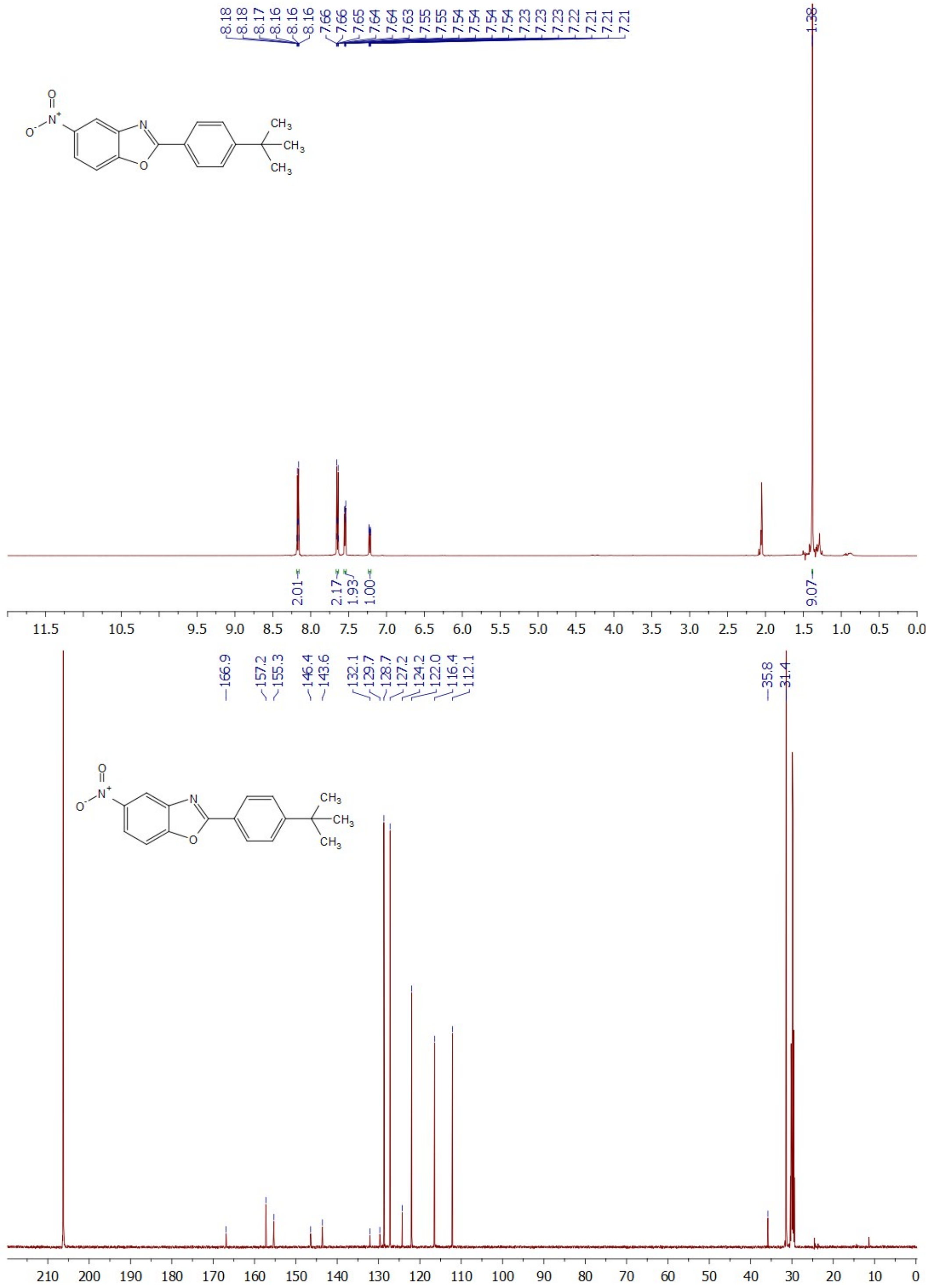

Figure 33. ^{1}H (top) and ^{13}C (bottom) NMR spectra of 5-Nitro-2-(4-*tert*-butylphenyl)benzoxazole

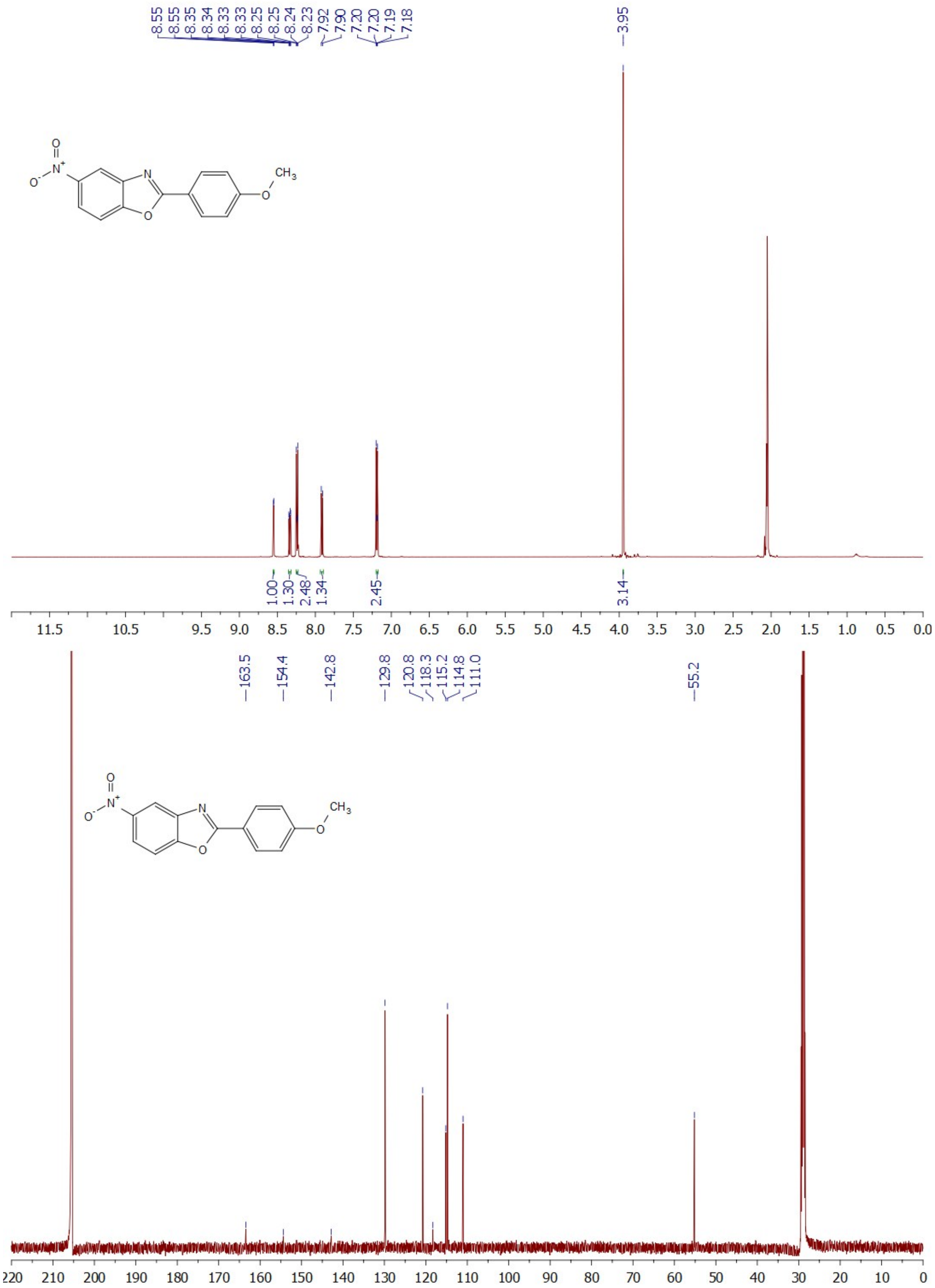

Figure 34. ^{1}H (top) and ^{13}C (bottom) NMR spectra of 5-Nitro-2-(4-methoxyphenyl)benzoxazole

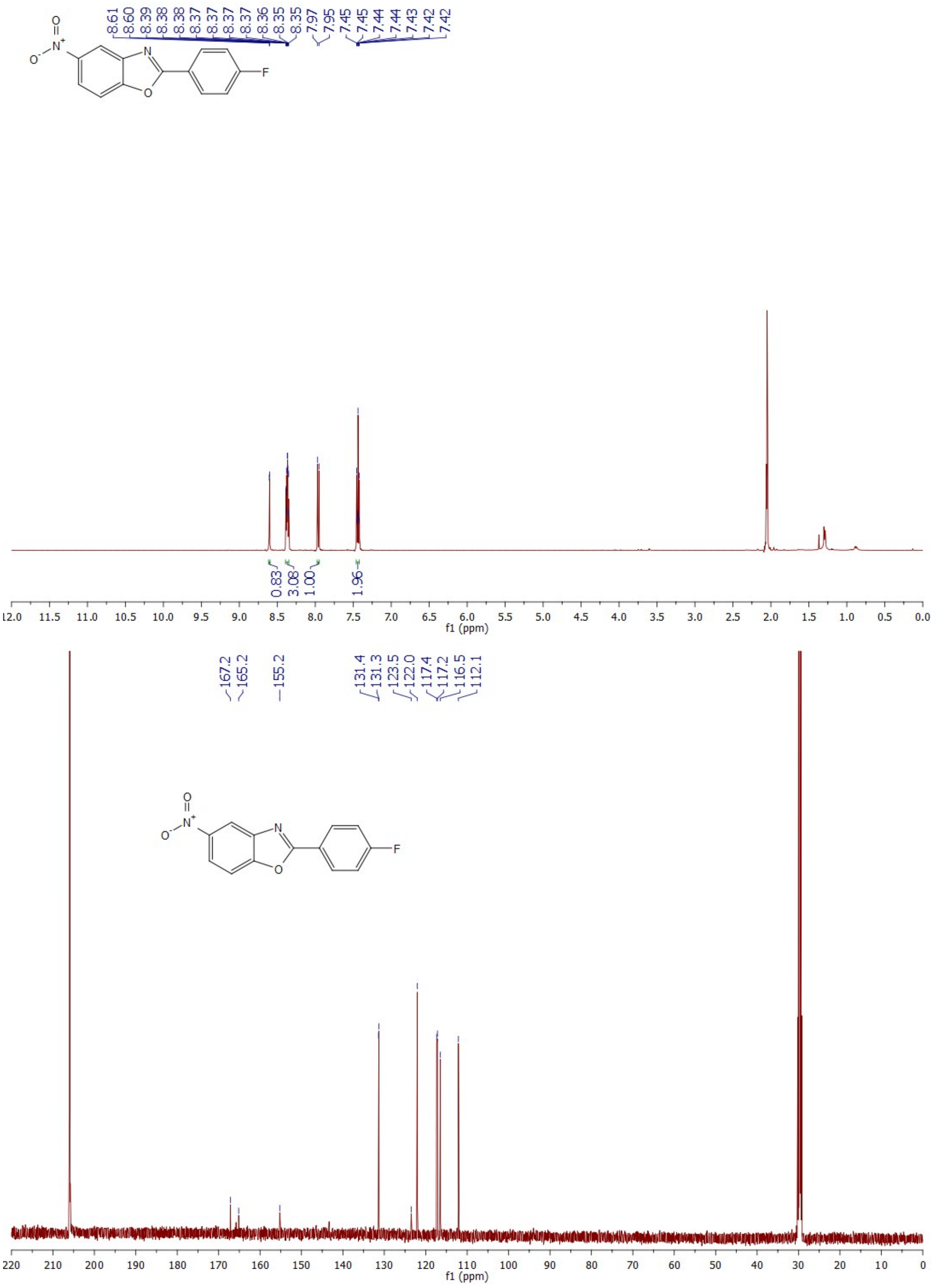

Figure 35. ^{1}H (top) and ^{13}C (bottom) NMR spectra of 5-Nitro-2-(4-fluorophenyl)benzoxazole

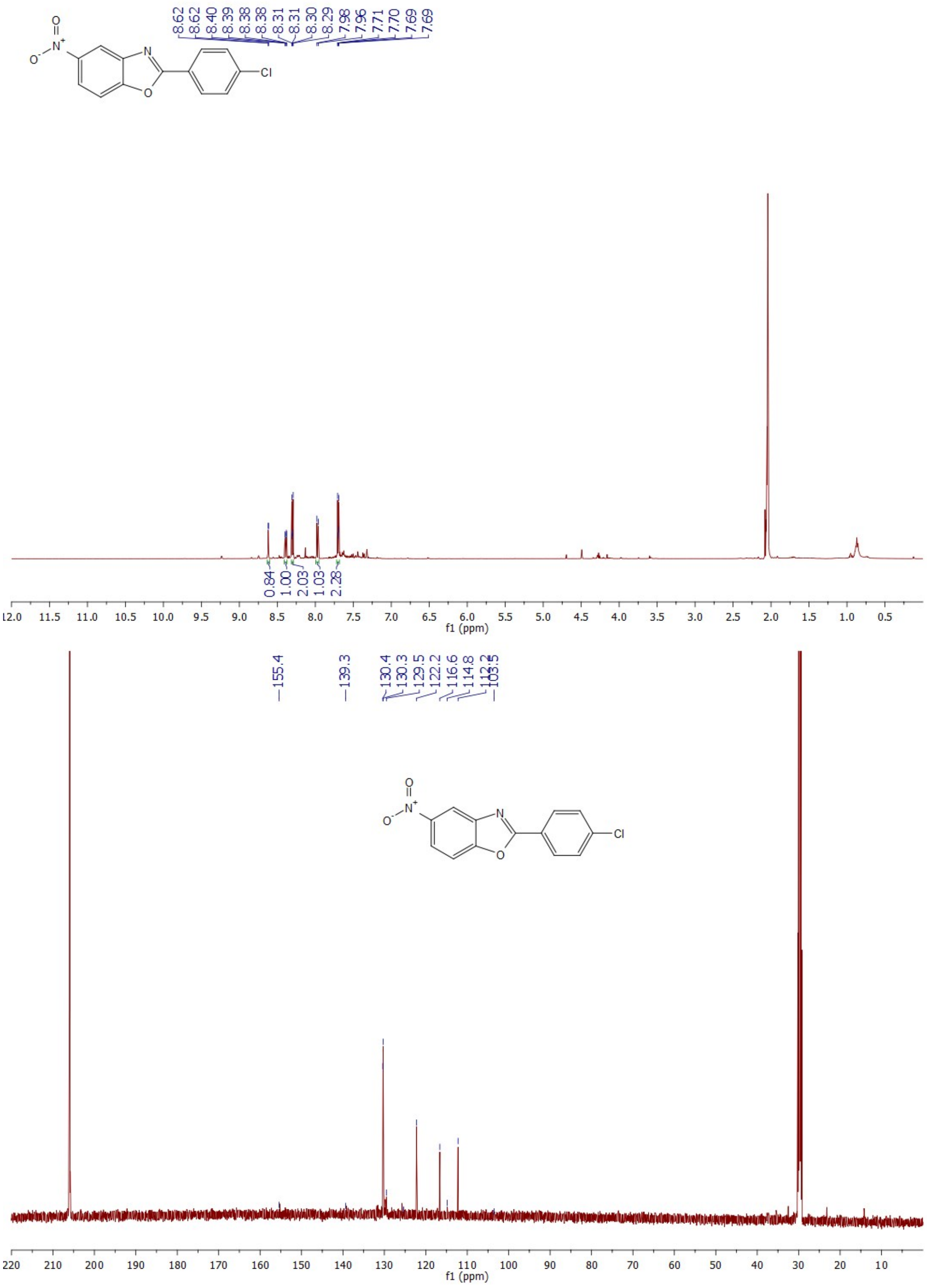

Figure 36. ^{1}H (top) and ^{13}C (bottom) NMR spectra of 5-Nitro-2-(4-chlorophenyl)benzoxazole

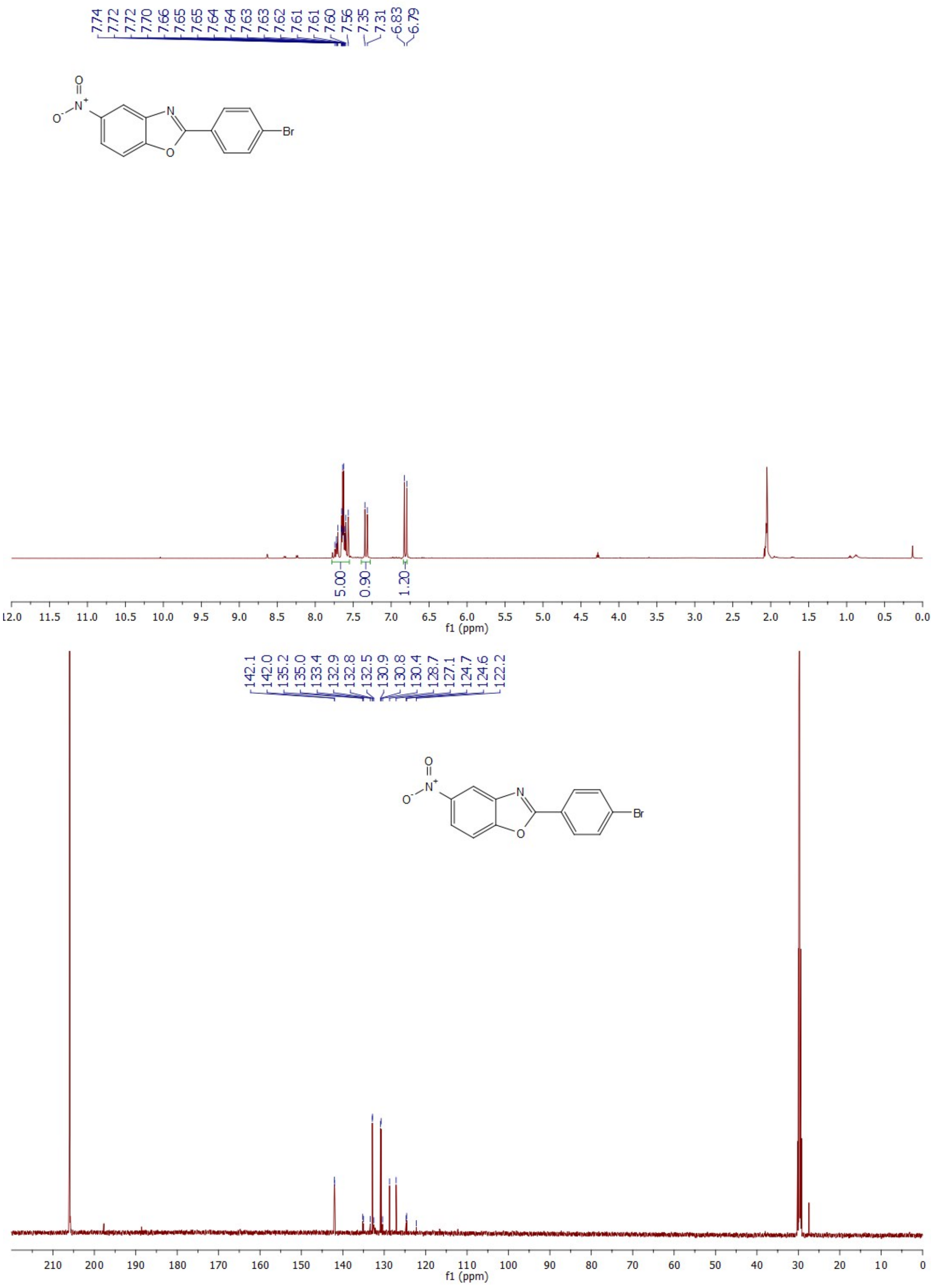

Figure 37. ^{1}H (top) and ^{13}C (bottom) NMR spectra of 5-Nitro-2-(4-bromophenyl)benzoxazole

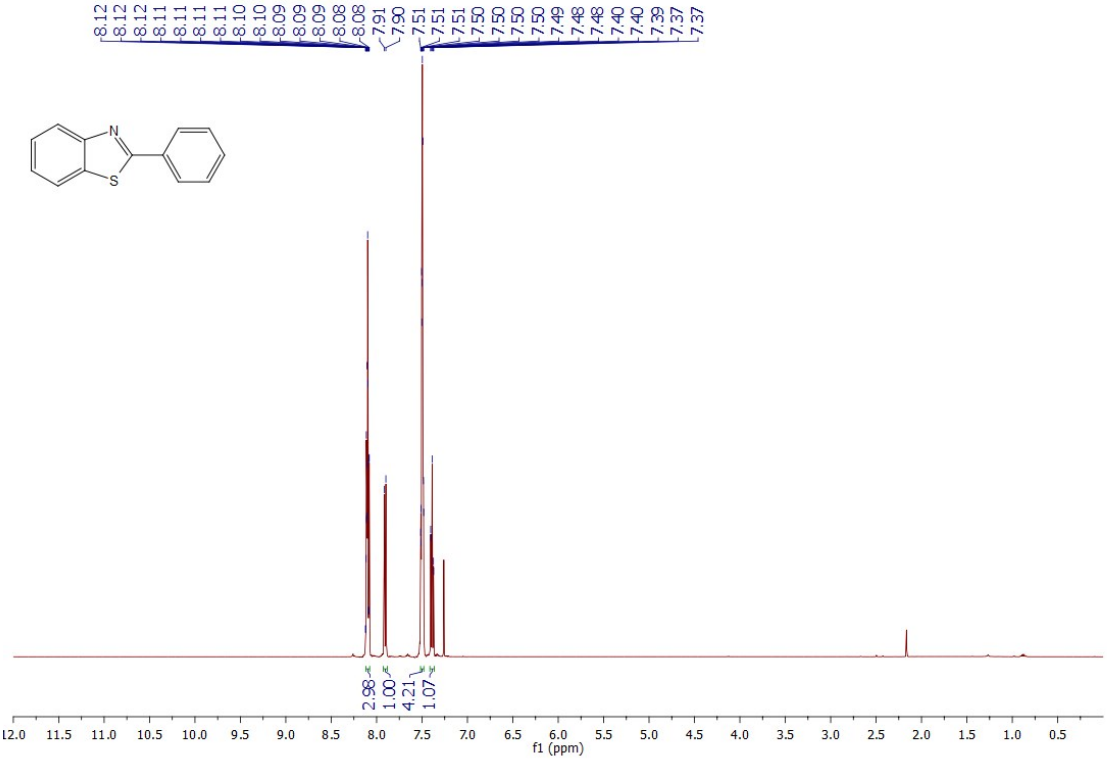

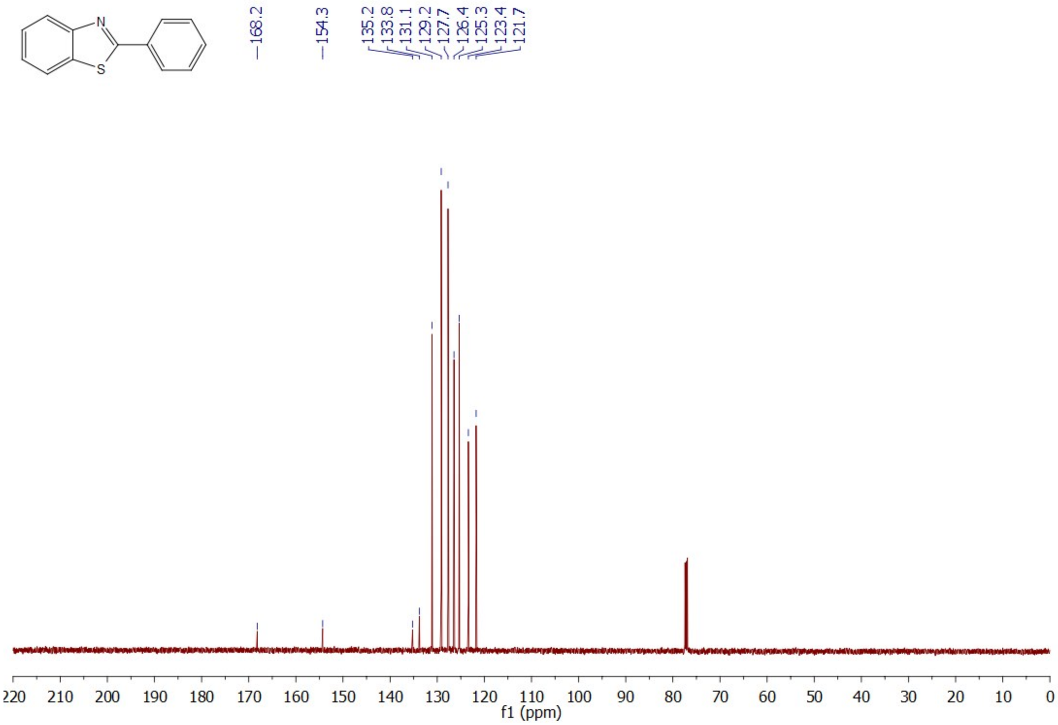

Figure 38. ^{1}H (top) and ^{13}C (bottom) NMR spectra of 2-Phenylbenzothiazole

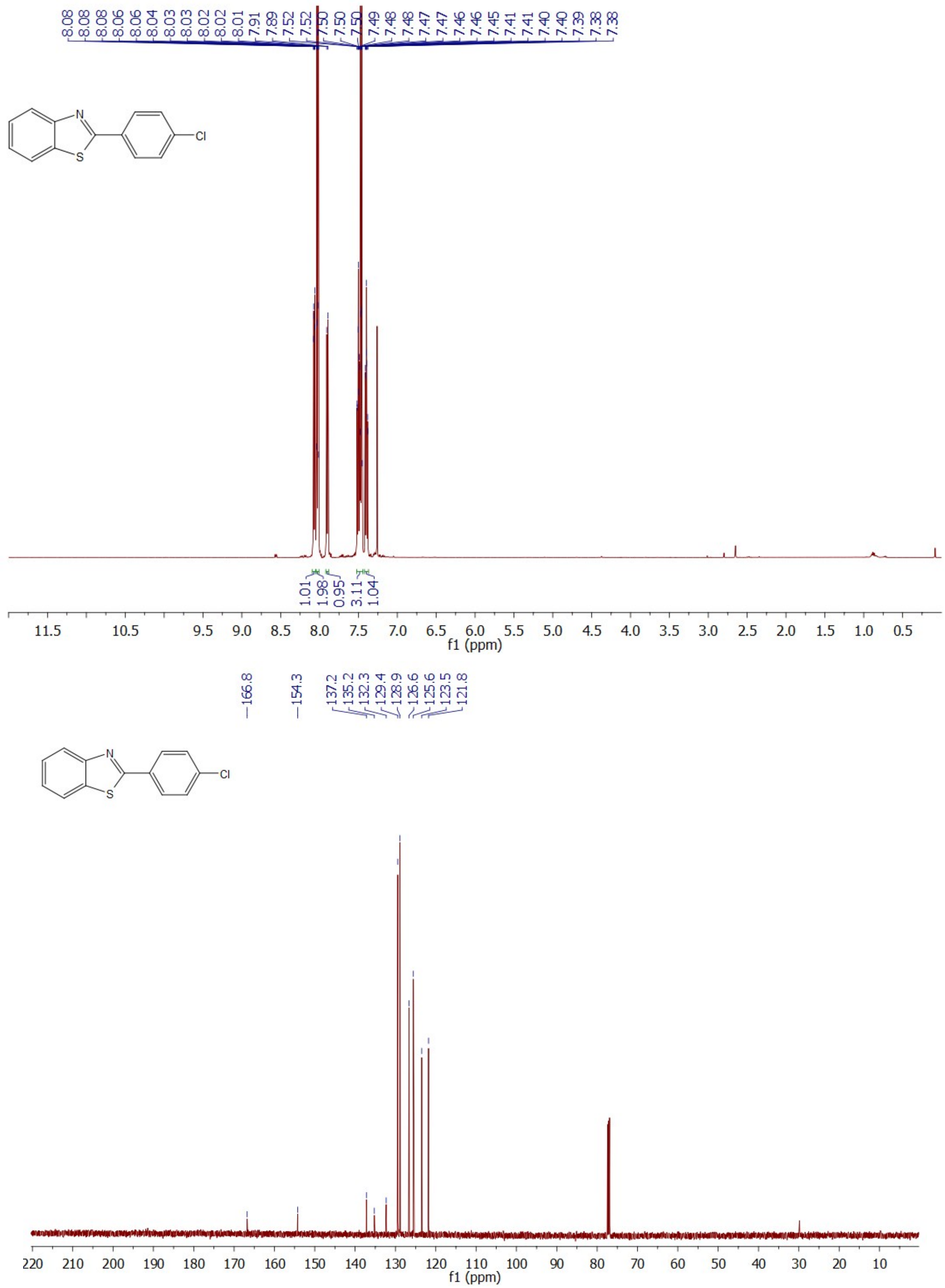

Figure 39. ^{1}H (top) and ^{13}C (bottom) NMR spectra of 2-(4-Chlorophenyl)benzothiazole

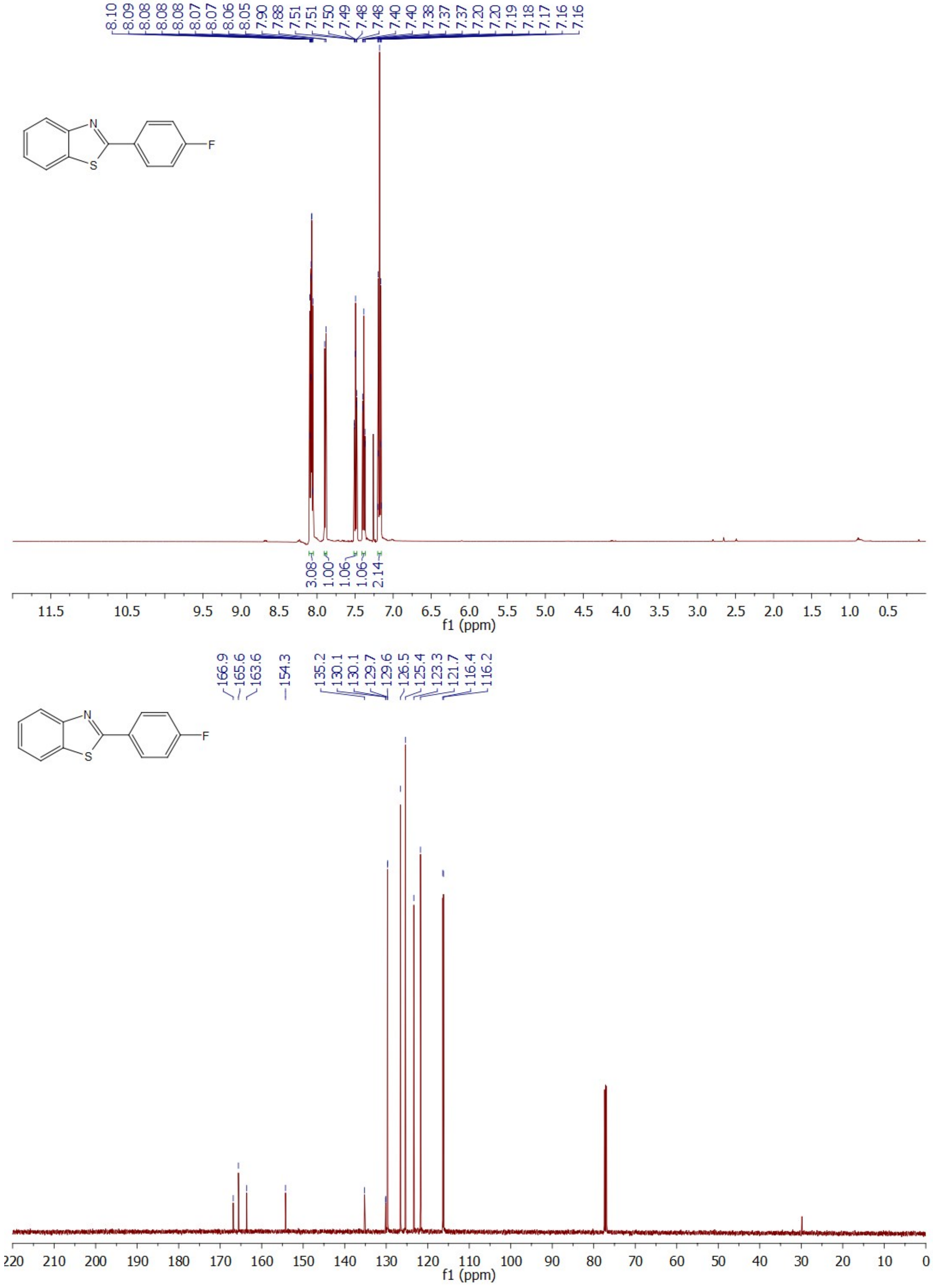

Figure 40. ^{1}H (top) and ^{13}C (bottom) NMR spectra of 2-(4-Fluorophenyl)benzothiazole

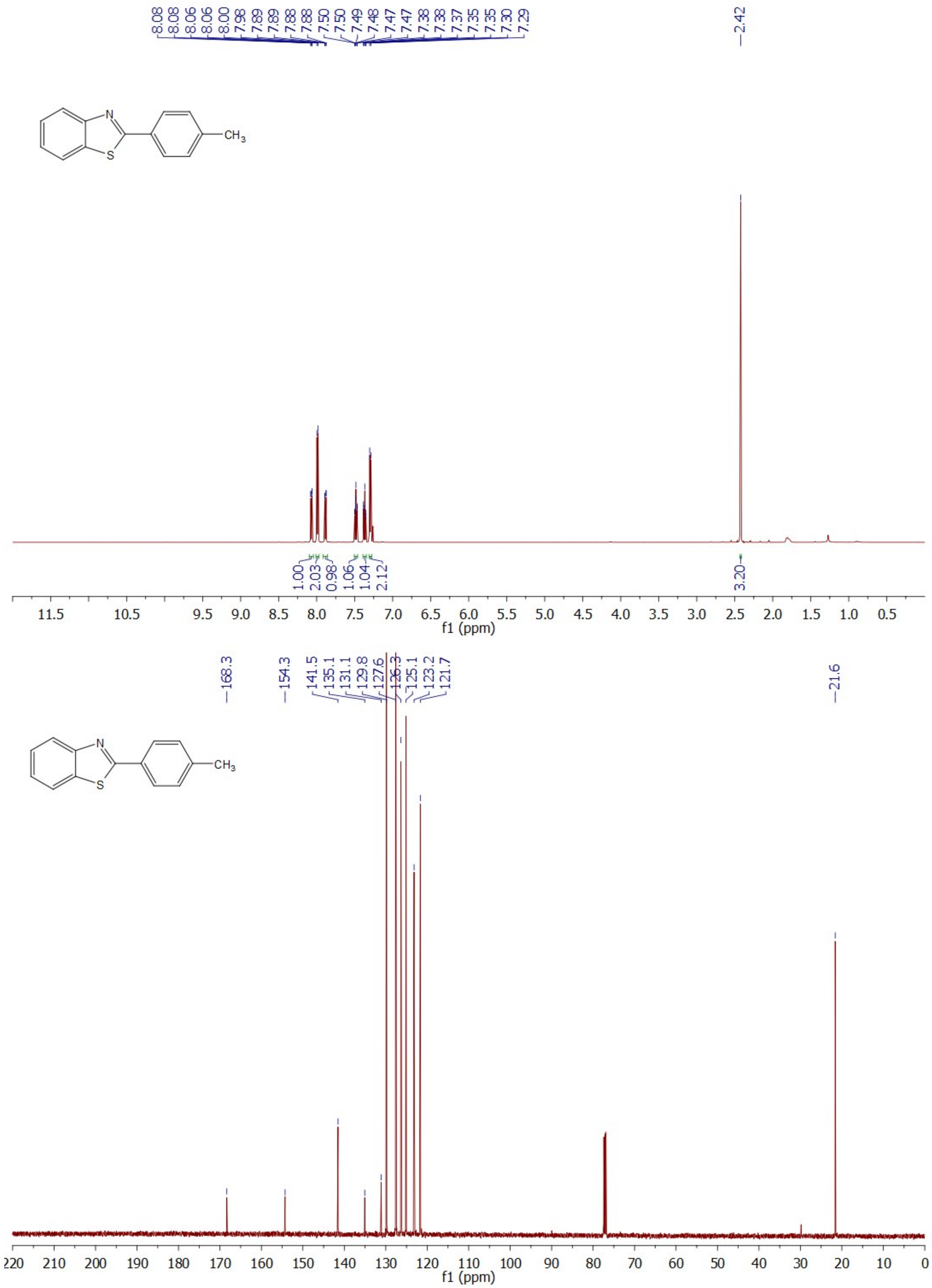

Figure 41. ^{1}H (top) and ^{13}C (bottom) NMR spectra of 2-(4-Methylphenyl)benzothiazole

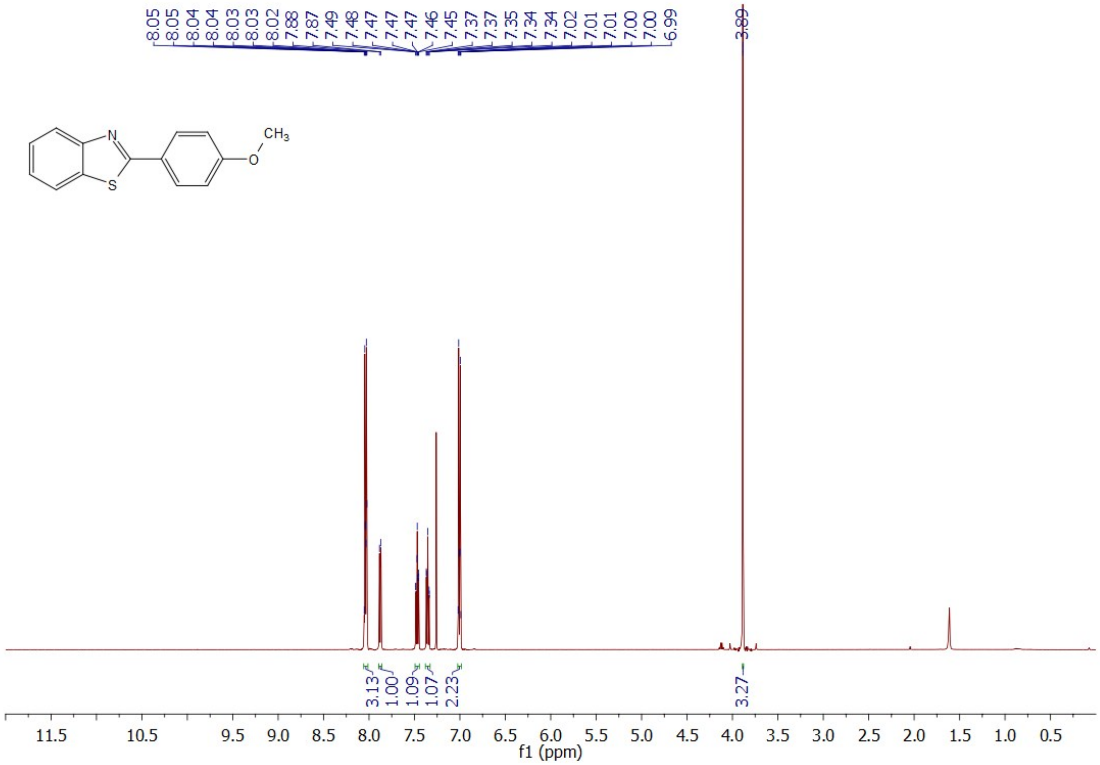

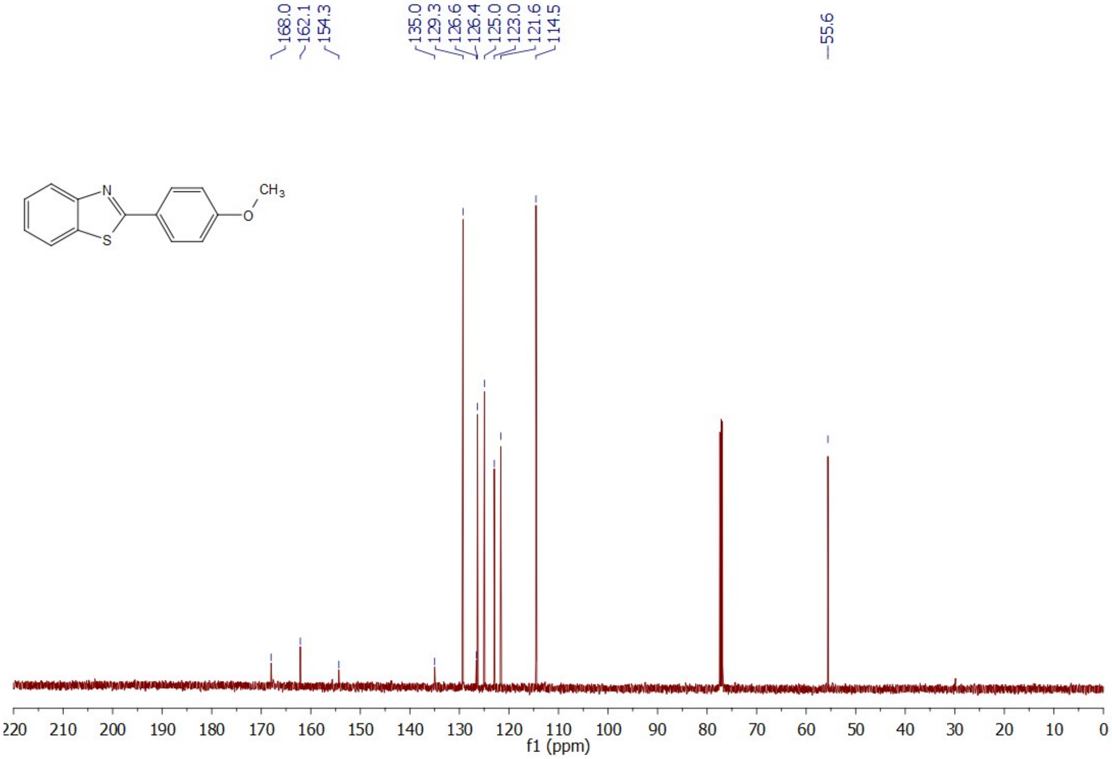

Figure 42. ^{1}H (top) and ^{13}C (bottom) NMR spectra of 2-(4-Methoxyphenyl)benzothiazole

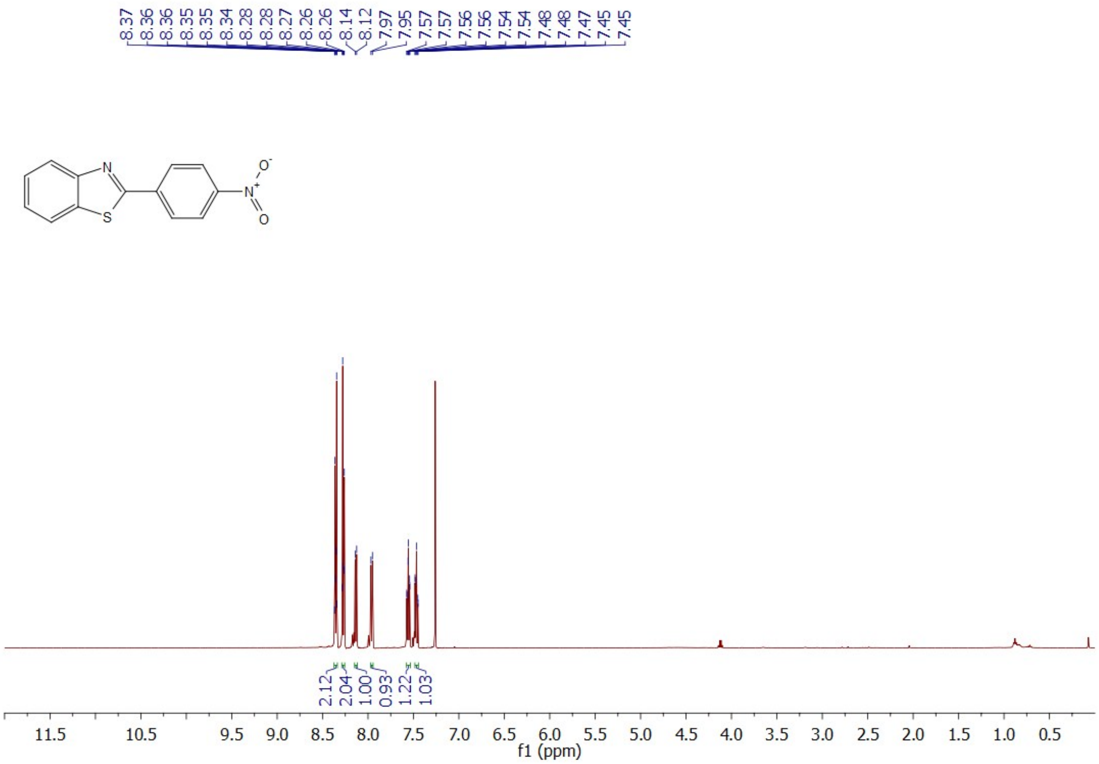

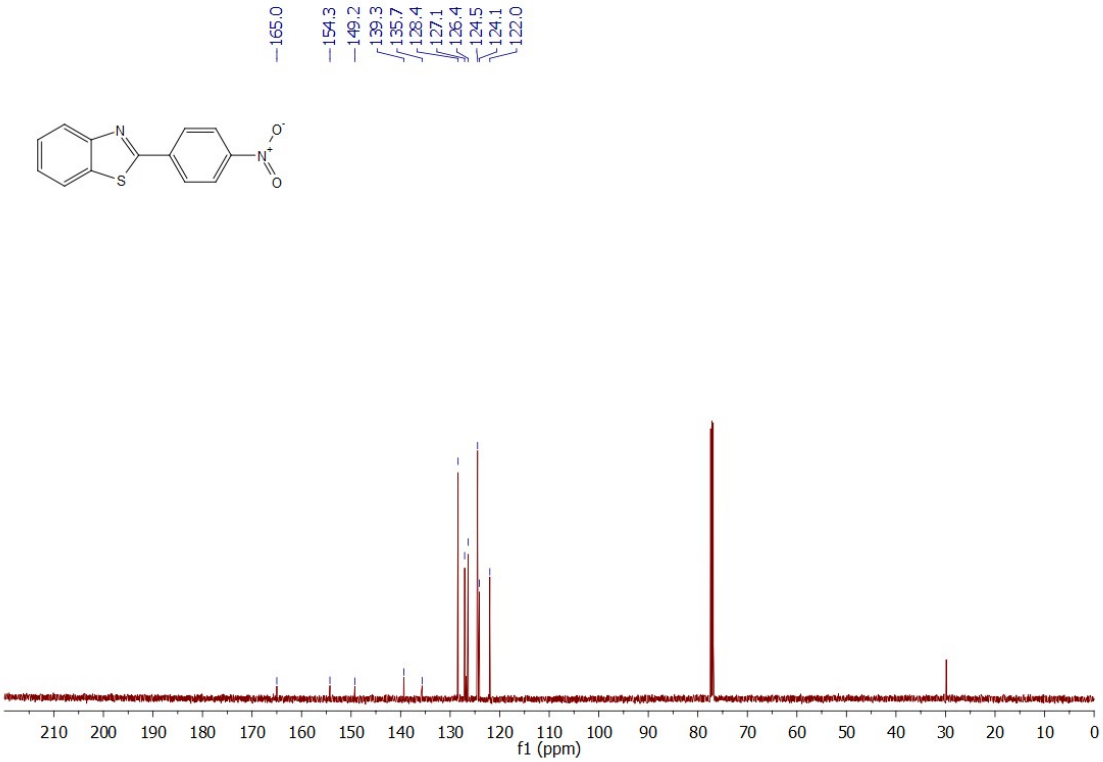

Figure 43. ^{1}H (top) and ^{13}C (bottom) NMR spectra of 2-(4-Nitrophenyl)benzothiazole
